# Supplementary material for: Bioactive Compounds of Porcine Hearts and Aortas May Improve Cardiovascular Disorders in Humans
Source: Int J Environ Res Public Health. 2021 Jul 8;18(14):7330. doi: 10.3390/ijerph18147330 (PMC8307898; doi:10.3390/ijerph18147330)
Supplement: Supplementary file 1 [file ijerph-18-07330-s001.zip › ijerph-1240964-Supplementary Materials.pdf]

Table S1. Fatty acids composition of developed MP.

№	trivial	systematic	index	*1*	*2*	*3*	*t, мин*
1	Caproic	Hexanoic	C 6:0		*0.01*		*4.42*
2	Caprylic	Octanoic	C 8:0	0.36	0.3	0.2	5.14
3	Capric	*Nonanoic*	C10:0	0.58	0.45	1.13	7.39
4		*Cis-9-decenoic*	C10:1	0.18			7.68
5		Undecanoic	C11:0	0.15	0.08	0.14	8.42
6	Lauric	Dodecanoic	C12:0	*0.22*	*0.17*	0.31	*9.29*
7		Tridecanoic	C13:0	*0.19*	*0.07*		*10.65*
8	Myristic	Tetradecanoic	C14:0	0.45	0.24	0.38	11.39
9	Myristoleic	Cis-9-tetradecenoic	C 14:1	0.24	0.17	0.15	11.55
10		Pentadecanoic	C15:0	0.61	0.49	0.33	12.37
11		cis-10-Pentadecenoic	C15:1		0.27		13.02
12	Palmitic	**Hexadecanoic**	**C16:0**	18.6	21.76	19.7	13.59
13	Palmitoleic	cis-9-hexadecenoic	C16:1	2.44	1.8	3.1	13.73
14		Heptadecanoic	C17:0	0.62	0.43	0.39	14.31
15		cis-10-heptadecenoic	C17:1	0.41	0.27	1.0	14.66
16	Stearic	**Octadecanoic**	**C18:0**	13.5	16.78	17.8	15.52
17	Oleic	Cis-9-**octadecenoic**	**C18:1**n9c	34.3	29.6	24.13	16.43
18		trans-9-octadecenoic	C18:1 n9t		0.23		16.66
19	LinoleicLA	Cis-9,12-octadecadienoic	C18:2 w6	9.6	14.5	13.45	16.97
20	y-linolenic GLA	cis-6,9,12-octadecatrienoic	C18:3 w6	0.14	0.09	0.25	17.95
21	a-linolenic	cis-9,12,15-octadecatrienoic	C18:3 w3		0.03	0.17	18.35
22		Nonadecanoic	C19:0		0.31		18.5
23		cis-11-eicosenoic	C20:1 w9		0.24		18.65
24		Eicosanoic	C20:0	0.18	0.15		19.29
25		cis-11,14-eicosadienoic	C20:2	0.26		0.35	20.20
26		*cis*-8,11,14-Eicosatrienoic	C20:3n6		0.24		20.55
27		Cis-11,14,17-eicosatrienoic	C20:3n3		0.11	0.2	21.50
28	Arachidonic	cis-5,8,11,14- Eicosatetraenoic	C20:4 w6	0.3	0.24		21.75
29		cis-5,8,11,14,17- Eicosapentaenoic	C20:5 ω3		0.06		22.25
30		Heneicosanoic	C21:0	0.4	0.48	0.12	22.87
31	Behenic	Docosanoic	22:0	1.38	1.2	0.83	23.3
32	Erucic	*cis*-13-docosenoic	C22:1n9	0.16			23.96
33		Cis-13,16,17-docosadienoic	C22:2	0.63	0.53		25.1
34		cis-5,8,11,14,17-docosapentaenoic	C22:5w3	0.1		0.08	25.53
35		*cis*-4,7,10,13,16,19-Docosahexaenoic	C22:6w3				26.01
36		Tricosanoic	C23:0				26.2
37	Lignoceric	Tetracosanoic	C24:0	0.42	0.63	0.33	24.45
38	nervonic	Cis-15-tetracosenoic	C24:1	0.6	0.34	0.52	28.13

Table S2. The results of search of peptide sequences identified in developed MP according to UniProt DataBase with species *Sus scrofa.*

No	Sequence	Result	Link
1	LCDFYNK	No entries matching your peptides found	https://www.uniprot.org/peptidesearch/uniprot/P20210423DA437993067D6F64326E5E763500BDED000413Y
2	LGADYYTK	No entries matching your peptides found	https://www.uniprot.org/peptidesearch/uniprot/P202104238471C63D39733769F8E060B506551E12001FD5M
3	VPYHLAAAR	No entries matching your peptides found	https://www.uniprot.org/peptidesearch/uniprot/P202104235C475328CEF75220C360D524E9D456CE0020A00
4	LEYFSSQK	No entries matching your peptides found	https://www.uniprot.org/peptidesearch/uniprot/P20210423DA437993067D6F64326E5E763500BDED000415U
5	LLAYTTKKK	No entries matching your peptides found	https://www.uniprot.org/peptidesearch/uniprot/P202104238BC4D7ADE02784B0C2481C7F3DE0963A0003E7X
6	LFDNYNTLK	No entries matching your peptides found	https://www.uniprot.org/peptidesearch/uniprot/P20210423A2A5A37CD3FF71F97605B695F360A9FA0004273
7	HNGN	212 proteins	https://www.uniprot.org/uniprot/?query=job:P202104238BC4D7ADE02784B0C2481C7F3DE0963A0003E83&columns=id,entry%20name,reviewed,protein%20names,genes,organism,length,peptidesearch(P202104238BC4D7ADE02784B0C2481C7F3DE0963A0003E83)
8	QGEEFCER	No entries matching your peptides found	https://www.uniprot.org/peptidesearch/uniprot/P202104238471C63D39733769F8E060B506551E12001FD69
9	WTCTQGPRWK	No entries matching your peptides found	https://www.uniprot.org/peptidesearch/uniprot/P20210423E5A08BB0B2D1C45B0C7BC3B55FD26556001FFB8
10	GLVDQGQHNCACR	No entries matching your peptides found	https://www.uniprot.org/peptidesearch/uniprot/P20210423216DA2B77BFBD2E6699CA9B6D1C41EB200204CW

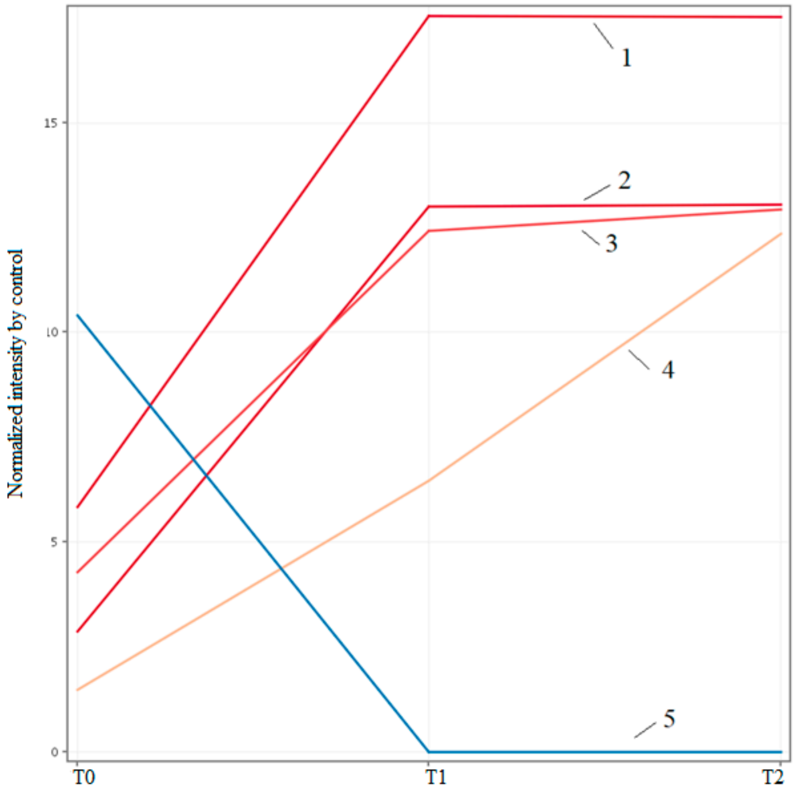

Figure S1. Changes in the dynamics of the peptide profile of the experimental group relative to the control group. 1 – KAAAAP; 2 – NLHLP; 3-organic substance No. 1; 4-organic substance No. 2; 5-FVAPW.

Table S3. The results of search of peptide sequences identified in human plasma according to UniProt DataBase with species *Sus scrofa.*

№	Protein	Protein No	Expression and tissue specificity	Function	Sequence	Similarities
**KAAAAP**						
1	LisH domain-containing protein	A0A4X1TTP7	No data	GO:0005515	Match to KAAAAP at 260<br>MADAGLRRVVPSDLYPLVLDFLRDNQLSDVANKFAKATGVTQQDANASSLLDIYSFWLKSTKAPKR<br>KLQANGPVTKKAKKKTSSSDSSEDSSEEEKAQGPPAKKAAAPAKRAGLPQQPGKATAKASESSSSSS<br>EESSDEEEDKKKKPVQKVVKPQGKAVKAPPKKAESSDSDSDSSSEDEAPKNQKPKTAAVAAKAQAK<br>VPAKTGTPARAAPKVANGKAASSSSSSSSSDDDSEEEKAAAVSKKTVPKKQVVVKAPVKAAAAPAQ<br>KSSSSEDSSSEEEEEEQKKKPMKKKPGTYSSVPPPSVPSPKKPLGTQAPKKAAGKQEPVESSEDSSDESD<br>SSSEEEKKPPAKAVVPKAATKAAPAKKAAESSSDSSDSDSSEDEAPAKPASATKNSSKPAATPKQSIAK<br>PATSSKQPVGSGQKPLTRKADSSSSEEESSSSEDEKMKKTVATPKSKVTAKAAPSLPAKQASQGGGDSS<br>SDSDSSSSEEEEEEKTSKTPAKKMPQKDVGAVAPSKPASAKQAKAESSSSSSSDDSSEEEEEDKPKGKST<br>PKPQAPKANGTTALTAQNGKADRDSNEEEEDKKKAAVGVSKPGSGKKRKQNEAAKETETPPAKKIK<br>PQTPNTFPKRKKGERRGSSPFRRIREEEIEVDARVADNSFDAKRGAAGDWGERANQVLKFTKGKSFR<br>HEKTKKKRGSYRGGSISVQVNSIKFDSE	
2	Dymeclin	A0A286ZZM5	Low in heart left ventricle	GO:0007030	Match to KAAAAP at 269<br>MGSNSSRIGDLPKNEYLKKLSGTESVSENDPFWNQLLSFSPAPTSSTELKLLEEATISVCRSLVENNPRT<br>GNLGALIKVFLSRTKELKLSAECQNHIFIWQTHNALFIICCLLKVFICEMSEEELQLHFTYEEKSPGSYSS<br>DSEDLLEELLCCLMQLITDIPLLDITYEISVEAVSTMVVFLSCQLFHKEVLRQSISHKYLMRGRCLPYTSK<br>LVKTLLYNFIRQEKPPPPGAHVLPQQSDSGGLLYGLASGVATGLWTVFTLGGVGSKAAAAPELSSPLA<br>NQSLLLLLVLANLTDAADAPNPYRQAIMSFKNTQDSSPFPSSNPHAFQINFNSLYMALCEQQTSDQA<br>TLLLYTLLHQNSNIRTYMLARTDMENLILKNITWYSERVLTEISLGSLLILVIRTIQYNMTRTRDKYLHT<br>NCLAALANMSAQFRSLHQYAAQRIISLFSLLSKKHNKLEQATQSLRGSLSSNDVPLPDYAQDLNVIEE<br>VIRMMLEIINSCLTNSLHHNPNLVYALLYKRDLFEQFRTHPSFQDIMQNIDLVITFFSSRLLQAGAELSV<br>ERVLEIIKQGVVALPKDRLKKFPELKFKYVEEEQPEEFFIPYVWSLVYNTAVGLYWNPQDIQLFTMDSD	
3	Dymeclin	A0A4X1UIA2	No data	No data	Match to KAAAAP at 105<br>HDITYEISVAVSTMVVFLSCQLFHKEVLRQSISHKYLMRGRCLPYTSKLVKTLLYNFIRQEKPPPPGAH<br>VLPQQSDSGGLLYGLASGVATGLWTVFTLGGVGSKAAAAPELSSPLANQSLLLLLVLANLTDAADAP<br>NPYRQAIMSFKNTQDSSPFPSSNPHAFQINFNSLYMALCEQQTSDQATLLLYTLLHQNSNIRTYMLAR<br>TDMENLIWIIPFLTSSHVYLNFSSPLQILKNITWYSERVLTEISLGSLLILVVIRTIQYNMTRTRDKYLHTN<br>CLAALANMSAQFRSLHQYAAQRIISLFSLLSKKHNKVLEQATQSLRGSLSSNDVPLPDYAQDLNVIEE<br>VIRMMLEIINSCLTNSLHHNPNLVYALLYKRDLFEQFRTHPSFQDIMQNIDLVITFFSSRLLQAGAELSV<br>ERVLEIIKQGVVALPKDRLKKFPELKFKYVEEEQPEEFFIPYVWSLVYNTAVGLYWNPQDIQLFTMDSD	
4	Dymeclin	A0A5G2R050	No data	No data	Match to KAAAAP at 79<br>MGSNSSRIGDLPKNEYLKKLSGTESVSENDPFWNQLLSFSFPAPTSSTELKLLEEATISVCRSLAGLWTV<br>FTLGGVGSKAAAAPELSSPLANQSLLLLLVLANLTDAADAPNPYRQAIMSFKNTQDSSPFPSSNPHAF<br>QINFNSLYMALCEQQTSDQATLLLYTLLHQNSNIRTYMLARTDMENLVLPILEILYHVEERNSHHVY<br>MALIILLILTEDDGFNRSIHEVILKNITWYSERVLTEISLGSLLILVVIRTIQYNMTRTRDKYLHTNCLAAL<br>ANMSAQFRSLHQYAAQRIISLFSLLSKKHNKVLEQATQSLRGSLSSNDVPLPDYAQDLNVIEEVIRMM<br>LEIINSCLTNSLHHNPNLVYALLYKRDLFEQFRTHPSFQDIMQNIDLVITFFSSRLLQAGAELSVERVLEI<br>IKQGVVALPKDRLKKFPELKFKYVEEEQPEEFFIPYVWSLVYNT AVGLYWNPQDIQLFTMDSD	
5	Dymeclin	A0A5G2R786	No data	No data	Match to KAAAAP at 269<br>MGSNSSRIGDLPKNEYLKKLSGTESVSENDPFWNQLLSFSFPAPTSSTELKLLEEATISVCRSLVENNPR<br>TGNLGALIKVFLSRTKELKLSAECQNHIFIWQTHNALFIICCLLKVFICEMSEEELQLHFTYEEKSPGSYS	

					SDSEDLLEELLCCLMQLITDIPLLDITYEISVEAVSTMVVFLSCQLFHKEVLRQSISHKYLMRGRCLPYTS<br>KLVKTLLYNFIRQEKPPPPGAHVLPQQSDSGGLLYGLASGVATGLWTVFTLGGVGSKAAAAPELSSPL<br>ANQSLLLLLVLANLTDAADAPNPYRQAIMSFKNTQDSSPFPSSNPHAFQINFNSLYMALCEQQTSDQ<br>ATLLLYTLLHQNSNIRTYMLARTDMENLVLPILEILYHVEERNSHHVYMALIILLILTEDDGFNRSIHEV<br>ILKNITWYSERVLTEISLGSLLILVVIRTIQYNMTRTRDKYLHTNCLAALANMSAQFRSLHQYAAQRIIS<br>LFSLLSKKHNKVLEQATQSLRGSLSSNDVPLPDYAQDLNVIEEVIRMMLEIINSCLTNSLHHNPNLVY<br>ALLYKRDLFEQFRTHPSFQDIMQNIDLVITFFSSRLLQAGAELSVERVLEIIKQGVVALPKDRLKKRLSE<br>YGLRYRSDTQKCNEGF	
6	Dymeclin	F1RPR0	High expression level in right coronary artery, left ventricle free wall, heart left ventricle, coronary artery, heart ventricle, cardiac muscle (myocardium)	No data	Match to KAAAAP at 269<br>MGSNSSRIGDLPKNEYLKKLSGTESVSENDPFWNQLLSFSFPAPTSSTELKLLEEATISVCRSLVENNPR<br>TGNLGALIKVFLSRTKELKLSAECQNHIFIWQTHNALFIICCLLKVFICEMSEEELQLHFTYEEKSPGSYS<br>SDSEDLLEELLCCLMQLITDIPLLDITYEISVEAVSTMVVFLSCQLFHKEVLRQSISHKDSGGLLYGLASG<br>VATGLWTVFTLGGVGSKAAAAPELSSPLANQSLLLLLVLANLTDAADAPNPYRQAIMSFKNTQDSSP<br>FPSSNPHAFQINFNSLYMALCEQQTSDQATLLLYTLLHQNSNIRTYMLARTDMENLVLPILEILYHVEE<br>RNSHHVYMALIILLILTEDDGFNRSIHEDKYLHTNCLAALANMSAQFRSLHQYAAQRIISLFSLLSKKH<br>NKVLEQATQSLRGSLSSNDVPLPDYAQDLNVIEEVIRMMLEIINSCLTNSLHHNPNLVYALLYKRDLF<br>EQFRTHPSFQDIMQNIDLVITFFSSRLLQAGAELSVERVLEIIKQGVVALPKDRLKKFPELKFKYVEEEQ<br>PEEFFIPYVWSLVYNTAVGLYWNPQDIQLFTMDSD	90% with A0A4X1UIA2 (3)
7	Dymeclin	I3LV52	Low in heart left ventricle	No data	Match to KAAAAP at 269<br>MGSNSSRIGDLPKNEYLKKLSGTESVSENDPFWNQLLSFSFPAPTSSTELKLLEEATISVCRSLVENNPR<br>TGNLGALIKVFLSRTKELKLSAECQNHIFIWQTHNALFIICCLLKVFICEMSEEELQLHFTYEEKSPGSYS<br>SDSEDLLEELLCCLMQLITDIPLLDITYEISVEAVSTMVVFLSCQLFHKEVLRQSISHKYLMRGRCLPYTS<br>KLVKTLLYNFIRQEKPPPPGAHVLPQQSDSGGLLYGLASGVATGLWTVFTLGGVGSKAAAAPELSSPL<br>ANQSLLLLLVLANLTDAADAPNPYRQAIMSFKNTQDSSPFPSSNPHAFQINFNSLYMALCEQQTSDQ<br>ATLLLYTLLHQNSNIRTYMLARTDMENLVLPILEILYHVEERNSHHVYMALIILLILTEDDGFNRSIHEV<br>ILKNITWYSERVLTEISLGSLLILVVIRTIQYNMTRTRDKYLHTNCLAALANMSAQFRSLHQYAAQRIIS<br>LFSLLSKKHNKVLEQATQSLRGSLSSNDVPLPDYVITFFSSRLLQAGAELSVERVLEIIKQGVVALPKDR<br>LKKFPELKFKYVEEEQPEEFFIPYVWSLVYNTAVGLYWNPQDIQLFTMDSD	
8	Dymeclin	A0A5G2QRC1	No data	No data	Match to KAAAAP at 269<br>MGSNSSRIGDLPKNEYLKKLSGTESVSENDPFWNQLLSFSFPAPTSSTELKLLEEATISVCRSLVENNPR<br>TGNLGALIKVFLSRTKELKLSAECQNHIFIWQTHNALFIICCLLKVFICEMSEEELQLHFTYEEKSPGSYS<br>SDSEDLLEELLCCLMQLITDIPLLDITYEISVEAVSTMVVFLSCQLFHKEVLRQSISHKYLMRGRCLPYTS<br>KLVKTLLYNFIRQEKPPPPGAHVLPQQSDSGGLLYGLASGVATGLWTVFTLGGVGSKAAAAPELSSPL<br>ANQSLLLLLVLANLTDAADAPNPYRQAIMSFKNTQDSSPFPSSNPHAFQINFNSLYMALCEQQTSDQ<br>ATLLLYTLLHQNSNIRTYMLARTDMENLVLPILEILYHVEERNSHHVYMALIILLILTEDDGFNRSIHEV<br>ILKNITWYSERVLTEISLGSLLILVVIRTIQYNMTRTRDKYLHTNCLAALANMSAQFRSLHQYAAQRIIS<br>YTCRHLRRYVYVLDKLYFPHSHCSTLQHCFSTLSDNGEELLSLTCSHILRSYASSLFSLLSKKHNKVLEQ<br>ATQSLRGSLSSNDVPLPDYAQDLNVIEEVIRMMLEIINSCLTNSLHHNPNLVYALLYKRDLFEQFRTHP<br>SFQDIMQNIDLVITFFSSRLLQAGAELSVERVLEIIKQGVVALPKDRLKKFPELKFKYVEEEQPEEFFIPY<br>VWSLVYNTAVGLYWNPQDIQLFTMDSD	
9	Dymeclin	A0A480JQY8	No data	No data	Match to KAAAAP at 269<br>MGSNSSRIGDLPKNEYLKKLSGTESVSENDPFWNQLLSFSFPAPTSSTELKLLEEATISVCRSLVENNPR<br>TGNLGALIKVFLSRTKELKLSAECQNHIFIWQTHNALFIICCLLKVFICEMSEEELQLHFTYEEKSPGSYS<br>SDSEDLLEELLCCLMQLITDIPLLDITYEISVEAVSTMVVFLSCQLFHKEVLRQSISHKYLMRGRCLPYTS<br>KLVKTLLYNFIRQEKPPPPGAHVLPQQSDSGGLLYGLASGVATGLWTVFTLGGVGSKAAAAPELSSPL	90% with A0A5G2R786 (5)

					ANQSLLLLLVLANLTDAADAPNPYRQAIMSFKNTQDSSPFPSSNPHAFQINFNSLYMALCEQQTSDQ<br>ATLLLYTLLHQNSNIRTYMLARTDMENLVLPILEILYHVEERNSHHVYMALIILLILTEDDGFNRSIHEV<br>ILKNITWYSERVLTEISLGSLLILVVIRTIQYNMTRTRDKYLHTNCLAALANMSAQFRSLHQYAAQRIIS<br>LFSLLSKKHNKVLEQATQSLRGSLSSNDVPLPDYAQDLNVIEEVIRMMLEIINSCLTNSLHHNPNLVY<br>ALLYKRDLFEQFRTHPSFQDIMQNIDLVITFFSSRLLQAGAELSVERVLEIIKQGVVALPKDRLKKFPEL<br>KFKYVEEEQPEEFFIPYVWSLVYNTAVGLYWNPQDIQLFTMDSD	
10	Dymeclin	I3LFB3	High in heart	No data	Match to KAAAAP at 269<br>MGSNSSRIGDLPKNEYLKKLSGTESVSENDPFWNQLLSFSFPAPTSSTELKLLEEATISVCRSLVENNPR<br>TGNLGALIKVFLSRTKELKLSAECQNHIFIWQTHNALFIICCLLKVFICEMSEEELQLHFTYEEKSPGSYS<br>SDSEDLLEELLCCLMQLITDIPLLDITYEISVEAVSTMVVFLSCQLFHKEVLRQSISHKYLMRGRCLPYTS<br>KLVKTLLYNFIRQEKPPPPGAHVLPQQSDSGGLLYGLASGVATGLWTVFTLGGVGSKAAAAPELSSPL<br>ANQSLLLLLVLANLTDAADAPNPYRQAIMSFKNTQDSSPFPSSNPHAFQINFNSLYMALCEQQTSDQ<br>ATLLLYTLLHQNSNIRTYMLARTDMENLVLPILEILYHVEERNSHHVYMALIILLILTEDDGFNRSIHEV<br>ILKNITWYSERVLTEISLGSLLILVVIRTIQYNMTRTRDKYLHTNCLAALANMSAQFRSLHQYAAQRIIS<br>LFSLLSKKHNKVLEQATQSLRGSLSSNDVPLPDYAQDLNVIEEVIRMMLEIINSCLTNSLHHNPNLVY<br>ALLYKRDLFEQFRTHPSFQDIMQNIDLVITFFSSRLLQAGAELSVERVLEIIKQGVVALPKDRLKAGPGD<br>SPTQRHQEVKERMHLVVLKP	90% with A0A5G2R786 (5) A0A480JQY8 (9)
11	Dymeclin	A0A4X1UIA6	No data	GO:0016021	Match to KAAAAP at 115<br>MYSLLEKRTHLSAKVIIIPLSVSTMVVFLSCQLFHKEVLRQSISHKYLMRGRCLPYTSKLVKTLLYNFIRQ<br>EKPPPPGAHVLPQQSDSGGLLYGLASGVATGLWTVFTLGGVGSKAAAAPELSSPLANQSLLLLLVLA<br>NLTDAADAPNPYRQAIMSFKNTQDSSPFPSSNPHAFQINFNSLYMALCEQQTSDQATLLLYTLLHQN<br>SNIRTYMLARTDMENLIWIIPFLTSSHVYLNFSSPLQILKNITWYSERVLTEISLGSLLILVVIRTIQYNMT<br>RTRDKYLHTNCLAALANMSAQFRSLHQYAAQRIISLFSLLSKKHNKVLEQATQSLRGSLSSNDVPLPD<br>YAQDLNVIEEVIRMMLEIINSCLTNSLHHNPNLVYALLYKRDLFEQFRTHPSFQDIMQNIDLVITFFSSR<br>LLQAGAELSVERVLEIIKQGVVALPKDRLKKFPELKFKYVEEEQPEEFFIPYVWSLVYNTAVGLYWNPQ<br>DIQLFTMDSD	90% with A0A4X1UIA2 (3)
12	E1A binding protein p400	I3L7Y4	Medium-low in heart (64.22) and in endocardial endothelium (58.11)	GO:0005524 GO:0070615 GO:0043968 GO:0043967	Match to KAAAAP at 1599<br>MLFLYPRPFPPLLLRSQPFFPRLQDPAQHTALPTRPAHFPAPRPAQGVRKHQSPQPTLPPRLAGPTHR<br>GSELSSRAYSRSSKGAPEGTDGHTQPAPSPLELPSRNLHSPQTPRLARRRPIGPPRRLASLAFLAVSTPPS<br>HPTSCRAPKRSCGRAFLPARRSRRAAASSAQVPDVSSGTAPPGPRPQPQFDAEEQAGPGRTLSWIKRR<br>AIMHHGSGPQNVQHQLQRSRAFASSEGEEQPAHPNPPQSPATPFAPSASPSAPQSPGYPVQQLMSRS<br>PVAGQNVNIALQNVGPVVGGNPQITLAPLPLPSPTSPGFQFSTQQRRFEHGSPSYIQVTSPLSQQVQTQ<br>SPTQPSPGPGPGLQSVRAGAPGPGLGLCSSSPTGGFVDASVLVRQISLSPSSGSHFVFQEGSGLAQMAQ<br>GGQVQLQHAGAPIAVRERRLSQPHAQSGGTVHHLGPQSPAGGAGLQPLSSPGHITTTSLPPQISSIIQG<br>QLIQQQQVLQGPPLTRPLLPGVGVGVGGASAFGMTSPPPPTSPSRTAVPPGLSSLPLTSAGSAGVRKAP<br>RKLEEIPPASQELAQMRKQCLDYHYREMEALKEAFKEYLIELFFLQHLQGNMMDFLAFKKKHYAPL<br>QAYLRQNDLDLEEEEEEEEEEEKSEVINDEQQALAGTLVAGAGSTVETDPFKRQQAGPPAEQSKRPRL<br>EVGHQGVVFQHPGVNTGVPLQQLMPTVQGGMPPAPQAAQLAGQKQSQQQYDPSTGPPVQNAASL<br>HTPPPQLPGRLPAAGLPTVPLPPALQFSQQPQMVEPQPQLQIPVKTQQPNAPVPAPPPSQLPAPPPQP<br>TPPALHVPMPGKAQMQAPPLSAQPQTVASSRPPVDPAQPCPRPPPSSSTSSIAPASGSGPGPSPARASP<br>VNRPSLATTKSLSPVTSRSPGAAVSALPKPQSPAQNTAPPQDSSQDKLAEQIALENQIHQRIADLRKEG<br>LWSLRRLPKLQEAPRPKSHWDYLLEEMQWMATDFAQERRWKVAAAKKLVRTVARHHEEKKLREE<br>RGKKEEQNRLRRIAASTAREIEYFWSNIEQVVEIKLQVELEEKRKKALHLQKVSRKGTSFFLQPFLAELP<br>LIDLMKLYEGAFLPNFQWPQPKPDSDETSEEEDMEDCSGDRESRKDVVLIDSLFIMDQFKAAERVNV<br>GKPHTKDIAEVTAVAEAVLPKGSARISTGVKLTAPALLYGALRDYQKIGLDWLAKLYRKNLNGILAD<br>EAGLGKTVQIIAFFAHLACNEGNWGPHLVIVRSCNILKWELELKRWCPGLKTLLYVGSHRELKAKRQ<br>EWSEPNSFNICITSYKQFFRGHASFPRARWKCLVIDEMQRVKGMTERHWEAVFALQSQQRLLLIDAP<br>LHNTFLELWTMVHFLIPGISRPYLHFPLKAPSEENQDYYHKVVIRLHRVTQPFILRRTKRDVEKQLTKK	

					YEHVLKCRLSNRQKALYEDVILQPGTQEALKSGHFVDVLRPLQYRSASLILKALDRDFWKETDLSIFDL<br>IGLENKMTHHEAELLCKKKVTRKLFEELFTAPPLPSSGRPVPVRLKPSRLFQPVQYGQKPEGRTVAFPS<br>AHPPRMAASATATAAPQGHVRGRPPIATFSANPDAKAAAAPFQTSQASSGAPRHQPASTFSTAPSPA<br>HPAKLRAPGPPPPQPQAPSHPAGQSALPQGLVLTSQAQARLPSGEVVKIAQLASLAGPPSRVAQPETP<br>VTLQFQGNKFTLSHSQLRQLTAGQPLQLQGSVLQIVSAPGQSYLRPPGPVVMQTVSQAGALNALGSK<br>PPAGGPGPTPVTPPVAVPGRVAVSPLAAGEPGVASKPASPVAGPTQEEKTRLMKERLDQIYFVNERRC<br>SRAPIYGRDLLGVCSLMGREHMPWLRASESSTGKVTGPVSQFISPSKSQRDLILTLTQRQESLQDVINRV<br>VCVIPPVVAAPPRLWVARPPSLYSHKMRLFRHGLQEHTAPYAQQLQQMTALRSLQFPELRLVQFDSG<br>KLEALAVLLQKLRSEGRRVLILSQMVLMLDILEMFLNFHYLTYIRIDENANSEQRQELMRSFNRDRRIF<br>CALLSTHSRTTGVSLVEADAVVFYDNDLNPVMDAKAQEWCDRIGRRRDVHIYRLVSGNSIEEKLLKN<br>GTKDLIREVAAQGNDYSMAFLTQRTIQELFEVYSPMDDAGFPVKAEEFVVLSQEPSVTETIAPKIARPFI<br>EALKSIEYLEEDAQKPFEEAVPEVSDSDSSRWNEEPSQLEELADFMGQLTPIEKYALNYLELFHTSIDQE<br>KERNSEGAVLSAVQEWEARNARMLREQEARELREQEQEQLLTYTREDAYNAEYVCEGAEGQTEVM<br>PLWTPPTPPQDDNDIYIDSVMCLMYETTPIPESKLPPVYVRKERRRHKTDPSAAGRKKKQRHGEAVVP<br>PRSLFDRATPGMLKIRREGKEQKKNILLKQQTQFAKPLPTFAKPTAESGPDNPEWLISEDWALLQAVK<br>QLLELPLNLTIVSPAHTPNWDLVSDVVNSCSRIYRSSKQCRNRYENVLIPREEGKSKNNRPLRTGQMY<br>AQDENATHTQLYTSHFDLMKMTAGKRSPPIKPLLGMNPFQKNPKHASVLAESGINYDKPLPPIQVAS<br>LRAERIAKEKKALADQQKAQQPPVAQPPPPPPQPQPPPPQQPPPPLPQPQATSSQQPTGPPAVQPQA<br>QAQPPAQPTQPPPKAPPAITTVGSAAVLQAGAIKTSVTGTSMPTGTVSGNVIVNTIAGVPAATFQSIN<br>KRLASPVAPGALTTSGGSAPAQVVHTQPRAVGSPATAASDLVSLAPTQGVRAVTSVTASAVVTTSLTP<br>VQTPTRSLVTQVSQATGVQLPGKTITPAHFQLLRQQQQQQQQQQQQQQQQQAASQVQVPQIQGQ<br>AQSPAQIKAVGKLTPEHLIKMQKQKLQLPQQAPPPQAPPGPPQPTAQVQVQPPQPTQQQSPQLTTVT<br>APRPGALLTGTTVANLQVARLTRVPTSQLQAQGQMQAQAPQPAQVALAKPPVVSVPAAVVSSPGV<br>TTLPMNVAGISVAIGQPQKTAGQTVVAQPVHVQQLLKLKQQAVQQQKAIQPQAAPGPAAVQQKIT<br>AQQIAAQGQAQKVTYATQPALKTQFLTTPISQAQKLAGTQQVQTQIQVAKLPQVVQQQTPVASIQQ<br>VASASQQASPQTVTLTQATAAGQQVQMIPAVTATAQVVQQKLMQQQVVTTAAAQLQTPGVPNPA<br>QAPASADSPNQQPKLQMRVPAVRLKTPTKPPCP	
13	E1A binding protein p400	A0A287AHU7	Low in heart left ventricle	The same (12) + GO:0006325	Match to KAAAAP at 1562<br>MLFLYPRPFPPLLLRSQPFFPRLQDPAQHTALPTRPAHFPAPRPAQGVRKHQSPQPTLPPRLAGPTHR<br>GSELSSRAYSRSSKGAPEGTDGHTQPAPSPLELPSRNLHSPQTPRLARRRPIGPPRRLASLAFLAVSTPPS<br>HPTSCRAPKRSCGRAFLPARRSRRAAASSAQVPDVSSGTAPPGPRPQPQFDAEEQAGPGRTLSWIKRR<br>AIMHHGSGPQNVQHQLQRSRAFASSEGEEQPAHPNPPQSPATPFAPSASPSAPQSPGYPVQQLMSRS<br>PVAGQNVNIALQNVGPVVGGNPQITLAPLPLPSPTSPGFQFSTQQRRFEHGSPSYIQVTSPLSQQVQTQ<br>SPTQPSPGPGPGLQSVRAGAPGPGLGLCSSSPTGGFVDASVLVRQISLSPSSGSHFVFQEGSGLAQMAQ<br>GGQVQLQHAGAPIAVRERRLSQPHAQSGGTVHHLGPQSPAGGAGLQPLSSPGHITTTSLPPQISSIIQG<br>QLIQQQQVLQGPPLTRPLLPGVGVGVGGASAFGMTSPPPPTSPSRTAVPPGLSSLPLTSAGSAGVRKAP<br>RKLEEIPPASQELAQMRKQCLDYHYREMEALKEAFKEYLIELFFLQHLQGNMMDFLAFKKKHYAPL<br>QAYLRQNDLDLEEEEEEEEEEKSEVINDEVRMCGVLSEGVYDSLEPQSQCRGGLSSFAGGMPPAPQA<br>AQLAGQKQSQQQYDPSTGPPVQNAASLHTPPPQLPGRLPAAGLPTVPLPPALQFSQQPQMVEPQPQ<br>LQIPVKTQQPNAPVPAPPPSQLPAPPPQPTPPALHVPMPGKAQMQAPPLSAQPQTVASSRPPVDPAQ<br>PCPRPPPSSSTSSIAPASGSGPGPSPARASPVNRPSLATTKSLSPVTSRSPGAAVSALPKPQSPAQNTAPP<br>QDSSQDKLAEQIALENQIHQRIADLRKEGLWSLRRLPKLQEAPRPKSHWDYLLEEMQWMATDFAQE<br>RRWKVAAAKKLVRTVARHHEEKKLREERGKKEEQNRLRRIAASTAREIEYFWSNIEQVVEIKLQVELE<br>EKRKKALHLQKVSRKGTSFFLQPFLAELPLIDLMKLYEGAFLPNFQWPQPKPDSDETSEEEDMEDCSG<br>DRESRKDVVLIDSLFIMDQFKAAERVNVGKPHTKDIAEVTAVAEAVLPKGSARISTGVKLTAPALLYG<br>ALRDYQKIGLDWLAKLYRKNLNGILADEAGLGKTVQIIAFFAHLACNEGNWGPHLVIVRSCNILKW<br>ELELKRWCPGLKTLLYVGSHRELKAKRQEWSEPNSFNICITSYKQFFRGHASFPRARWKCLVIDEMQR<br>VKGMTERHWEAVFALQSQQRLLLIDAPLHNTFLELWTMVHFLIPGISRPYLHFPLKAPSEENQDYYH<br>KVVIRLHRVTQPFILRRTKRDVEKQLTKKYEHVLKCRLSNRQKALYEDVILQPGTQEALKSGHFVDVL	90% with I3L7Y4 (12)

					RPLQYRSASLILKALDRDFWKETDLSIFDLIGLENKMTHHEAELLCKKKVTRKLFEELFTAPPLPSSGRP<br>VPVRLKPSRLFQPVQYGQKPEGRTVAFPSAHPPRMAASATATAAPQGHVRGRPPIATFSANPDAKAA<br>AAPFQTSQASSGAPRHQPASTFSTAPSPAHPAKLRAPGPPPPQPQAPSHPAGQSALPQGLVLTSQAQ<br>ARLPSGEVVKIAQLASLAGPPSRVAQPETPVTLQFQGNKFTLSHSQLRQLTAGQPLQLQGSVLQIVSAP<br>GQSYLRPPGPVVMQTVSQAGALNALGSKPPAGGPGPTPVTPPVAVPGRVAVSPLAAGEPGVASKPAS<br>PVAGPTQEEKTRLMKERLDQIYFVNERRCSRAPIYGRDLLGVCSLMGREHMPWLRASESSTGKVTGP<br>VSQFISPSKSQRDLILTLTQRQESLQDVINRVVCVIPPVVAAPPRLWVARPPSLYSHKMRLFRHGLQEH<br>TAPYAQQLQQMTALRSLQFPELRLVQFDSGKLEALAVLLQKLRSEGRRVLILSQMVLMLDILEMFLNF<br>HYLTYIRIDENANSEQRQELMRSFNRDRRIFCALLSTHSRTTGVSLVEADAVVFYDNDLNPVMDAKA<br>QEWCDRIGRRRDVHIYRLVSGNSIEEKLLKNGTKDLIREVAAQGNDYSMAFLTQRTIQELFEVYSPMD<br>DAGFPVKAEEFVVLSQEPSVTETIAPKIARPFIEALKSIEYLEEDAQKPFEEAVPEVSDSDSSRWNEEPSQ<br>LEELADFMGQLTPIEKYALNYLELFHTSIDQEKERNSEGAVLSAVQEWEARNARMLREQEARELREQ<br>EQEQLLTYTREDAYNAEYVCEGAEGQTEVMPLWTPPTPPQDDNDIYIDSVMCLMYETTPIPESKLPPV<br>YVRKERRRHKTDPSAAGRKKKQRHGEAVVPPRSLFDRATPGMLKIRREGKEQKKNILLKQQTQFAKP<br>LPTFAKPTAESGPDNPEWLISEDWALLQAVKQLLELPLNLTIVSPAHTPNWDLVSDVVNSCSRIYRSSK<br>QCRNRYENVLIPREEGKSKNNRPLRTGQMYAQDENATHTQLYTSHFDLMKMTAGKRSPPIKPLLGM<br>NPFQKNPKHASVLAESGINYDKPLPPIQVASLRAERIAKEKKALADQQKAQQPPVAQPPPPPPQPQPP<br>PPQQPPPPLPQPQATSSQQPTGPPAVQPQAQAQPPAQPTQPPPKAPPAITTVGSAAVLQAGAIKTSVT<br>GTSMPTGTVSGNVIVNTIAGVPAATFQSINKRLASPVAPGALTTSGGSAPAQVVHTQPRAVGSPATA<br>ASDLVSLAPTQGVRAVTSVTASAVVTTSLTPVQTPTRSLVTQVSQATGVQLPGKTITPAHFQLLRQQQ<br>QQQQQQQQQQQQQQQAASQVQVPQIQGQAQSPAQIKAVGKLTPEHLIKMQKQKLQLPQQAPPPQ<br>APPGPPQPTAQVQVQPPQPTQQQSPQLTTVTAPRPGALLTGTTVANLQVARLTRVPTSQLQAQGQM<br>QAQAPQPAQVALAKPPVVSVPAAVVSSPGVTTLPMNVAGISVAIGQPQKTAGQTVVAQPVHVQQLL<br>KLKQQAVQQQKAIQPQAAPGPAAVQQKITAQQIAAQGQAQKVTYATQPALKTQFLTTPISQAQKL<br>AGTQQVQTQIQVAKLPQVVQQQTPVASIQQVASASQQASPQTVTLTQATAAGQQVQMIPAVTATA<br>QVVQQKLMQQQVVTTAAAQLQTPGVPNPAQAPASADSPNQQPKLQMRVPAVRLKTPTKPPCP	
14	E1A-binding protein p400 isoform X1	A0A480M084	No data	The same (13)	Match to KAAAAP at 1041<br>MPRYKLILGRMIAYLRQNDLDLEEEEEEEEEEEKSEVINDEQQALAGTLVAGAGSTVETDPFKRQQAG<br>PPAGGMPPAPQAAQLAGQKQSQQQYDPSTGPPVQNAASLHTPPPQLPGRLPAAGLPTVPLPPALQF<br>SQQPQMVEPQPQLQIPVKTQQPNAPVPAPPPSQLPAPPPQPTPPALHVPMPGKAQMQAPPLSAQPQ<br>TVASSRPPVDPAQPCPRPPPSSSTSSIAPASGSGPGPSPARASPVNRPSLATTKSLSPVTSRSPGAAVSALP<br>KPQSPAQNTAPPQDSSQDKLAEQIALENQIHQRIADLRKEGLWSLRRLPKLQEAPRPKSHWDYLLEE<br>MQWMATDFAQERRWKVAAAKKLVRTVARHHEEKKLREERGKKEEQNRLRRIAASTAREIEYFWSN<br>IEQVVEIKLQVELEEKRKKALHLQKVSRKGKELRSKGLDVLSENFLDSGTSGRKRRASTSLTDDEVEDE<br>EETIEEEEANEGAVDHQTELSNLAKEAELPLIDLMKLYEGAFLPNFQWPQPKPDSDETSEEEDMEDCS<br>GDRESRKDVVLIDSLFIMDQFKAAERVNVGKPHTKDIAEVTAVAEAVLPKGSARISTGVKLTAPALLY<br>GALRDYQKIGLDWLAKLYRKNLNGILADEAGLGKTVQIIAFFAHLACNEGNWGPHLVIVRSCNILK<br>WELELKRWCPGLKTLLYVGSHRELKAKRQEWSEPNSFNICITSYKQFFRGHASFPRARWKCLVIDEM<br>QRVKGMTERHWEAVFALQSQQRLLLIDAPLHNTFLELWTMVHFLIPGISRPYLHFPLKAPSEENQDY<br>YHKVVIRLHRVTQPFILRRTKRDVEKQLTKKYEHVLKCRLSNRQKALYEDVILQPGTQEALKSGHFVD<br>VLSILLRLQRICNHPGLVEPRLPESSYTAGPLQYRSASLILKALDRDFWKETDLSIFDLIGLENKMTHHE<br>AELLCKKKVTRKLFEELFTAPPLPSSGRPVPVRLKPSRLFQPVQYGQKPEGRTVAFPSAHPPRMAASAT<br>ATAAPQGHVRGRPPIATFSANPDAKAAAAPFQTSQASSGAPRHQPASTFSTAPSPAHPAKLRAPGPP<br>PPQPQAPSHPAGQSALPQGLVLTSQAQARLPSGEVVKIAQLASLAGPPSRVAQPETPVTLQFQGNKFT<br>LSHSQLRQLTAGQPLQLQGSVLQIVSAPGQSYLRPPGPVVMQTVSQAGALNALGSKPPAGGPGPTPV<br>TPPVAVPGRVAVSPLAAGEPGVASKPASPVAGPTQEEKTRLMKERLDQIYFVNERRCSRAPIYGRDLL<br>AVCSLLGREHMPWLRASESSTGKVTGPVSQFISPSKSQRDLILTLAQRQESLQDVINRVVCVIPPVVAA<br>PPRLWVARPPSLYSHKMRLFRHGLQEHTAPYAQQLQQMTALRSLQFPELRLVQFDSGKLEALAVLLQ<br>KLRSEGRRVLILSQMVLMLDILEMFLNFHYLTYIRIDENANSEQRQELMRSFNRDRRIFCALLSTHSRTT	

					GVSLVEADAVVFYDNDLNPVMDAKAQEWCDRIGRRRDVHIYRLVSGNSIEEKLLKNGTKDLIREVA<br>AQGNDYSMAFLTQRTIQELFEVYSPMDDAGFPVKAEEFVVLSQEPSVTETIAPKIARPFIEALKSIEYLEE<br>DAQKPFEEAVPEVSDSDSSRWNEEPSQLEELADFMGQLTPIEKYALNYLELFHTSIDQEKERNSEGAVL<br>SAVQEWEARNARMLREQEARELREQEQEQLLTYTREDAYNAEYVCEGAEGQTEVMPLWTPPTPPQ<br>DDNDIYIDSVMCLMYETTPIPESKLPPVYVRKERRRHKTDPSAAGRKKKQRHGEAVVPPRSLFDRATP<br>GMLKIRREGKEQKKNILLKQQTQFAKPLPTFAKPTAESGPDNPEWLISEDWALLQAVKQLLELPLNLT<br>IVSPAHTPNWDLVSDVVNSCSRIYRSSKQCRNRYENVLIPREEGKSKNNRPLRTGQMYAQDENATHT<br>QLYTSHFDLMKMTAGKRSPPIKPLLGMNPFQKNPKHASVLAESGINYDKPLPPIQVASLRAERIAKEK<br>KALADQQKAQQPPVAQPPPPPPQPQPPPPQQPPPPLPQPQATSSQQPTGPPAVQPQAQAQPPAQPT<br>QPPPKVPPAITTVGSAAVLQAGAIKTSVTGTSMPTGTVSGNVIVNTIAGVPAATFQSINKRLASPVAPG<br>ALTTSGGSAPAQVVHTQPRAVGSPATAASDLVSLAPTQGVRAVTSVTASAVVTTSLTPVQTPTRSLVT<br>QVSQATGVQLPGKTITPAHFQLLRQQQQQQQQQQQQQQQQQQQQAASQVQVPQIQGQAQSPAQIKA<br>VGKLTPEHLIKMQKQKLQLPQQAPPPQAPPGPPQPTAQVQVQPPQPTQQQSPQLTTVTAPRPGALLT<br>GTTVANLQVARLTRVPTSQLQAQGQMQAQAPQPAQVALAKPPVVSVPAAVVSSPGVTTLPMNVAG<br>ISVAIGQPQKTAGQTVVAQPVHVQQLLKLKQQAVQQQKAIQPQAAPGPAAVQQKITAQQIAAQGQ<br>AQKVTYATQPALKTQFLTTPISQAQKLAGTQQVQTQIQVAKLPQVVQQQTPVASIQQVASASQQASP<br>QTVTLTQATAAGQQVQMIPAVTATAQVVQQKLMQQQVVTTAAAQLQTPGVPNPAQAPASADSPN<br>QQPKLQMRVPAVRLKTPTKPPCP	
15	E1A-binding protein p400 isoform X1	A0A480K972	No data	The same (13)	Match to KAAAAP at 1081<br>MRKQCLDYHYREMEALKEAFKEYLIELFFLQHLQGNMMDFLAFKKKHYAPLQAYLRQNDLDLEEEE<br>EEEEEEEKSEVINDEQQALAGTLVAGAGSTVETDPFKRQQAGPPAGGMPPAPQAAQLAGQKQSQQQ<br>YDPSTGPPVQNAASLHTPPPQLPGRLPAAGLPTVPLPPALQFSQQPQMVEPQPQLQIPVKTQQPNAP<br>VPAPPPSQLPAPPPQPTPPALHVPMPGKAQMQAPPLSAQPQTVASSRPPVDPAQPCPRPPPSSSTSSIA<br>PASGSGPGPSPARASPVNRPSLATTKSLSPVTSRSPGAAVSALPKPQSPAQNTAPPQDSSQDKLAEQIA<br>LENQIHQRIADLRKEGLWSLRRLPKLQEAPRPKSHWDYLLEEMQWMATDFAQERRWKVAAAKKLV<br>RTVARHHEEKKLREERGKKEEQNRLRRIAASTAREIEYFWSNIEQVVEIKLQVELEEKRKKALHLQKV<br>SRKGKELRSKGLDVLSENFLDSGTSGRKRRASTSLTDDEVEDEEETIEEEEANEGAVDHQTELSNLAKE<br>AELPLIDLMKLYEGAFLPNFQWPQPKPDSDETSEEEDMEDCSGDRESRKDVVLIDSLFIMDQFKAAER<br>VNVGKPHTKDIAEVTAVAEAVLPKGSARISTGVKLTAPALLYGALRDYQKIGLDWLAKLYRKNLNGI<br>LADEAGLGKTVQIIAFFAHLACNEGNWGPHLVIVRSCNILKWELELKRWCPGLKTLLYVGSHRELKA<br>KRQEWSEPNSFNICITSYKQFFRGHASFPRARWKCLVIDEMQRVKGMTERHWEAVFALQSQQRLLLI<br>DAPLHNTFLELWTMVHFLIPGISRPYLHFPLKAPSEENQDYYHKVVIRLHRVTQPFILRRTKRDVEKQL<br>TKKYEHVLKCRLSNRQKALYEDVILQPGTQEALKSGHFVDVLSILLRLQRICNHPGLVEPRLPESSYTA<br>GPLQYRSASLILKALDRDFWKETDLSIFDLIGLENKMTHHEAELLCKKKVTRKLFEELFTAPPLPSSGRP<br>VPVRLKPSRLFQPVQYGQKPEGRTVAFPSAHPPRMAASATATAAPQGHVRGRPPIATFSANPDAKAA<br>AAPFQTSQASSGAPRHQPASTFSTAPSPAHPAKLRAPGPPPPQPQAPSHPAGQSALPQGLVLTSQAQ<br>ARLPSGEVVKIAQLASLAGPPSRVAQPETPVTLQFQGNKFTLSHSQLRQLTAGQPLQLQGSVLQIVSAP<br>GQSYLRPPGPVVMQTVSQAGALNALGSKPPAGGPGPTPVTPPVAVPGRVAVSPLAAGEPGVASKPAS<br>PVAGPTQEEKTRLMKERLDQIYFVNERRCSRAPIYGRDLLAVCSLMGREHMPWLRASESSTGKVTGP<br>VSQFISPSKSQRDLILTLTQRQESLQDVINRVVCVIPPVVAAPPRLWVARPPSLYSHKMRLFRHGLQEH<br>TAPYAQQLQQMTALRSLQFPELRLVQFDSGKLEALAVLLQKLRSEGRRVLILSQMVLMLDILEMFLNF<br>HYLTYIRIDENANSEQRQELMRSFNRDRRIFCALLSTHSRTTGVSLVEADAVVFYDNDLNPVMDAKA<br>QEWCDRIGRRRDVHIYRLVSGNSIEEKLLKNGTKDLIREVAAQGNDYSMAFLTQRTIQELFEVYSPMD<br>DAGFPVKAEEFVVLSQEPSVTETIAPKIARPFIEALKSIEYLEEDAQKPFEEAVPEVSDSDSSRWNEEPSQ<br>LEELADFMGQLTPIEKYALNYLELFHTSIDQEKERNSEGAVLSAVQEWEARNARMLREQEARELREQ<br>EQEQLLTYTREDAYNAEYVCEGAEGQTEVMPLWTPPTPPQDDNDIYIDSVMCLMYETTPIPESKLPPV<br>YVRKERRRHKTDPSAAGRKKKQRHGEAVVPPRSLFDRATPGMLKIRREGKEQKKNILLKQQTQFAKP<br>LPTFAKPTAESGPDNPEWLISEDWALLQAVKQLLELPLNLTIVSPAHTPNWDLVSDVVNSCSRIYRSSK<br>QCRNRYENVLIPREEGKSKNNRPLRTGQMYAQDENATHTQLYTSHFDLMKMTAGKRSPPIKPLLGM	90% with A0A480M084 (14)

					NPFQKNPKHASVLAESGINYDKPLPPIQVASLRAERIAKEKKALADQQKAQQPPVAQPPPPPPQPQPP<br>PPQQPPPPLPQPQATSSQQPTGPPAVQPQAQAQPPAQPTQPPPKAPPAITTVGSAAVLQAGAIKTSVT<br>GTSMPTGTVSGNVIVNTIAGVPAATFQSINKRLASPVAPGALTTSGGSAPAQVVHTQPRAVGSPATA<br>ASDLVSLAPTQGVRAVTSVTASAVVTTSLTPVQTPTRSLVTQVSQATGVQLPGKTITPAHFQLLRQQQ<br>QQQQQQQQQQQQQQQAASQVQVPQIQGQAQSPAQIKAVGKLTPEHLIKMQKQKLQLPQQAPPPQ<br>APPGPPQPTAQVQVQPPQPTQQQSPQLTTVTAPRPGALLTGTTVANLQVARLTRVPTSQLQAQGQM<br>QAQAPQPAQVALAKPPVVSVPAAVVSSPGTTLPMNVAGISVAIGQPQKTAGQTVVAQPVHVQQLLK<br>LKQQAVQQQKAIQPQAAPGPAAVQQKITAQQIAAQGQAQKVTYATQPALKTQFLTTPISQAQKLA<br>GTQQVQTQIQVAKLPQVVQQQTPVASIQQVASASQQASPQTVTLTQATAAGQQVQMIPAVTATAQ<br>VVQQKLMQQQVVTTAAAQLQTPGVPNPAQAPASADSPNQQPKLQMRVPAVRLKTPTKPPCP	
16	E1A-binding protein p400 isoform X1	A0A480K656	No data	The same (13)	Match to KAAAAP at 1132<br>MTSPPPPTSPSRTAVPPGLSSLPLTSAGSAGVRKAPRKLEEIPPASQELAQMRKQCLDYHYREMEALKE<br>AFKEYLIELFFLQHLQGNMMDFLAFKKKHYAPLQAYLRQNDLDLEEEEEEEEEEEKSEVINDEQQAL<br>AGTLVAGAGSTVETDPFKRQQAGPPAGGMPPAPQAAQLAGQKQSQQQYDPSTGPPVQNAASLHTP<br>PPQLPGRLPAAGLPTVPLPPALQFSQQPQMVEPQPQLQIPVKTQQPNAPVPAPPPSQLPAPPPQPTPP<br>ALHVPMPGKAQMQAPPLSAQPQTVASSRPPVDPAQPCPRPPPSSSTSSIAPASGSGPGPSPARASPVNR<br>PSLATTKSLSPVTSRSPGAAVSALPKPQSPAQNTAPPQDSSQDKLAEQIALENQIHQRIADLRKEGLWS<br>LRRLPKLQEAPRPKSHWDYLLEEMQWMATDFAQERRWKVAAAKKLVRTVARHHEEKKLREERGK<br>KEEQNRLRRIAASTAREIEYFWSNIEQVVEIKLQVELEEKRKKALHLQKVSRKGKELRSKGLDVLSENF<br>LDSGTSGRKRRASTSLTDDEVEDEEETIEEEEANEGAVDHQTELSNLAKEAELPLIDLMKLYEGAFLPN<br>FQWPQPKPDSDETSEEEDMEDCSGDRESRKDVVLIDSLFIMDQFKAAERVNVGKPHTKDIAEVTAVA<br>EAVLPKGSARISTGVKLTAPALLYGALRDYQKIGLDWLAKLYRKNLNGILADEAGLGKTVQIIAFFAH<br>LACNEGNWGPHLVIVRSCNILKWELELKRWCPGLKTLLYVGSHRELKAKRQEWSEPNSFNICITSYK<br>QFFRGHASFPRARWKCLVIDEMQRVKGMTERHWEAVFALQSQQRLLLIDAPLHNTFLELWTMVHFL<br>IPGISRPYLHFPLKAPSEENQDYYHKVVIRLHRVTQPFILRRTKRDVEKQLTKKYEHVLKCRLSNRQKA<br>LYEDVILQPGTQEALKSGHFVDVLSILLRLQRICNHPGLVEPRLPESSYTAGPLQYRSASLILKALDRDF<br>WKETDLSIFDLIGLENKMTHHEAELLCKKKVTRKLFEELFTAPPLPSSGRPVPVRLKPSRLFQPVQYGQ<br>KPEGRTVAFPSAHPPRMAASATATAAPQGHVRGRPPIATFSANPDAKAAAAPFQTSQASSGAPRHQ<br>PASTFSTAPSPAHPAKLRAPGPPPPQPQAPSHPAGQSALPQGLVLTSQAQARLPSGEVVKIAQLASLA<br>GPPSRVAQPETPVTLQFQGNKFTLSHSQLRQLTAGQPLQLQGSVLQIVSAPGQSYLRPPGPVVMQTVS<br>QAGALNALGSKPPAGGPGPTPVTPPVALPGRVAVSPLAAGEPGVASKPASPVAGPTQEEKTRLMKER<br>LDQIYFVNERRCSRAPIYGRDLLGVCSLMGREHMPWLRASESSTGKVTGPVSQFISPSKSQRDLILTLTQ<br>RQESLQDVINRVVCVIPPVVAAPPRLWVARPPSLYSHKMRLFRHGLQEHTAPYAQQLQQMTALRSL<br>QFPELRLVQFDSGKLEALAVLLQKLRSEGRRVLILSQMVLMLDILEMFLNFHYLTYIRIDENANSEQRQ<br>ELMRSFNRDRRIFCALLSTHSRTTGVSLVEADAVVFYDNDLNPVMDAKAQEWCDRIGRRRDVHIYRL<br>VSGNSIEEKLLKNGTKDLIREVAAQGNDYSMAFLTQRTIQELFEVYSPMDDAGFPVKAEEFVVLSQEP<br>SVTETIAPKIARPFIEALKSIEYLEEDAQKPFEEAVPEVSDSDSSRWNEEPSQLEELADFMGQLTPIEKYA<br>LNYLELFHTSIDQEKERNSEGAVLSAVQEWEARNARMLREQEARELREQEQEQLLTYTREDAYNAEY<br>VCEGAEGQTEVMPLWTPPTPPQDDNDIYIDSVMCLMYETTPIPESKLPPVYVRKERRRHKTDPSAAGR<br>KKKQRHGEAVVPPRSLFDRATPGMLKIRREGKEQKKNILLKQQTQFAKPLPTFAKPTAESGPDNPEW<br>LISEDWALLQAVKQLLELPLNLTIVSPAHTPNWDLVSDVVNSCSRIYRSSKQCRNRYENVLIPREEGKS<br>KNNRPLRTGQMYAQDENATHTQLYTSHFDLMKMTAGKRSPPIKPLLGMNPFQKNPKHASVLAESGI<br>NYDKPLPPIQVASLRAERIAKEKKALADQQKAQQPPVAQPPPPPPQPQPPPPQQPPPPLPQPQATSSQ<br>QPTGPPAVQPQAQAQPPAQPTQPPPKAPPAITTVGSAAVLQAGAIKTSVTGTSMPTGTVSGNVIVNTI<br>AGVPAATFQSINKRLASPVAPGALTTSGGSAPAQVVHTQPRAVGSPATAASDLVSLAPTQGVRAVTS<br>VTASAVVTTSLTPVQTPTRSLVTQVSQATGVQLPGKTITPAHFQLLRQQQQQQQQQQQQQQQQQQA<br>ASQVQVPQIQGQAQSPAQIKAVGKLTPEHLIKMQKQKLQLPQQAPPPQAPPGPPQPTAQVQVQPPQ<br>PTQQQSPQLTTVTAPRPGALLTGTTVANLQVARLTRVPTSQLQAQGQMQAQAPQPAQVALAKPPV<br>VSVPAAVVSSPGVTTLPMNVAGISVAIGQPQKTAGQTVVAQPVHVQQLLKLKQQAVQQQKAIQPQ	90% with A0A480M084 (14) and A0A480K972 (15)

					AAPGPAAVQQKITAQQIAAQGQAQKVTYATQPALKTQFLTTPISQAQKLAGTQQVQTQIQVAKLPQ<br>VVQQQTPVASIQQVASASQQASPQTVTLTQATAAGQQVQMIPAVTATAQVVQQKLMQQQVVTTA<br>AAQLQTPGVPNPAQAPASADSPNQQPKLQMRVPAVRLKTPTKPPCP	
17	E1A-binding protein p400 isoform X1	A0A480HPC9	No data	The same (13)	Match to KAAAAP at 1132<br>MTSPPPPTSPSRTAVPPGLSSLPLTSAGSAGVRKAPRKLEEIPPASQELAQMRKQCLDYHYREMEALKE<br>AFKEYLIELFFLQHLQGNMMDFLAFKKKHYAPLQAYLRQNDLDLEEEEEEEEEEEKSEVINDEQQAL<br>AGTLVAGAGSTVETDPFKRQQAGPPAGGMPPAPQAAQLAGQKQSQQQYDPSTGPPVQNAASLHTP<br>PPQLPGRLPAAGLPTVPLPPALQFSQQPQMVEPQPQLQIPVKTQQPNAPVPAPPPSQLPAPPPQPTPP<br>ALHVPMPGKAQMQAPPLSAQPQTVASSRPPVDPAQPCPRPPPSSSTSSIAPASGSGPGPSPARASPVNR<br>PSLATTKSLSPVTSRSPGAAVSALPKPQSPAQNTAPPQDSSQDKLAEQIALENQIHQRIADLRKEGLWS<br>LRRLPKLQEAPRPKSHWDYLLEEMQWMATDFAQERRWKVAAAKKLVRTVARHHEEKKLREERGK<br>KEEQNRLRRIAASTAREIEYFWSNIEQVVEIKLQVELEEKRKKALHLQKVSRKGKELRSKGLDVLSENF<br>LDSGTSGRKRRASTSLTDDEVEDEEETIEEEEANEGAVDHQTELSNLAKEAELPLIDLMKLYEGAFLPN<br>FQWPQPKPDSDETSEEEDMEDCSGDRESRKDVVLIDSLFIMDQFKAAERVNVGKPHTKDIAEVTAVA<br>EAVLPKGSARISTGVKLTAPALLYGALRDYQKIGLDWLAKLYRKNLNGILADEAGLGKTVQIIAFFAH<br>LACNEGNWGPHLVIVRSCNILKWELELKRWCPGLKTLLYVGSHRELKAKRQEWSEPNSFNICITSYK<br>QFFRGHASFPRARWKCLVIDEMQRVKGMTERHWEAVFALQSQQRLLLIDAPLHNTFLELWTMVHFL<br>IPGISRPYLHFPLKAPSEENQDYYHKVVIRLHRVTQPFILRRTKRDVEKQLTKKYEHVLKCRLSNRQKA<br>LYEDVILQPGTQEALKSGHFVDVLSILLRLQRICNHPGLVEPRLPESSYTAGPLQYRSASLILKALDRDF<br>WKETDLSIFDLIGLENKMTHHEAELLCKKKVTRKLFEELFTAPPLPSSGRPVPVRLKPSRLFQPVQYGQ<br>KPEGRTVAFPSAHPPRMAASATATAAPQGHVRGRPPIATFSANPDAKAAAAPFQTSQASSGAPRHQ<br>PASTFSTAPSPAHPAKLRAPGPPPPQPQAPSHPAGQSALPQGLVLTSQAQARLPSGEVVKIAQLASLA<br>GPPSRVAQPETPVTLQFQGNKFTLSHSQLRQLTAGQPLQLQGSVLQIVSAPGQSYLRPPGPVVMQTVS<br>QAGALNALGSKPPAGGPGPTPVTPPVAVPGRVAVSPLAAGEPGVASKPASPVAGPTQEEKTRLMKER<br>LDQIYFVNERRCSRAPIYGRDLLGVCSLMGREHMPWLRASESSTGKVTGPVSQFISPSKSQRDLILTLTQ<br>RQESLQDVINRVVCVIPPVVAAPPRLWVARPPSLYSHKMRLFRHGLQEHTAPYAQQLQQMTALRSL<br>QFPELRLVQFDSGKLEALAVLLQKLRSEGRRVLILSQMVLMLDILEMFLNFHYLTYIRIDENANSEQRQ<br>ELMRSFNRDRRIFCALLSTHSRTTGVSLVEADAVVFYDNDLNPVMDAKAQEWCDRIGRRRDVHIYRL<br>VSGNSIEEKLLKNGTKDLIREVAAQGNDYSMAFLTQRTIQELFEVYSPMDDAGFPVKAEEFVVLSQEP<br>SVTETIAPKIARPFIEALKSIEYLEEDAQKPFEEAVPEVSDSDSSRWNEEPSQLEELADFMGQLTPIEKYA<br>LNYLELFHTSIDQEKERNSEGAVLSAVQEWEARNARMLREQEARELREQEQEQLLTYTREDAYNAEY<br>VCEGAEGQTEVMPLWTPPTPPQDDNDIYIDSVMCLMYETTPIPESKLPPVYVRKERRRHKTDPSAAGR<br>KKKQRHGEAVVPPRSLFDRATPGMLKIRREGKEQKKNILLKQQTQFAKPLPTFAKPTAESGPDNPEW<br>LISEDWALLQAVKQLLELPLNLTIVSPAHTPNWDLVSDVVNSCSRIYRSSKQCRNRYENVLIPREEGKS<br>KNNRPLRTGQMYAQDENATHTQLYTSHFDLMKMTAGKRSPPIKPLLGMNPFQKNPKHASVLAESGI<br>NYDKPLPPIQVASLRAERIAKEKKALADQQKAQQPPVAQPPPPPPQPQPPPPQQPPPPLPQPQATSSQ<br>QPTGPPAVQPQAQAQPPAQPTQPPPKAPPAITTVGSAAVLQAGAIKTSVTGTSMPTGTVSGNVIVNTI<br>AGVPAATFQSINKRLASPVAPGALTTSGGSAPAQVVHTQPRAVGSPATAASDLVSLAPTQGVRAVTS<br>VTASAVVTTSLTPVQTPTRSLVTQVSQATGVQLPGKTITPAHFQLLRQQQQQQQQQQQQQQQQQA<br>ASQVQVPQIQGQAQSPAQIKAVGKLTPEHLIKMQKQKLQLPQQAPPPQAPPGPPQPTAQVQVQPPQ<br>PTQQQSPQLTTVTAPRPGALLTGTTVANLQVARLTRVPTSQLQAQGQMQAQAPQPAQVALAKPPV<br>VSVPAAVVSSPGVTTLPMNVAGISVAIGQPQKTAGQTVVAQPVHVQQLLKLKQQAVQQQKAIQPQ<br>AAPGPAAVQQKITAQQIAAQGQAQKVTYATQPALKTQFLTTPISQAQKLAGTQQVQTQIQVAKLPQ<br>VVQQQTPVASIQQVASASQQASPQTVTLTQATAAGQQVQMIPAVTATAQVVQQKLMQQQVVTTA<br>AAQLQTPGVPNPAQAPASADSPNQQPKLQMRVPAVRLKTPTKPPCP	90% with I3L7Y4 (12) and A0A287AHU7 (13)
18	5-hydroxytryptamine receptor 1A	A0A4X1UTF5	No data	GO:0004993<br>GO:0050795<br>GO:0046883	Match to KAAAAP at 270<br>MDVLSPDQGNNTTSSQGPFGARGNATGSSDVTFSYQVITSLLLGTLIFCAVLGNACVVAAIALERSLQ<br>NVANYLIGSLAVTDLMVSVLVLPMAALYQVLNKWTLGQVTCDLFIALDVLCCTSSILHLCAIALDRY	

				GO:0019229	WAITDPIDYVNKRTPRRAAALISLTWLIGFLISIPPMLGWRTPEDRSDPDACTISKDHGYTIYSTFGAFYI<br>PLLLMLVLYGRIFRAARFRIRKTVKKVEKKRGNNPLGASPAPQPPKSVNGEPGIRDWKQGMENKAA<br>AAPCANGAVRQGEEGAALEVIEVHRVGNSKEHLPLPNEASAVSCVPTSFKKKNERNAEAKRKMAL<br>ARERKTVKTLGIIMGTFILCWLPFFIVALVLPFCESSCHMPTLLGAIINWLGYSNSLLNPVIYAYFNKDF<br>QNAFKKIIKCKFCRR	
19	PEHE domain-containing protein	A0A4X1U9H6	Low in heart left ventricle	GO:0043984	Match to KAAAAP at 9<br>MTMRSAVFKAAAAPAGGNPEQRLDYERAAALGGPEDEPGAAEAHFLPRHRKLKEPGPPLASSQGGS<br>PAPSPAGCGGKGRGLLLPAGAAPGQQEESWGGSVPLPCPPPATKQAGIGGEPAAAGAGCSPRPKYQ<br>AVLPIQTGSLVAAAKEPTPWAGDKGGAAPPAATASDPAGPPPLPLPGPPPLAPTATAGTLAASEGRW<br>KSMRKSPLGGGGGSGASSQAACLKQILLLQLDLIEQQQQLQAKEKEIEELKSERDTLLARIERMERR<br>MQLVKKDNEKERHKLFQGYETEEREETELSEKIKLECQPELSETSQTLPPKPFSCGRSGKGHKRKSPFG<br>STERKIPVKKLAPEFSKVKTKTPKHSPVKEEPCGSLSETVCKRELRSQETPEKPRSSVDTPPRLSTPQKGP<br>STHPKEKAFSSEIEDLPYLSTTEMYLCRWHQPPPSPLPLRESSPKKEETVARCLMPSSVAGETSVLAVPS<br>WRDHSVEPLRDPNPSDLLENLDDSVFSKRHAKLELDEKRRKRWDIQRIREQRILQRLQLRMYKKKGI<br>QESEPEVTSFFPEPDDVESLMITPFLPVVAFGRPLPKLTPQNFELPWLDERSRCRLEIQKKQTPHRTCRK	
20	PEHE domain-containing protein	A0A4X1UEX9	Low in heart left ventricle	The same (19)	Match to KAAAAP at 9<br>MTMRSAVFKAAAAPAGGNPEQRLDYERAAALGGPEDEPGAAEAHFLPRHRKLKEPGPPLASSQGGS<br>PAPSPAGCGGKGRGLLLPAGAAPGQQEESWGGSVPLPCPPPATKQAGIGGEPAAAGAGCSPRPKYQ<br>AVLPIQTGSLVAAAKEPTPWAGDKGGAAPPAATASDPAGPPPLPLPGPPPLAPTATAGTLAASEGRW<br>KSMRKSPLGGGGGSGASSQAACLKQILLLQLDLIEQQQQLQAKEKEIEELKSERDTLLARIERMERR<br>MQLVKKDNEKERHKLFQGYETEEREETELSEKIKLECQPELSETSQTLPPKPFSCGRSGKGHKRKSPFG<br>STERKIPVKKLAPEFSKVKTKTPKHSPVKEEPCGSLSETVCKRELRSQETPEKPRSSVDTPPRLSTPQKGP<br>STHPKEKAFSSEIEDLPYLSTTEMYLCRWHQPPPSPLPLRESSPKKEETVAIPSWRDHSVEPLRDPNPSD<br>LLENLDDSVFSKRHAKLELDEKRRKRWDIQRIREQRILQRLQLRMYKKKGIQESEPEVTSFFPEPDDVE<br>SLMITPFLPVVAFGRPLPKLTPQNFELPWLDERSRCRLEIQKKQTPHRTCRK	90 % with A0A4X1U9H6 (19)
21	Death inducer-obliterator 1	A0A287AIZ5	Low in heart left ventricle	GO:0046872<br>GO:0097190<br>GO:0006351	Match to KAAAAP at 478<br>MDEKGDPSSEEAPKAIKPTSKEFRKTWGFRRTTIAKREGAGDAEPEALEQPPPQQGLSLRRSGRQPKR<br>TERVEEFLTTVRRRGRRSAPAPLEEPGEPASCPLTDGDTASEGSVDSASDGKGGPKPGSSAVKERPASS<br>KAKGGDDDEDDTSDSDSDGLTLKELQNRLRRKREQEPADRPPKGLQSRLRKKRREDPVETAGVEAA<br>DAGEGSLPVKAEPEADQGAASPAAEDDREGELQGPAAPGGREQERGGPGRPKPECEVYDPSALYCIC<br>RQPHNNRFMICCDRCEEWFHGDCVGISEARGRLLERNGEDYICPHCTILQVQDERSAESAGPQAAGP<br>RPADADGADLTSVGTVEQTSGGDQGIKGRIEKAANPSGKKKLKIFQPVVEAPGAAKCIGPGCSSAAQ<br>PDSVYCSSDCILKHAAATMRLLSAGKEPKPKPKEKGKTKPERLILQKCTVQAGIKVAPVHKRPAPDRK<br>ENAAKKAAAAPPRSESLAPEPASESSTPSWASDHNYNAVKPERTAAPRPPLLCKSGKEDRRVEKVVA<br>AAAPKKGAPPGSSGGGKQPAPRNLLPKKSPPFANAATARPAIRKSPSGFKGTVPKRPWLSAAPSGAG<br>PAAKQAGLAPGAASSAPKKFPGPAASAAAVKKPLPASAPLASPALGRLVPSSPASSQPNSQIRQNIRR<br>SLKEILWRRAADSDDLVMTESEVGRVALRIEKEMFGLFRVTDSRYKSKYRSLMFNLKDPKNQGLFHR<br>VLREDISLAKLVRMKPEELVSKELSVWKERPAKAVMEPRAKLHSESKKPAAKQETAPDLGDSPPVSDS<br>DEQQEAVRAAPERSAAPLDVFSSMLTDTTSQHRAHLFDLNCKICTGQVPSSEDEPAPKKQKVSASAR<br>KEDSKPVLGSPAPEPGLSSADDLVPDALPKHASEPDLGGAAQAHLEGTHPPVPAGDTPPAPATLEDP<br>PGPAPGGGSVLTTVTVSGRDPRTAPGAASTVTAPAAAQPPEPRPDPPKPVATSVTVPKSILAKPSTPPE<br>PRYLLSIPPSPSTSVPESRSPPDGDTSLFLSRLGTIWKGFINMQSVAKFVTKAYPVSGCFDHLSEDLPDTI<br>HVGGRIAPRTVWDYVGKLKSSVSKELCLIRFQPATEEEEVAYISLYSYFSSRGRFGVVANNSRHVKDLY<br>LIPLSARDPVPSKLLPFEGPGKHQPSEPTCPRPRTPGPAPCHQWALWGLASGMWAAGPRGSLPQARV<br>ASGKSQEAGAPRSAHPQAAPAPGEHPLPLRARGTWGERWAEARPPRGLLAGPSPACCSRGRLQPAS<br>SRALVPRRGLLQRTAYTLAQACRGPRVTPGGQLLLTPKKVT	

22	Death-inducer obliterator 1 isoform b	A0A480UG25	No data	The same (21)	Match to KAAAAP at 478<br>MDEKGDPSSEEAPKAIKPTSKEFRKTWGFRRTTIAKREGAGDAEPEALEQPPPQQGLSLRRSGRQPKR<br>TERVEEFLTTVRRRGRRSAPAPLEEPGEPASCPLTDGDTASEGSVDSASDGKGGPKPGSSAVKERPASS<br>KAKGGDDDEDDTSDSDSDGLTLKELQNRLRRKREQEPADRPPKGLQSRLRKKRREDPVETAGVEAA<br>DAGEGSLPVKAEPEADQGAASPAAEDDREGELQGPAAPGGREQERGGPGRPKPECEVYDPSALYCIC<br>RQPHNNRFMICCDRCEEWFHGDCVGISEARGRLLERNGEDYICPHCTILQVQDESSAESAGPQAAGP<br>RPADADGTDLTSVGTVEQTSGEDQGIKGRIEKAANPSGKKKLKIFQPVVEAPGAAKCIGPGCSSAAQP<br>DSVYCSSDCILKHAAATMRLLSAGKEPKPKPKEKGKTKPERLILQKCTVQAGIKVAPVHKRPAPDRKE<br>NAAKKAAAAPPRSEGLAPEPASESSTPSWASDHNYNAVKPERTAAPRPPLLCKSGKEDRRVEKVVA<br>AAAPKKGAPPGSSGGGKQPAPRNLLPKKSPPFANAATARPAIRKSPSGFKGTVPKRPWLSAAPSGAG<br>PAAKQAGLAPGAASSAPKKFPGPAASAAAVKKPLPASAPLASPALGRLVPSSPASSQPNSQIRQNIRR<br>SLKEILWRRAADSDDLVMTESEVGRVALRIEKEMFGLFRVTDSRYKSKYRSLMFNLKDPKNQGLFHR<br>VLREDISLAKLVRMKPEELVSKELSVWKERPAKAVMEPRAKLHSESKKPAAKQETAPDLGDSPPVSDS<br>DEQQEAVRAAPERSAAPLDVFSSMLTDTTSQHRAHLFDLNCKICTGQVPSSEDEPAPKKQKVSASAR<br>KEDSKPVLGSPAPEPGLSSADDLVPDALPKHASEPDLGGAAQAHLEGTHPPVPAGDTPPAPATLEDP<br>PGPAPGGGSVLTTVTVSGRDPRTAPGAASTVTAPAAAQPPEPRPDPPKPVATSVTVPKSILAKPSTPPE<br>PRYLLSIPPSPSTSVPESRSPPDGDTSLFLSRLGTIWKGFINMQSVAKFVTKAYPVSGCFDHLSEDLPDTI<br>HVGGRIAPRTVWDYVGKLKSSVSKELCLIRFQPATEEEEVAYISLYSYFSSRGRFGVVANNSRHVKDLY<br>LIPLSARDPVPSKLLPFEGPGKHQPSEPTCPRPRTPGPAPCHQWACRLGHRP	90 % with A0A287AIZ5 (21)
23	Death inducer-obliterator 1	A0A287AIF6	Low in heart left ventricle	The same (21)	Match to KAAAAP at 478<br>MDEKGDPSSEEAPKAIKPTSKEFRKTWGFRRTTIAKREGAGDAEPEALEQPPPQQGLSLRRSGRQPKR<br>TERVEEFLTTVRRRGRRSAPAPLEEPGEPASCPLTDGDTASEGSVDSASDGKGGPKPGSSAVKERPASS<br>KAKGGDDDEDDTSDSDSDGLTLKELQNRLRRKREQEPADRPPKGLQSRLRKKRREDPVETAGVEAA<br>DAGEGSLPVKAEPEADQGAASPAAEDDREGELQGPAAPGGREQERGGPGRPKPECEVYDPSALYCIC<br>RQPHNNRFMICCDRCEEWFHGDCVGISEARGRLLERNGEDYICPHCTILQVQDERSAESAGPQAAGP<br>RPADADGADLTSVGTVEQTSGGDQGIKGRIEKAANPSGKKKLKIFQPVVEAPGAAKCIGPGCSSAAQ<br>PDSVYCSSDCILKHAAATMRLLSAGKEPKPKPKEKGKTKPERLILQKCTVQAGIKVAPVHKRPAPDRK<br>ENAAKKAAAAPPRSESLAPEPASESSTPSWASDHNYNAVKPERTAAPRPPLLCKSGKEDRRVEKVVA<br>AAAPKKGAPPGSSGGGKQPAPRNLLPKKSPPFANAATARPAIRKSPSGFKGTVPKRPWLSAAPSGAG<br>PAAKQAGLAPGAASSAPKKFPGPAASAAAVKKPLPASAPLASPALGRLVPSSPASSQPNSQIRQNIRR<br>SLKEILWRRAADSDDLVMTESEVGRVALRIEKEMFGLFRVTDSRYKSKYRSLMFNLKDPKNQGLFHR<br>VLREDISLAKLVRMKPEELVSKELSVWKERPAKAVMEPRAKLHSESKKPAAKQETAPDLGDSPPVSDS<br>DEQQEAVRAAPERSAAPLDVFSSMLTDTTSQHRAHLFDLNCKICTGQVPSSEDEPAPKKQKVSASAR<br>KEDSKPVLGSPAPEPGLSSADDLVPDALPKHASEPDLGGAAQAHLEGTHPPVPAGDTPPAPATLEDP<br>PGPAPGGGSVLTTVTVSGRDPRTAPGAASTVTAPAAAQPPEPRPDPPKPVATSVTVPKSILAKPSTPPE<br>PRYLLSIPPSPSTSVPESRSPPDGDTSLFLSRLGTIWKGFINMQSVAKFVTKAYPVSGCFDHLSEDLPDTI<br>HVGGRIAPRTVWDYVGKLKSSVSKELCLIRFQPATEEEEVAYISLYSYFSSRGRFGVVANNSRHVKDLY<br>LIPLSARDPVPSKLLPFEGPGLESPRPNIILGLVICQKVKRPSGAGELDKPEEKRARPPTQDELDGAPYA<br>KAPGALLPEKKAPKYPLSSGDLAASSTPPGSPPPPPPPPEAPAAPASASVLKILSALKPGAASAVSPASS<br>AVAAAAAPAAPSASKTASPLEHILQTLFGKKKSFDPPTKEAVEAAPACLDPHARADSGLPAAPLLDPI<br>VQQFGQFSKDRALEEEEDDRPYDPEEEYGPERALDAPLLERGRRQDAEAAPEAAEREEVAYDPEDETI<br>LEEAKVTIDDLPNRMCADAQAGPGAPTPSLAEQQQMIEELSRQIEEQKRQVEEQEEALRLQRAAVGA<br>SMAHFSVSDALMSPPPKAELFPQEPQAGGRAAVLPASSQGPGPSADLRPSRDPRQARRLAAESSESEA<br>APKPPANGTRGAPPAREVPGGAVAGPGPVPALEEPDSAPPPWAPDEEAALPAKQDGPLGERSQSPG<br>QQPGESAQPMAPGDGVPRPARKVLLPTPPGASFQPHFPSQNEGQNFNPPGGRDAFSGPLYASQEKAL<br>SCFQYEDPRPAQFAGRGDPPTAEAEGDREPQPRPGEGPGLFPPATQKGGPPPQFPGQREPAPPRTFG<br>MSGLHGPNFPGPRGPVPPCSEENVVSNNEGPRGGAPPGRFGPQKGPIPSLFSGPHGPPPYGDGRGPSP<br>SHLGGPRGGAPPPFEDRTDPHGEKREFQDGAYSEAVGPPAPFEGPEQGQFLGGRGGGAPFQFGGQR	

					RPLLSQFKGPRGGPPPSQFGGQRGPPPGHFGGPRGPHPGQFEGPRGQAPNFMPGPRGIQPQPFEEQR<br>VHSPPRFASQRAPAPLQFAGPRGSAPFPEQGEAAPPRFQFQGQAAPGSKPAPRPLLELPSHPPQHRKD<br>RWDDAGPAPALPAAPGPGPEADFREAKGHEYRNQAFEGRPRERFEAGPKEKPPDEPEAPRPTAGRA<br>APSMTGAASGSVGSPGSGSAAAPGAASGTGTGAASGTDTATRTLAVSGTAAASGTRAGTRGASGTG<br>AASGAATGTGTAGVTARGPAAGTATATRPASGSEAGTAGTAAGARSGPGTPRPRPRGPPTPPPRPRA<br>PAQSRTLWIQK	
24	MSL complex subunit 1	F1RXC8	Medium-low in heart left ventricle (67.87) Medium in endocardial endothelium (83.48) and heart (79.78)	GO:0003682<br>GO:0043984	Match to KAAAAP at 9<br>MTMRSAVFKAAAAPAGGNPEQRLDYERAAALGGPEDEPGAAEAHFLPRHRKLKEPGPPLASSQGGS<br>PAPSPAGCGGKGRGLLLPAGAAPGQQEESWGGSVPLPCPPPATKQAGIGGEPAAAGAGCSPRPKYQ<br>AVLPIQTGSLVAAAKEPTPWAGDKGGAAPPAATASDPAGPPPLPLPGPPPLAPTATAGTLAASEGRW<br>KSMRKSPLGGGGGSGASSQAACLKQILLLQLDLIEQQQQLQAKEKEIEELKSERDTLLARIERMERR<br>MQLVKKDNEKERHKLFQGYETEEREETELSEKIKLECQPELSETSQTLPPKPFSCGRSGKGHKRKSPFG<br>STERKIPVKKLAPEFSKVKTKTPKHSPVKEEPCGSLSETVCKRELRSQETPEKPRSSVDTPPRLSTPQKGP<br>STHPKEKAFSSEIEDLPYLSTTEMYLCRWHQPPPSPLPLRESSPKKEETVARCLMPSSVAGETSVLAVPS<br>WRDHSVEPLRDPNPSDLLENLDDSVFSKRHAKLELDEKRRKRWDIQRIREQRILQRLQLRMYKKKGI<br>QESEPEVTSFFPEPDDVESLMITPFLPVVAFGRPLPKLTPQNFELPWLDERSRCRLEIQKKQTPHRTCRK	100 % with A0A4X1U9H6 (19) 90% with A0A4X1U9H6 (19) and A0A4X1UEX9 (20)
25	MSL complex subunit 1	A0A286ZQ98	Low in heart left ventricle	GO:0043984	Match to KAAAAP at 9<br>MTMRSAVFKAAAAPAGGNPEQRLDYERAAALGGPEDEPGAAEAHFLPRHRKLKEPGPPLASSQGGS<br>PAPSPAGCGGKGRGLLLPAGAAPGQQEESWGGSVPLPCPPPATKQAGIGGEPAAAGAGCSPRPKYQ<br>AVLPIQTGSLVAAAKEPTPWAGDKGGAAPPAATASDPAGPPPLPLPGPPPLAPTATAGTLAASEGRW<br>KSMRKSPLGGGGGSGASSQAACLKQILLLQLDLIEQQQQLQAKEKEIEELKSERDTLLARIERMERR<br>MQLVKKDNEKERHKLFQGYETEEREETELSEKIKLECQPELSETSQTLPPKPFSCGRSGKGHKRKSPFG<br>STERKIPVKKLAPEFSKVKTKTPKHSPVKEEPCGSLSETVCKRELRSQETPEKPRSSVDTPPRLSTPQKGP<br>STHPKEKAFSSEIEDLPYLSTTEMYLCRWHQPPPSPLPLRESSPKKEETVAIPSWRDHSVEPLRDPNPSD<br>LLENLDDSVFSKRHAKLELDEKRRKRWDIQRIREQRILQRLQLRMYKKKGIQESEPEVTSFFPEPDDVE<br>SLMITPFLPVVAFGRPLPKLTPQNFELPWLDERSRCRLEIQKKQTPHRTCRK	100 % with A0A4X1UEX9 (20) 90% with A0A4X1U9H6 (19) and F1RXC8 (24) A0A4X1UEX9 (20)
26	MSL complex subunit 1	A0A5G2QBE7	No data	The same (25)	Match to KAAAAP at 9<br>MTMRSAVFKAAAAPAGGNPEQRLDYERAAALGGPEDEPGAAEAHFLPRHRKLKEPGPPLASSQGGS<br>PAPSPAGCGGKGRGLLLPAGAAPGQQEESWGGSVPLPCPPPATKQAGIGGEPAAAGAGCSPRPKYQ<br>AVLPIQTGSLVAAAKEPTPWAGDKGGAAPPAATASDPAGPPPLPLPGPPPLAPTATAGTLAASEGRW<br>KSMRKSPLGGGGGSGASSQAACLKQILLLQLDLIEQQQQLQAKEKEIEELKSERDTLLARIERMERR<br>MQLVKKDNEKERHKLFQGYETEEREETELSEKIKLECQPELSETSQTLPPKPFSCGRSGKGHKRKSPFG<br>STERKIPVKKLAPEFSKVKTKTPKHSPVKEEPCGSLSETVCKRELRSQETPEKPRSSVDTPPRLSTPQKGP<br>STHPKEKAFSSEIEDLPYLSTTEMYLCRWHQPPPSPLPLRESSPKKEETVARCLMPSSVAGETSVLAVPS<br>WRDHSVEPLRDPNPSDLLENLDDSVFSKRHAKLELDEKRRKRWDIQRIREQRILQRLQLRMYKKKGI<br>QESEPEVTSFFPEPDDVESLMITPFLPVVAFGRPLPKLTPQNFELPWLDERSRCRLEIQKKQTPHRTYQL<br>SFDLDMLKFC	90% with A0A4X1U9H6 (19), F1RXC8 (24), A0A286ZQ98 (25) and A0A4X1UEX9 (20)
27	Nucleolar and coiled-body	A0A480HQA8	No data	GO:0005515	Match to KAAAAP at 261<br>MADAGLRRVVPSDLYPLVLGFLRDNQLSDVANKFAKATGVTQQDANASSLLDIYSFWLNRSTKAPK<br>RKLQANGPVTKKAKKKTSSSDSSEDSSEEEKAQGPPAKKAAAPAKRAGLPQQPGKATAKASESSSSS<br>SEESSDEEEDKKKKPVQKVVKPQGKAVKAPPKKAESSDSDSDSSSEDEAPKNQKPKTAAVAAKAQAK	90% with A0A4X1TTP7 (1)

	phosphoprotein 1 isoform 3				VPAKTGTPARAAPKVANGKAASSSSSSSSSSDDDSEEEKAAAVSKKTVPKKQVVVKAPVKAAAAPAQ<br>KSSSSEDSSSEEEEEEQKKKPMKKKPGTYSSVPPPSVPSPKKPLGTQAPKKAAGKQEPVESSEDSSDESD<br>SSSEEEKKPPAKAVVPKAATKAAPAKKAAESSSDSSDSDSSEDEAPAKPASATKNSSKPAATPKQSIAK<br>PATSSKQPVGSGQKPLTRKADSSSSEEESSSSEDEKMKKTVATPKSKVTAKAAPSLPAKQASQGGGDSS<br>SDSDSSSSEEEEEEKTSKTPAKKMPQKDMGAVAPSKPASAKQAKAESSSSSSSDDSSEEEEEDKPKGKST<br>PKPQAPKANGTTALTAQNGKADRDSNEEEEDKKKAAVGVSKPGSGKKRKQNEAAKETETPPAKKIK<br>PQTPNTFPKRKKGERRGSSPFRRIREEEIEVDARVADNSFDAKRGAAGDWGERANQVLKFTKGKSFR<br>HEKTKKKRGSYRGGSISVQVNSIKFDSE	
28	Nucleolar and coiled-body phosphoprotein 1 isoform 3	A0A480HP04	No data	The same (27)	Match to KAAAAP at 261<br>MADAGLRRVVPSDLYPLVLGFLRDNQLSDVANKFAKATGVTQQDANASSLLDIYSFWLNRSTKAPK<br>RKLQANGPVTKKAKKKTSSSDSSEDSSEEEKAQGPPAKKAAAPAKRAGLPQQPGKATAKASESSSSSS<br>SEESSDEEEDKKKKPVQKVVKPQGKAVKAPPKKAESSDSDSDSSSEDEAPKNQKPKTAAVAAKAQAK<br>VPAKTGTPARAAPKVANGKAASSSSSSSSSSDDDSEEEKAAAVSKKTVPKKQVVVKAPVKAAAAPAQ<br>KSSSSEDSSSEEEEEEQKKKPMKKKPGTYSSVPPPSVPSPKKPLGTQAPKKAAGKQEPVESSEDSSDESD<br>SSSEEEKKPPAKAVVPKAATKAAPAKKAAESSSDSSDSDSSEDEAPAKPASATKNSSKPAATPKQSIAK<br>PATSSKQPVGSGQKPLTRKADSSSSEEESSSSEDEKMKKTVATPKSKVTAKAAPSLPAKQASQGGGDSS<br>SDSDSSSSEEEEEEKTSKTPAKKMPQKDVGAVAPSKPASAKQAKAESSSSSSSDDSSEEEEEDKPKGKST<br>PKPQAPKANGTTALTAQNGKADRDSNEEEEDKKKAAVGVSKPGSGKKRKQNEAAKETETPPAKKIK<br>PQTPNTFPKRKKGERRGSSPFRRIREEEIEVDARVADNSFDAKRGAAGDWGERANQVLKFTKGKSFR<br>HEKTKKKRGSYRGGSISVQVNSIKFDSE	90% with A0A4X1TTP7 (1) and A0A480HQA8 (27)
29	Nucleolar and coiled-body phosphoprotein 1	F1S8T1	Medium in heart (81.79) and endocardial endothelium (76.26); low in heart left ventricle (65.79)	GO:0046982<br>GO:0030674<br>GO:0014032<br>GO:0014029<br>GO:0007000<br>GO:0006417	Match to KAAAAP at 261<br>MADAGLRRVVPSDLYPLVLGFLRDNQLSDVANKFAKATGVTQQDANASSLLDIYSFWLNRSTKAPK<br>RKLQANGPVTKKAKKKTSSSDSSEDSSEEEKAQGPPAKKAAAPAKRAGLPQQPGKATAKASESSSSSS<br>SEESSDEEEDKKKKPVQKVVKPQGKAVKAPPKKAESSDSDSDSSSEDEAPKNQKPKTAAVAAKAQAK<br>VPAKTGTPARAAPKVANGKAASSSSSSSSSSDDDSEEEKAAAVSKKTVPKKQVVVKAPVKAAAAPAQ<br>KSSSSEDSSSEEEEEEQKKKPMKKKPGTYSSVPPPSVPSPKKPLGTQAPKKAAGKQEPVESSEDSSDESD<br>SSSEEEKKPPAKAVVPKAATKAAPAKKAAESSSDSSDSDSSEDEAPAKPASATKNSTKPATSSKQPVGS<br>GQKPLTRKADSSSSEEESSSSEDEKMKKTVATPKSKVTAKAAPSLPAKQASQGGGDSSSDSDSSSSEEEE<br>EEKTSKTPAKKMPQKDVGAVAPSKPASAKQAKAESSSSSSSDDSSEEEEEDKPKGKSTPKPQAPKANG<br>TTALTAQNGKADRDSNEEEEDKKKAAVGVSKPGSGKKRKQNEAAKETETPPAKKIKPQTPNTFPKR<br>KKGERRGSSPFRRIREEEIEVDARVADNSFDAKRGAAGDWGERANQVLKFTKGKSFRHEKTKKKRGS<br>YRGGSISVQVNSIKFDSE	90% with A0A4X1TTP7 (1), A0A480HQA8 (27) and A0A480HP04 (28)
30	CCAAT enhancer binding protein alpha	F1RNW6	Low in heart (49.59) and endocardial endothelium (47.43)	GO:0003682<br>GO:0001228<br>GO:0000981<br>GO:0042802<br>GO:0019900<br>GO:0000978<br>GO:0008134<br>GO:0050873<br>GO:0048469<br>GO:0071407<br>GO:0071356<br>GO:0008203	Match to KAAAAP at 92<br>MESADFYEAEPRPPMSSHLQSPPHAPSSAAFGFPRGAGPAQPPAPPAAPEPLGGICEHETSIDISAYIDP<br>AAFNDEFLADLFQHSRQQEKAKAAAAPAGGGGDFDYPGAPVGPGGAVMPGGAHGPPPSYGCAAA<br>GYLDGRLEPLYERVGAPALRPLVIKQEPREEDESKQLALAGLFPYQPPPPPPPPHSHPPPAHLAAPHL<br>QFQIAHCGQTTMHLQPGHPTPPPTPVPSPHPAPALGAAGLPGPGGALKGLAPAHPDLRAGGGGGA<br>GKAKKSVDKNSNEYRVRRERNNIAVRKSRDKAKQRNVETQQKVLELTSDNDRLRKRVEQLSRELDT<br>LRGIFRQLPESSLVKAMGNCA	

				GO:0001892<br>GO:0042593<br>GO:0030851<br>GO:0048839<br>GO:0070102<br>GO:0055088<br>GO:0001889<br>GO:0030324<br>GO:0030225<br>GO:0007005<br>GO:0030099<br>GO:0045786<br>GO:0008285<br>GO:0000122<br>GO:0007219<br>GO:0045600<br>GO:0050729<br>GO:0043032<br>GO:0045669<br>GO:0045945<br>GO:0006357<br>GO:0000050<br>GO:0050872		
31	BZIP domain-containing protein	A0A4X1TNP2	The same (30)	The same (30)	Match to KAAAAP at 92<br>MESADFYEAEPRPPMSSHLQSPPHAPSSAAFGFPRGAGPAQPPAPPAAPEPLGGICEHETSIDISAYIDP<br>AAFNDEFLADLFQHSRQQEKAKAAAAPAGGGGDFDYPGAPVGPGGAVMPGGAHGPPPSYGCAAA<br>GYLDGRLEPLYERVGAPALRPLVIKQEPREEDESKQLALAGLFPYQPPPPPPPPHSHPPPAHLAAPHL<br>QFQIAHCGQTTMHLQPGHPTPPPTPVPSPHPAPALGAAGLPGPGGALKGLAPAHPDLRAGGGGGA<br>GKAKKSVDKNSNEYRVRRERNNIAVRKSRDKAKQRNVETQQKVLELTSDNDRLRKRVEQLSRELDT<br>LRGIFRQLPESSLVKAMGNCA	100%with F1RNW6 (30)
32	Uncharacterized protein	A0A4X1SIV7	No data	The same (21)	Match to KAAAAP at 551<br>MFAATHGGLSRPARPGTPFSGAGWRIVPLCPVGGPSHPSRAASVPACPTAGRSGPSARPRWTPVMLC<br>LRFLSGMDEKGDPSSEEAPKAIKPTSKEFRKTWGFRRTTIAKREGAGDAEPEALEQPPPQQGLSLRRSG<br>RQPKRTERVEEFLTTVRRRGRRSAPAPLEEPGEPASCPLTDGDTASEGSVDSASDGKGGPKPGSSAVKE<br>RPASSKAKGGDDDEDDTSDSDSDGLTLKELQNRLRRKREQEPADRPPKGLQSRLRKKRREDPVETAG<br>VEAADAGEGSLPVKAEPEADQGAASPAAEDDREGELQGPAAPGGREQERGGPGRPKPECEVYDPSA<br>LYCICRQPHNNRFMICCDRCEEWFHGDCVGISEARGRLLERNGEDYICPHCTILQVQDERSAESAGPQ<br>AAGPRPADADGADLTSVGTVEQTSGGDQGIKGRIEKAANPSGKKKLKIFQPVVEAPGAAKCIGPGCS<br>SAAQPDSVYCSSDCILKHAAATMRLLSAGKEPKPKPKEKGKTKPERLILQKCTVQAGIKVAPVHKRP<br>APDRKENAAKKAAAAPPRSESLAPEPASESSTPSWASDHNYNAVKPERTAAPRPPLLCKSGKEDRRV<br>EKVVAAAAPKKGAPPGSSGGGKQPAPRNLLPKKSPPFANAATARPAIRKSPSGFKGTVPKRPWLSAA<br>PSGAGPAAKQAGLAPGAASSAPKKFPGPAASAAAVKKPLPASAPLASPALGRLVPSSPASSQPNSQIR	

					QNIRRSLKEILWRRAADSDDLVMTESEVGRVALRIEKEMFGLFRVTDSRYKSKYRSLMFNLKDPKNQG<br>LFHRVLREDISLAKLVRMKPEELVSKELSVWKERPAKAVMEPRAKLHSESKKPAAKQETAPDLGDSPP<br>VSDSDEQQEAVRAAPERSAAPLDVFSSMLTDTTSQHRAHLFDLNCKICTGQVPSSEDEPAPKKQKVS<br>ASARKEDSKPVLGSPAPEPGLSSADDLVPDALPKHASEPDLGGAAQAHLEGTHPPVPAGDTPPAPAT<br>LEDPPGPAPGGGSVLTTVTVSGRDPRTAPGAASTVTAPAAAQPPEPRPDPPKPVATSVTVPKSILAKPS<br>TPPSPDTSCPSLRLRAPASRSRGRLPMETPPSSCLGSAPSGKGSLTCRASRNLSRKRIPSLGVLTTSVRTCP<br>TRSTSAGGSPPGRSGITWASSSLPCPRSCV	
33	Uncharacterized protein	A0A4X1SJ53	No data	The same (21)	Match to KAAAAP at 478<br>MDEKGDPSSEEAPKAIKPTSKEFRKTWGFRRTTIAKREGAGDAEPEALEQPPPQQGLSLRRSGRQPKR<br>TERVEEFLTTVRRRGRRSAPAPLEEPGEPASCPLTDGDTASEGSVDSASDGKGGPKPGSSAVKERPASS<br>KAKGGDDDEDDTSDSDSDGLTLKELQNRLRRKREQEPADRPPKGLQSRLRKKRREDPVETAGVEAA<br>DAGEGSLPVKAEPEADQGAASPAAEDDREGELQGPAAPGGREQERGGPGRPKPECEVYDPSALYCIC<br>RQPHNNRFMICCDRCEEWFHGDCVGISEARGRLLERNGEDYICPHCTILQVQDERSAESAGPQAAGP<br>RPADADGADLTSVGTVEQTSGGDQGIKGRIEKAANPSGKKKLKIFQPVVEAPGAAKCIGPGCSSAAQ<br>PDSVYCSSDCILKHAAATMRLLSAGKEPKPKPKEKGKTKPERLILQKCTVQAGIKVAPVHKRPAPDRK<br>ENAAKKAAAAPPRSESLAPEPASESSTPSWASDHNYNAVKPERTAAPRPPLLCKSGKEDRRVEKVVA<br>AAAPKKGAPPGSSGGGKQPAPRNLLPKKSPPFANAATARPAIRKSPSGFKGTVPKRPWLSAAPSGAG<br>PAAKQAGLAPGAASSAPKKFPGPAASAAAVKKPLPASAPLASPALGRLVPSSPASSQPNSQIRQNIRR<br>SLKEILWRRAADSDDLVMTESEVGRVALRIEKEMFGLFRVTDSRYKSKYRSLMFNLKDPKNQGLFHR<br>VLREDISLAKLVRMKPEELVSKELSVWKERPAKAVMEPRAKLHSESKKPAAKQETAPDLGDSPPVSDS<br>DEQQEAVRAAPERSAAPLDVFSSMLTDTTSQHRAHLFDLNCKICTGQVPSSEDEPAPKKQKVSASAR<br>KEDSKPVLGSPAPEPGLSSADDLVPDALPKHASEPDLGGAAQAHLEGTHPPVPAGDTPPAPATLEDP<br>PGPAPGGGSVLTTVTVSGRDPRTAPGAASTVTAPAAAQPPEPRPDPPKPVATSVTVPKSILAKPSTPPS<br>PDTSCPSLRLRAPGEASTRPDEPPRVLFLGDGKGQHERVRGPAPRAAREDGFPQFRVSGRRLKASVLG<br>RLTSTLETDLENCSLCP	90% with A0A287AIZ5 (21) and A0A480UG25 (22)
**NLHLP**						
1	Steroid 21-hydroxylase	P15540	Low in endocardial endothelium (41.74)	GO:0103069<br>GO:0020037<br>GO:0005506<br>GO:0106309<br>GO:0004509<br>GO:0005496<br>GO:0008395<br>GO:0006704<br>GO:0006694<br>GO:0008202	Match to NLHLP at 27<br>MVLVWLLLLLTLLAGARLLWGQWKLRNLHLPPLVPGFLHLLQPNLPIYLLGLTQRLGPIYRLRLGLQ<br>DVVVLNSKRTIEEALVRKWVDFAGRPQIPSYKLASQHCPDISLGDYSLFWKAHKKLTRSALLLGVRSS<br>MEPRVEQLTQEFCERMRAQAGTPVTIQKEFSVLTCSIICCLTFGDKEDTLVHALHDCVQDLMKTWEH<br>WSIQILDMVPFLRFFPSPGLRRLKQAIENRDHLVEKQLRRHKESMVAGQWRDMLDYMLQEAGRQRV<br>EEGQGQLLEGHVHMSVVDLFIGGTETTANTLSWAVVYLLHHPEIQWRLQEELDRELGPGAAGSRVP<br>YKDRARLPLLNATIAEVLRLRPVVPLALPHRATRPSSIFGYDIPEGTVVIPNLQGAHLDETVWEQPHEF<br>RPDRFLAPGANPSALAFGCGARVCLGEPLARLELFVVLVQLLQAFTLLPPEGALPSLQPHPHSGINLK<br>VQPFQVRLQPRGGRGEGPGPR	
2	Alpha-1-antitrypsin	P50447	No data	GO:0004867<br>GO:0010951	Match to NLHLP at 312<br>MASSSTWGLLLLAGLCCLVPISLAEGLQGHAVQETDVPRHDHEQHQEAACHRIAPNLADFAFSLYR<br>QVARQSNTSNIFLSPVTIARAFAMLSLGTKGATHAEILEGLQFNLTEKAEAEIHEGFQHLLHTLNQPD<br>NQLQLTTGNGLFIDEKAKLVPKFLEDVKNLYHSEAFSINFRDTEEAKKCINDYVEKGSQGKIVDLVDE	

					LDKDTVFALVNYIFFKGKWEKPFEVEQTTEEDFHVDEETTVKVPMMNRLGMFDLHHCDKLSSWVLL<br>MDYVATATAFFILPDQGKLHQLEDMLTKEIRAKFLEKRYPSSANLHLPKLTISGTYDLKSLLGNLGITK<br>VFSDEADLSGVTEEQPLKLSKALHRAVLTIDEKGTEATGATILEAIPMSIPPNVKFNKPFLFLIYDTKTK<br>AVLFMGKVMNPTQK	
3	Alpha-1-antitrypsin	F1SCF0	Low in heart (45.04)	GO:0042802<br>GO:0002020<br>GO:0004867<br>GO:0010951	Match to NLHLP at 311<br>MASSSTWGLLLLAGLCCLVPISLAEGLQGHAVQETDVPRHDHEQHQEAACHRIAPNLADFAFSLYR<br>QVARQSNTSNIFLSPVSIATAFAMLSLGTKGATHAEILEGLQFNLTEKAEAEIHEGFQHLLHTLNQPD<br>NQLQLTTGNGLFVDKKAKLVPKFLEDVKNLYHSEAFSINFRDTEEAKKCINDYVEKGSQGKIVDLVD<br>ELDKDTVFALVNYIFFKGKWEKPFEVEQTTEEDFHVDEETTVKVPMMNRLGMFDLHHCEKLSSWVL<br>LMDYVGNATAFFILPDGKLHQLEDMLTKEILAKFLEKRYPSSANLHLPKLTISGTYDLKSLLGNLGITK<br>VFSDEADLSGVTEEQPLKLSKALHRAVLTIDEKGTEATGATILEAIPMSIPPNVKFNKPFLFLIYDTKTK<br>AVLFMGKVMNPTQK	90% with P50447 (2)
4	Alpha-1-antitrypsin	A0A4X1SHV8	No data	No data	Match to NLHLP at 312<br>MASSSTWGLLLLAGLCCLVPISLAEGLQGHAVQETDVPRHDHEQHQEAACHRIAPNLADFAFSLYR<br>QVARQSNTSNIFLSPVSIATAFAMLSLGTKGATHAEILEGLQFNLTEKAEAEIHEGFQHLLHTLNQPD<br>NQLQLTTGNGLFIDEKAKLVPKFLEDVKNLYHSEAFSINFRDTEEAKKCINDYVEKGSQGKIVDLVDE<br>LDKDTVFALVNYIFFKGKWEKPFEVEQTTEEDFHVDEETTVKVPMMNRLGMFDLHHCDKLSSWVLL<br>MDYVGNATAFFILPDQGKLHQLEDMLTKEILAKFLEKRYPSSANLHLPKLTISGTYDLKSLLGNLGITK<br>VFSDEADLSGVTEEQPLKLSKGNGIFHHMGLSAAGGPVQPGPWLPASIQACHLQAPTLLQHSSQTGQ<br>LYLQIVPRTGLSVQKQHHLLPREHRFSLSDALPGDQRGHSYPDPVGHRLQV	
5	Alpha-1-antitrypsin	A0A481ATB2	No data	No data	Match to NLHLP at 312<br>MASSSTWGLLLLAGLCCLVPISLAEGLQGHAVQETDVPRHDHEQHQEAACH<br>RIAPNLADFAFSLYRQVARQSNTSNIFLSPVSIATAFAMLSLGTKGATHAEILE<br>GLQFNLTEKAEAEIHEGFQHLLHTLNQPDNQLQLTTGNGLFIDEKAKLVPKF<br>LEDVKNLYHSEAFSINFRDTEEAKKCINDYVEKGSQGKIVDLVDELDKDTVFA<br>LVNYIFFKGKWEKPFEVEQTTEEDFHVDEETTVKVPMMNRLGMFDLHHCEK<br>LSSWVLLMDYVGNAPAFFILPDQGKLHQLEDMLTKEILAKFLEKRYPSSANL<br>HLPKLTISGTYDLKSLLGNLGITKVFSDEADLSGVTEEQQPLKLSKALHKAVLD<br>LSENGTDHGGDILSKDTRWSNHQTISFNVPFLILIKDENTNIPLFMGRVVNPL<br>QN	90% with P50447 (2)
6	Alpha-1-antitrypsin	A0A4X1SHX2	No data	No data	Match to NLHLP at 312<br>MASSSTWGLLLLAGLCCLVPISLAEGLQGHAVQETDVPRHDHEQHQEAACH<br>RIAPNLADFAFSLYRQVARQSNTSNIFLSPVSIATAFAMLSLGTKGATHAEILE<br>GLQFNLTEKAEAEIHEGFQHLLHTLNQPDNQLQLTTGNGLFIDEKAKLVPKF<br>LEDVKNLYHSEAFSINFRDTEEAKKCINDYVEKGSQGKIVDLVDELDKDTVFA<br>LVNYIFFKGKWEKPFEVEQTTEEDFHVDEETTVKVPMMNRLGMFDLHHCDK<br>LSSWVLLMDYVGNATAFFILPDQGKLHQLEDMLTKEILAKFLEKRYPSSANL<br>HLPKLTISGTYDLKSLLGNLGITKVFSDEADLSGVTEEQPLKLSKALHRAVLTI<br>DEKGTEATGATILEAIPMSIPPNVKFNKPFLFLIYDTKTKAVLFMGKVMNPTQ<br>K	90% with P50447 (2)

7	Cytochrome P450 21-hydroxylase (фрагмент)	Q6B7Q0	No data	GO:0020037<br>GO:0005506<br>GO:0016705	Match to NLHLP at 27<br>MVLVGLLLLLTLLAGARLLWGQWKLRNLHLPPLVPGFLHLLQPNLPIYLLGLTQRLGPIYRLRLGLQD<br>VVVLNSKRTIEEALVRKWVDFAGRPQIPSYKLASQHCPDISLGDYSLFWKAHKKFTRSALLLGVRSSM<br>EPRVEQLTQEFCE	
8	Cytochrome P450 21-hydroxylase (фрагмент)	Q6B7P9	No data	The same (7)	Match to NLHLP at 27<br>MVLVGLLLLLTLLAGARLLWGQWKLRNLHLPPLVPGFLHLLQPNLPIYLLGLTQRLGPIYRLRLGLQD<br>VVVLNSKRTIEEALVRKWVDFAGRPQIPSYKLASQHCPDISLGDYSLFWKAHKKLTRSALLLGVRSSM<br>EPRVEQLTQEFCE	90% with Q6B7Q0 (7)
9	Cytochrome P450 21-hydroxylase (фрагмент)	Q6B7Q2	No data	The same (7)	Match to NLHLP at 27<br>MVLVGLLLLLTLLAGARLLWGQWKLRNLHLPPLVPGFLHLLQPNLPIYLLGLTQRLGPIYRLRLGLQD<br>VVVLNSKRTIEEALVRKWVDFAGRPQIPSYKLASQHCPDISLGDYSLFWKAHKKFTRSALLLGVRSSM<br>EPRVEQLTQEFCERMRAQAGTPVTIQKEFSVLTCS	90% with Q6B7Q0 (7)
10	Cytochrome P450 21-hydroxylase (фрагмент)	Q6B7Q1	No data	The same (7)	Match to NLHLP at 27<br>MVLVWLLLLLTLLAGARLLWGQWKLRNLHLPPLVPGFLHLLQPNLPIYLLGLTQRLGPIYRLRLGLQ<br>DVVVLNSKRTIEEALVRKWVDFAGRPQIPSYKLASQHCPDISLGDYSLFWKAHKKLTRSALLLGVRSS<br>MEPRVEQLTQEFCE	90% with P15540 (1)
11	Cytochrome P450 21-hydroxylase (фрагмент)	Q6B7P7	No data	The same (7)	Match to NLHLP at 27<br>MVLVWLLLLLTLLAGARLLWGQWKLRNLHLPPLVPGFLHLLQPNLPIYLLGLTQRLGPIYRLRLGLQ<br>DVVVLNSKRTIEEALVRKWVDFAGRPQIPSYKLASQHCPDISLGDYSLFWKAHKKLTRSALLLGVRSS<br>MEPRVEQLTQEFCERMRAQAGTPVTIQKEFSVLTCS	100% with Q6B7Q1 (10)<br>90% with P15540 (1)
12	Cytochrome P450 21-hydroxylase (фрагмент)	Q6B7P8	No data	The same (7)	Match to NLHLP at 27<br>MVLVGLLLLLTLLAGARLLWGQWKLRNLHLPPLVPGFLHLLQPNLPIYLLGLTQRLGPIYRLRLGLQD<br>VVVLNSKRTIEEALVRKWVDFAGRPQIPSYKLASQHCPDISLGDYSLFWKAHKKLTRSALLLGVRSSM<br>EPRVEQLTQEFCERMRAQAGTPVTIQKEFSVLTCS	100% with Q6B7P9 (8)<br>90% with Q6B7Q0 (7)
13	Cytochrome P450, family 21, subfamily A, polypepide 2	A5A8W7	Low in endocardial endothelium (41.74)	The same (7) + GO:0004497	Match to NLHLP at 27<br>MVLVWLLLLLTLLAGARLLWGQWKLRNLHLPPLVPGFLHLLQPNLPIYLLGLTQRLGPIYRLRLGLQ<br>DKLASQHCPDISLGDYSLFWKAHKKLTRSALLLGVRSSMEPRVEQLTQEFCERMRAQAGTPVTIQKEF<br>SVLTCSIICCLTFGDKVKEDTLVHALHDCVQDLMKTWEHWSIQILDMVPFLRFFPSPGLRRLKQAIEN<br>RDHLVEKQLRRHKESMVAGQWRDMLDYMLQEAGRQRVEEGQGQLLEGHVHMSVVDLFIGGTETT<br>ANTLSWAVVYLLHHPEIQWRLQEELDRELGPGAAGSRVPYKDRARLPLLNATIAEVLRLRPVVPLAL<br>PHRATRPSSIFGYDIPEGTVVIPNLQGAHLDETVWEQPHEFRPDRFLAPGANPSALAFGCGARVCLGE<br>PLARLELFVVLVQLLQAFTLLPPEGALPSLQPHPHSGINLKVQPFQVRLQPRGGRGEGPGPR	
14	Cytochrome P450, family 21, subfamily A, polypepide 2	A5A8W5	The same (13)	The same (13)	Match to NLHLP at 27<br>MVLVWLLLLLTLLAGARLLWGQWKLRNLHLPPLVPGFLHLLQPNLPIYLLGLTQRLGPIYRLRLGLQ<br>DVVVLNSKRTIEEALVRKWVDFAGRPQIPSYKLASQHCPDISLGDYSLFWKAHKKLTRSALLLGVRSS<br>MEPRVEQLTQEFCERMRAQAGTPVTIQKEFSVLTCSIICCLTFGDKEDTLVHALHDCVQDLMKTWEH<br>WSIQILDMVPFLRFFPSPGLRRLKQAIENRDHLVEKQLRRHKESMVAGQWRDMLDYMLQEAGRQRV<br>EEGQGQLLEGHVHMSVVDLFIGGTETTANTLSWAVVYLLHHPEIQWRLQEELDRELGPGAAGSRVP<br>YKDRARLPLLNATIAEVLRLRPVVPLALPHRATRPSSIFGYDIPEGTVVIPNLQGAHLDETVWEQPHEF<br>RPDRFLAPGANPSALAFGCGARVCLGEPLARLELFVVLVQLLQAFTLLPPEGALPSLQPHPHSGINLK<br>VQPFQD	90% with P15540 (1), Q6B7Q1 (10) and Q6B7P7 (11)

15	Protein kinase domain-containing protein	A0A4X1WBG5	No data	GO:0004672<br>GO:0005524<br>GO:0006468	Match to NLHLP at 146<br>MDRSKRNSIAGFPPRVERLEDFEGGGGGGDGSLSHVGRVWPSSYRALISAFSRLTRLDDFTCEKIGSGF<br>FSEVFKVRHRASGQVMALKMNTLSSNRANMLKEVQLMNRLSHPNILRFMGVCVHQGQLHALTEYI<br>NSGNLEQLLDSNLHLPWTVRVKLAYDIAVGLSYLHFKGIFHRDLTSKNCLIKRDESGYSAVVADFGLA<br>EKIPDGSMGSEKLAVVGSPFWMAPEVLRDEPYNEKADVFSYGIILCEIIARIQADPDYLPRTEMDPKLR<br>PSFAEIGKTLEEILSRLQEEELERDRKLQPAAKGLLEKGPGLKRLSLQDNKIPPKSPRPRRTIWLSRSQSD<br>IFSSKPPRTVNVLDPYYQPRGGAARAPKINPFSARQDLKGGKIKFFDLPSKSVISLVFDLDAPGPGSTPV<br>AEWQEPLAPPARRWRSLPGSPEFLHQEACPFVGEESLSDGPPPRLSSHKYRVREIPPFRAPALPAAPAH<br>EAMDCSGPQEENGFGPRPPGPGLCLAGALEEMEVEERPRDLAVSGTGLKTQGEQDG	
16	Protein kinase domain-containing protein	A0A4X1WA11	No data	The same (15)	Match to NLHLP at 146<br>MDRSKRNSIAGFPPRVERLEDFEGGGGGGDGSLSHVGRVWPSSYRALISAFSRLTRLDDFTCEKIGSGF<br>FSEVFKVRHRASGQVMALKMNTLSSNRANMLKEVQLMNRLSHPNILRFMGVCVHQGQLHALTEYI<br>NSGNLEQLLDSNLHLPWTVRVKLAYDIAVGLSYLHFKGIFHRDLTSKNCLIKRDESGYSAVVADFGLA<br>EKIPDGSMGSEKLAVVGSPFWMAPEVLRDEPYNEKADVFSYGIILCEIIARIQADPDYLPRTENFGLDY<br>DAFQHMVGDCPPDFLQLTFNCCNMDPKLRPSFAEIGKTLEEILSRLQEEELERDRKLQPAAKGLLEKG<br>PGLKRLSLQDNKIPPKSPRPRRTIWLSRSQSDIFSSKPPRTVNVLDPYYQPRGGAARAPKINPFSARQDL<br>KGGKIKFFDLPSKSVISLVFDLDAPGPGSTPVAEWQEPLAPPARRWRSLPGSPEFLHQEACPFVGEESLS<br>DGPPPRLSSHKYRVREIPPFRAPALPAAPAHEAMDCSGPQEENGFGPRPPGPGLCLAGALEEMEVEER<br>PRDLAVSGTGLKTQGEQDG	
17	DNA-directed RNA polymerase subunit	A0A4X1SL14	Medium-low in heart left ventricle	GO:0003677<br>GO:0003899<br>GO:0006351	Match to NLHLP at 400<br>MICKTCCHIMLSQEERKQFLDYLKRPGLTYLQKRGLKKKISDKCRKKNTCHHCGAFNGTVKKCGLLK<br>IIHEKYKTNKKVVDPIVSNFLQSFETAIEHNKEVEPLLGRAQENLNPLVVLNLFKRIPAEDIPLLLMNPE<br>AGKPSDLILTRLLVPPLCIRPSVVSDLKSGTNEDDLTMKLTEIIFLNDVIKKHRISGAKTQMIMEDWDFL<br>QLQCALYINSELSGIPLNMAPKKWTRGFVQRLKGKQGRFRGNLSGKRVDFSGRTVISPDPNLRIDEVA<br>VPVHVAKILTFPEKVNKANITFLRKLVRNGPEVHPGANFIQQRHTQMKRFLKYGNREKMAQELKFG<br>DIVERHLIDGDVVLFNRQPSLHKLSIMAHLARVKPHRTFRFNECVCTPYNADFDGDEMNLHLPQTEE<br>AKAEALVLMGTKANLVTPRNGEPLIAAIQDFLTGAYLLTLKDTFFDRAKACQIIASILVGKDEKIKVRL<br>PPPTILKPVTLWTGKQVFSVILRPSYDNPVRANLRTKGKQYCGKGEDLCVNDSYVTIQNSELMSGSMD<br>KGTLGSGSKNNIFYILLRDWGQNEAADAMSRLARLAPVYLSNRGFSIGIGDVTPGQGLLKAKYELLN<br>AGYRKCDEYIEALNTGKLQQQPGCTAEETLEALILKELSVIRDHAGSACLRELDKSNSPLTMALCGSK<br>GSFINISQMIACVGQQAISGSRVPDGFENRSLPHFEKHSKLPAAKGFVANSFYSGLTPTEFFFHTMAGR<br>EGLVDTAVKTAETGYMQRRLVKSLEDLCSQYDLTVRSSTGDIIQFIYGGDGLDPAAMEGKDEPLEFKR<br>VLDNIKAVFPCQSEPALSKNELLLTTESIMKKNEFLCCQDSFLQEIKKFIKGVSEKIKKTRDKYGINDNG<br>TTEPRVLYQLDRITPTQIEKFLETCRDKYMRAQMEPGSAVGALCAQSIGEPGTQMTLKTFHFAGVASM<br>NITLGVPRIKEIINASKAISTPIITAQLDKDDDADYARLVKGRIEKTLLGEISEYIEEVFLPDDCFILIKLSLE<br>RIRLLLRLEVNAETVRYSICMSKLRVKPGDVAVHGEAVVCVTPRENSKSSMYYVLQFLKEDLPKVVVQ<br>GIPEVSRAVIHIDEQSGKEKYKLLVEGDNLRAVMATHGVKGTQTTSNNTYEVEKTLGIEAARTTIINEI<br>QYTMVNHGMSIDRRHVMLLSDLMTYKGEVLGITRFGLAKMKESVLMLASFEKTADHLFDAAYFGQ<br>KDSVCGVSECIIMGIPMNIGTGLFKLLHKANRDPSPPRRPLIFDTDEFHIPLVT	
18	DNA-directed RNA polymerase subunit	A0A286ZSI9	The same (17)	The same (17)+<br>GO:0003682<br>GO:0045087<br>GO:0032728	Match to NLHLP at 506<br>MVKEQFRETDVAKKISHICFGMKSAEEMRQQAHIQVVSKNLYSQDNNHSPLLYGVLDHRMGTSEKD<br>RPCETCGKNLADCLGHYGYIDLELPCFHVGYFRAVIGILQMICKTCCHIMLSQEERKQFLDYLKRPGL<br>TYLQKRGLKKKISDKCRKKNTCHHCGAFNGTVKKCGLLKIIHEKYKTNKKVVDPIVSNFLQSFETAIE<br>HNKEVEPLLGRAQENLNPLVVLNLFKRIPAEDIPLLLMNPEAGKPSDLILTRLLVPPLCIRPSVVSDLKS<br>GTNEDDLTMKLTEIIFLNDVIKKHRISGAKTQMIMEDWDFLQLQCALYINSELSGIPLNMAPKKWTRG<br>FVQRLKGKQGRFRGNLSGKRVDFSGRTVISPDPNLRIDEVAVPVHVAKILTFPEKVNKANITFLRKLVR<br>NGPEVHPGANFIQQRHTQMKRFLKYGNREKMAQELKFGDIVERHLIDGDVVLFNRQPSLHKLSIMA<br>HLARVKPHRTFRFNECVCTPYNADFDGDEMNLHLPQTEEAKAEALVLMGTKANLVTPRNGEPLIAA	

					IQDFLTGAYLLTLKDTFFDRAKACQIIASILVGKDEKIKVRLPPPTILKPVTLWTGKQVFSVILRPSYDNP<br>VRANLRTKGKQYCGKGEDLCVNDSYVTIQNSELMSGSMDKGTLGSGSKNNIFYILLRDWGQNEAAD<br>AMSRLARLAPVYLSNRGFSIGIGDVTPGQGLLKAKYELLNAGYRKCDEYIEALNTGKLQQQPGCTAE<br>ETLEALILKELSVIRDHAGSACLRELDKSNSPLTMALCGSKGSFINISQMIACVGQQAISGSRVPDGFEN<br>RSLPHFEKHSKLPAAKGFVANSFYSGLTPTEFFFHTMAGREGLVDTAVKTAETGYMQRRLVKSLEDLC<br>SQYDLTVRSSTGDIIQFIYGGDGLDPAAMEGKDEPLEFKRVLDNIKAVFPCQSEPALSKNELLLTTESIM<br>KKNEFLCCQDSFLQTLTETGYEKYNEEIKKFIKGVSEKIKKTRDKYGINDNGTTEPRVLYQLDRITPTQI<br>EKFLETCRDKYMRAQMEPGSAVGALCAQSIGEPGTQMTLKTFHFAGVASMNITLGVPRIKEIINASKA<br>ISTPIITAQLDKDDDADYARLVKGRIEKTLLGEISEYIEEVFLPDDCFILIKLSLERIRLLRLEVNAETVRYSI<br>CMSKLRVKPGDVAVHGEAVVCVTPRENSKSSMYYVLQFLKEDLPKVVVQGIPEVSRAVIHIDEQSGK<br>EKYKLLVEGDNLRAVMATHGVKGTQTTSNNTYEVEKTLGIEAARTTIINEIQYTMVNHGMSIDRRHV<br>MLLSDLMTYKGEVLGITRFGLAKMKESVLMLASFEKTADHLFDAAYFGQKDSVCGVSECIIMGIPMNI<br>GTGLFKLLHKANRDPSPPRRPLIFDTDEFHIPLVT	
19	DNA-directed RNA polymerase subunit	A0A4X1SK18	No data	The same (17)	Match to NLHLP at 506<br>MVKEQFRETDVAKKISHICFGMKSAEEMRQQAHIQVVSKNLYSQDNNHSPLLYGVLDHRMGTSEKD<br>RPCETCGKNLADCLGHYGYIDLELPCFHVGYFRAVIGILQMICKTCCHIMLSQEERKQFLDYLKRPGL<br>TYLQKRGLKKKISDKCRKKNTCHHCGAFNGTVKKCGLLKIIHEKYKTNKKVVDPIVSNFLQSFETAIE<br>HNKEVEPLLGRAQENLNPLVVLNLFKRIPAEDIPLLLMNPEAGKPSDLILTRLLVPPLCIRPSVVSDLKS<br>GTNEDDLTMKLTEIIFLNDVIKKHRISGAKTQMIMEDWDFLQLQCALYINSELSGIPLNMAPKKWTRG<br>FVQRLKGKQGRFRGNLSGKRVDFSGRTVISPDPNLRIDEVAVPVHVAKILTFPEKVNKANITFLRKLVR<br>NGPEVHPGANFIQQRHTQMKRFLKYGNREKMAQELKFGDIVERHLIDGDVVLFNRQPSLHKLSIMA<br>HLARVKPHRTFRFNECVCTPYNADFDGDEMNLHLPQTEEAKAEALVLMGTKANLVTPRNGEPLIAA<br>IQDFLTGAYLLTLKDTFFDRAKACQIIASILVGKDEKIKVRLPPPTILKPVTLWTGKQVFSVILRPSYDNP<br>VRANLRTKGKQYCGKGEDLCVNDSYVTIQNSELMSGSMDKGTLGSGSKNNIFYILLRDWGQNEAAD<br>AMSRLARLAPVYLSNRGFSIGIGDVTPGQGLLKAKYELLNAGYRKCDEYIEALNTGKLQQQPGCTAE<br>ETLEALILKELSVIRDHAGSACLRELDKSNSPLTMALCGSKGSFINISQMIACVGQQAISGSRVPDGFEN<br>RSLPHFEKHSKLPAAKGFVANSFYSGLTPTEFFFHTMAGREGLVDTAVKTAETGYMQRRLVKSLEDLC<br>SQYDLTVRSSTGDIIQFIYGGDGLDPAAMEGKDEPLEFKRVLDNIKAVFPCQSEPALSKNELLLTTESIM<br>KKNEFLCCQDSFLQEIKKFIKGVSEKIKKTRDKYGINDNGTTEPRVLYQLDRITPTQIEKFLETCRDKYM<br>RAQMEPGSAVGALCAQSIGEPGTQMTLKTFHFAGVASMNITLGVPRIKEIINASKAISTPIITAQLDKD<br>DDADYARLVKGRIEKTLLGEISEYIEEVFLPDDCFILIKLSLERIRLLRLEVNAETVRYSICMSKLRVKPGD<br>VAVHGEAVVCVTPRENSKSSMYYVLQFLKEDLPKGEVLGITRFGLAKMKESVLMLASFEKTADHLFD<br>AAYFGQKDSVCGVSECIIMGIPMNIGTGLFKLLHKANRDPSPPRRPLIFDTDEFHIPLVT	
20	DNA-directed RNA polymerase subunit	A0A4X1SJU1	The same (17)	The same (17)	Match to NLHLP at 506<br>MVKEQFRETDVAKKISHICFGMKSAEEMRQQAHIQVVSKNLYSQDNNHSPLLYGVLDHRMGTSEKD<br>RPCETCGKNLADCLGHYGYIDLELPCFHVGYFRAVIGILQMICKTCCHIMLSQEERKQFLDYLKRPGL<br>TYLQKRGLKKKISDKCRKKNTCHHCGAFNGTVKKCGLLKIIHEKYKTNKKVVDPIVSNFLQSFETAIE<br>HNKEVEPLLGRAQENLNPLVVLNLFKRIPAEDIPLLLMNPEAGKPSDLILTRLLVPPLCIRPSVVSDLKS<br>GTNEDDLTMKLTEIIFLNDVIKKHRISGAKTQMIMEDWDFLQLQCALYINSELSGIPLNMAPKKWTRG<br>FVQRLKGKQGRFRGNLSGKRVDFSGRTVISPDPNLRIDEVAVPVHVAKILTFPEKVNKANITFLRKLVR<br>NGPEVHPGANFIQQRHTQMKRFLKYGNREKMAQELKFGDIVERHLIDGDVVLFNRQPSLHKLSIMA<br>HLARVKPHRTFRFNECVCTPYNADFDGDEMNLHLPQTEEAKAEALVLMGTKANLVTPRNGEPLIAA<br>IQDFLTGAYLLTLKDTFFDRAKACQIIASILVGKDEKIKVRLPPPTILKPVTLWTGKQVFSVILRPSYDNP<br>VRANLRTKGKQYCGKGEDLCVNDSYVTIQNSELMSGSMDKGTLGSGSKNNIFYILLRDWGQNEAAD<br>AMSRLARLAPVYLSNRGFSIGIGDVTPGQGLLKAKYELLNAGYRKCDEYIEALNTGKLQQQPGCTAE<br>ETLEALILKELSVIRDHAGSACLRELDKSNSPLTMALCGSKGSFINISQMIACVGQQAISGSRVPDGFEN<br>RSLPHFEKHSKLPAAKGFVANSFYSGLTPTEFFFHTMAGREGLVDTAVKTAETGYMQRRLVKSLEDLC<br>SQYDLTVRSSTGDIIQFIYGGDGLDPAAMEGKDEPLEFKRVLDNIKAVFPCQSEPALSKNELLLTTESIM	100% with A0A4X1SL14 (17);<br>90% with A0A286ZSI9 (18)

					KKNEFLCCQDSFLQEIKKFIKGVSEKIKKTRDKYGINDNGTTEPRVLYQLDRITPTQIEKFLETCRDKYM<br>RAQMEPGSAVGALCAQSIGEPGTQMTLKTFHFAGVASMNITLGVPRIKEIINASKAISTPIITAQLDKD<br>DDADYARLVKGRIEKTLLGEISEYIEEVFLPDDCFILIKLSLERIRLLRLEVNAETVRYSICMSKLRVKPGD<br>VAVHGEAVVCVTPRENSKSSMYYVLQFLKEDLPKVVVQGIPEVSRAVIHIDEQSGKEKYKLLVEGDNL<br>RAVMATHGVKGTQTTSNNTYEVEKTLGIEAARTTIINEIQYTMVNHGMSIDRRHVMLLSDLMTYKGE<br>VLGITRFGLAKMKESVLMLASFEKTADHLFDAAYFGQKDSVCGVSECIIMGIPMNIGTGLFKLLHKAN<br>RDPSPPRRPLIFDTDEFHIPLVT	
21	DNA-directed RNA polymerase subunit	A0A4X1V3A0	No data	The same (17)	Match to NLHLP at 502<br>MHGGGPPSGDSACPLRTIKRVQFGVLSPDELKRMSVTEGGIKYPETTEGGRPKLGGLMDPRQGVIERT<br>GRCQTCAGNMTECPGHFGHIELAKPVFHVGFLVKTMKVLRCVCFFCSKLLVDSNNPKIKDILAKSKG<br>QPKKRLTHVYDLCKGKNICEGGEEMDNKFGVEQPEGDEDLTKEKGHGGCGRYQPRIRRSGLELYAE<br>WKHVNEDSQEKKILLSPERVHEIFKRISDEECFVLGMEPRYARPEWMIVTVLPVPPLSVRPAVVMQGS<br>ARNQDDLTHKLADIVKINNQLRRNEQNGAAAHVIAEDVKLLQFHVATMVDNELPGLPRAMQKSG<br>RPLKSLKQRLKGKEGRVRGNLMGKRVDFSARTVITPDPNLSIDQVGVPRSIAANMTFAEIVTPFNIDRL<br>QELVRRGNSQYPGAKYIIRDNGDRIDLRFHPKPSDLHLQTGYKVERHMCDGDIVIFNRQPTLHKMSM<br>MGHRVRILPWSTFRLNLSVTTPYNADFDGDEMNLHLPQSLETRAEIQELAMVPRMIVTPQSNPVMGI<br>VQDTLTAVRKFTKRDVFLERVCGPTGDLAW	
22	DNA-directed RNA polymerase subunit	A0A286ZPB2	The same (17)	The same (17)	Match to NLHLP at 400<br>MICKTCCHIMLSQEERKQFLDYLKRPGLTYLQKRGLKKKISDKCRKKNTCHHCGAFNGTVKKCGLLK<br>IIHEKYKTNKKVVDPIVSNFLQSFETAIEHNKEVEPLLGRAQENLNPLVVLNLFKRIPAEDIPLLLMNPE<br>AGKPSDLILTRLLVPPLCIRPSVVSDLKSGTNEDDLTMKLTEIIFLNDVIKKHRISGAKTQMIMEDWDFL<br>QLQCALYINSELSGIPLNMAPKKWTRGFVQRLKGKQGRFRGNLSGKRVDFSGRTVISPDPNLRIDEVA<br>VPVHVAKILTFPEKVNKANITFLRKLVRNGPEVHPGANFIQQRHTQMKRFLKYGNREKMAQELKFG<br>DIVERHLIDGDVVLFNRQPSLHKLSIMAHLARVKPHRTFRFNECVCTPYNADFDGDEMNLHLPQTEE<br>AKAEALVLMGTKANLVTPRNGEPLIAAIQDFLTGAYLLTLKDTFFDRAKACQIIASILVGKDEKIKVRL<br>PPPTILKPVTLWTGKQVFSVILRPSYDNPVRANLRTKGKQYCGKGEDLCVNDSYVTIQNSELMSGSMD<br>KGTLGSGSKNNIFYILLRDWGQNEAADAMSRLARLAPVYLSNRGFSIGIGDVTPGQGLLKAKYELLN<br>AGYRKCDEYIEALNTGKLQQQPGCTAEETLEALILKELSVIRDHAGSACLRELDKSNSPLTMALCGSK<br>GSFINISQMIACVGQQAISGSRVPDGFENRSLPHFEKHSKLPAAKFVANSFYSGLTPTEFFFHTMAGRE<br>GLVDTAVKTAETGYMQRRLVKSLEDLCSQYDLTVRSSTGDIIQFIYGGDGLDPAAMEGKDEPLEFKRV<br>LDNIKAVFPCQSEPALSKNELLLTTESIMKKNEFLCCQDSFLQEIKKFIKGVSEKIKKTRDKYGINDNGT<br>TEPRVLYQLDRITPTQIEKFLETCRDKYMRAQMEPGSAVGALCAQSIGEPGTQMTLKTFHFAGVASM<br>NITLGVPRIKEIINASKAISTPIITAQLDKDDDADYARLVKGRIEKTLLGEISEYIEEVFLPDDCFILIKLSLE<br>RIRLLRLEVNAETVRYSICMSKLRVKPGDVAVHGEAVVCVTPRENSKSSMYYVLQFLKEDLPKVVVQ<br>GIPEVSRAVIHIDEQSGKEKYKLLVEGDNLRAVMATHGVKGTQTTSNNTYEVEKTLGIEAARTTIINEI<br>QYTMVNHGMSIDRRHVMLLSDLMTYKGEVLGITRFGLAKMKESVLMLASFEKTADHLFDAAYFGQ<br>KDSVCGVSECIIMGIPMNIGTGLFKLLHKANRDPSPPRRPLIFDTDEFHIPLVT	100% with A0A4X1SJU1 (20) and A0A4X1SL14 (17)<br>90% with A0A4X1SL14 (17), A0A286ZSI9 (18) and A0A4X1SJU1 (20)
23	DNA-directed RNA polymerase subunit	A0A480MHM1	No data	The same (17)	Match to NLHLP at 506<br>MVKEQFRETDVAKKISHICFGMKSAEEMRQQAHIQVVSKNLYSQDNNHSPLLYGVLDHRMGTSEKD<br>RPCETCGKNLADCLGHYGYIDLELPCFHVGYFRAVIGILQMICKTCCHIMLSQEERKQFLDYLKRPGL<br>TYLQKRGLKKKISDKCRKKNTCHHCGAFNGTVKKCGLLKIIHEKYKTNKKVVDPIVSNFLQSFETAIE<br>HNKEVEPLLGRAQENLNPLVVLNLFKRIPAEDIPLLLMNPEAGKPSDLILTRLLVPPLCIRPSVVSDLKS<br>GTNEDDLTMKLTEIIFLNDVIKKHRISGAKTQMIMEDWDFLQLQCALYINSELSGIPLNMAPKKWTRG<br>FVQRLKGKQGRFRGNLSGKRVDFSGRTVISPDPNLRIDEVAVPVHVAKILTFPEKVNKANITFLRKLVR<br>NGPEVHPGANFIQQRHTQMKRFLKYGNREKMAQELKFGDIVERHLIDGDVVLFNRQPSLHKLSIMA<br>HLARVKPHRTFRFNECVCTPYNADFDGDEMNLHLPQTEEAKAEALVLMGTKANLVTPRNGEPLIAA<br>IQDFLTGAYLLTLKDTFFDRAKACQIIASILVGKDEKIKVRLPPPTILKPVTLWTGKQVFSVILRPSYDNP<br>VRANLRTKGKQYCGKGEDLCVNDSYVTIQNSELMSGSMDKGTLGSGSKNNIFYILLRDWGQNEAAD	

					AMSRLARLAPVYLSNRGFSIGIGDVTPGQGLLKAKYELLNAGYRKCDEYIEALNTGKLQQQPGCTAE<br>ETLEALILKELSVIRDHAGSACLRELDKSNSPLTMALCGSKGSFINISQMIACVGQQAISGSRVPDGFEN<br>RSLPHFEKHSKLPAAKGFVANSFYSGLTPTEFFFHTMAGREGLVDTAVKTAETGYMQRRLVKSLEDLC<br>SQYDLTVRSSTGDIIQFIYGGDGLDPAAMEGKDEPLEFKRVLDNIKAVFPCQSEPALSKNELLLTTESIM<br>KKNEFLCCQDSFLQVSSEGPCAQFDCHRGIVHQVLAGTSLSWGY	
24	DNA-directed RNA polymerase subunit	F1S2E6	Medium-low in heart (59.15), endocardial endothelium (58.34), heart left ventricle (55.38)	The same (17)	Match to NLHLP at 506<br>MVKEQFRETDVAKKISHICFGMKSAEEMRQQAHIQVVSKNLYSQDNNHSPLLYGVLDHRMGTSEKD<br>RPCETCGKNLADCLGHYGYIDLELPCFHVGYFRAVIGILQMICKTCCHIMLSQEERKQFLDYLKRPGL<br>TYLQKRGLKKKISDKCRKKNTCHHCGAFNGTVKKCGLLKIIHEKYKTNKKVVDPIVSNFLQSFETAIE<br>HNKEVEPLLGRAQENLNPLVVLNLFKRIPAEDIPLLLMNPEAGKPSDLILTRLLVPPLCIRPSVVSDLKS<br>GTNEDDLTMKLTEIIFLNDVIKKHRISGAKTQMIMEDWDFLQLQCALYINSELSGIPLNMAPKKWTRG<br>FVQRLKGKQGRFRGNLSGKRVDFSGRTVISPDPNLRIDEVAVPVHVAKILTFPEKVNKANITFLRKLVR<br>NGPEVHPGANFIQQRHTQMKRFLKYGNREKMAQELKFGDIVERHLIDGDVVLFNRQPSLHKLSIMA<br>HLARVKPHRTFRFNECVCTPYNADFDGDEMNLHLPQTEEAKAEALVLMGTKANLVTPRNGEPLIAA<br>IQDFLTGAYLLTLKDTFFDRAKACQIIASILVGKDEKIKVRLPPPTILKPVTLWTGKQVFSVILRPSYDNP<br>VRANLRTKGKQYCGKGEDLCVNDSYVTIQNSELMSGSMDKGTLGSGSKNNIFYILLRDWGQNEAAD<br>AMSRLARLAPVYLSNRGFSIGIGDVTPGQGLLKAKYELLNAGYRKCDEYIEALNTGKLQQQPGCTAE<br>ETLEALILKELSVIRDHAGSACLRELDKSNSPLTMALCGSKGSFINISQMIACVGQQAISGSRVPDGFEN<br>RSLPHFEKHSKLPAAKGFVANSFYSGLTPTEFFFHTMAGREGLVDTAVKTAETGYMQRRLVKSLEDLC<br>SQYDLTVRSSTGDIIQFIYGGDGLDPAAMEGKDEPLEFKRVLDNIKAVFPCQSEPALSKNELLLTTESIM<br>KKNEFLCCQDSFLQEIKKFIKGVSEKIKKTRDKYGINDNGTTEPRVLYQLDRITPTQIEKFLETCRDKYM<br>RAQMEPGSAVGALCAQSIGEPGTQMTLKTFHFAGVASMNITLGVPRIKEIINASKAISTPIITAQLDKD<br>DDADYARLVKGRIEKTLLGEISEYIEEVFLPDDCFILIKLSLERIRLLRLEVNAETVRYSICMSKLRVKPGD<br>VAVHGEAVVCVTPRENSKSSMYYVLQFLKEDLPKVVVQGIPEVSRAVIHIDEQSGKEKYKLLVEGDNL<br>RAVMATHGVKGTQTTSNNTYEVEKTLGIEAARTTIINEIQYTMVNHGMSIDRRHVMLLSDLMTYKGE<br>VLGITRFGLAKMKESVLMLASFEKTADHLFDAAYFGQKDSVCGVSECIIMGIPMNIGTGLFKLLHKAN<br>RDPSPPRRPLIFDTDEFHIPLVT	100% with A0A4X1SJU1 (20), A0A4X1SL14 (17) and A0A286ZPB2 (22);<br>90% with A0A4X1SL14 (17), A0A286ZSI9 (18), A0A4X1SJU1 (20) and A0A286ZPB2 (22)
25	DNA-directed RNA polymerase subunit	A0A481CFL6	No data	GO:0046872<br>GO:0006366	Match to NLHLP at 425<br>MTECPGHFGHIELAKPVFHVGFLVKTMKVLRCVCFFCSKLLVDSNNPKIKDILAKSKGQPKKRLTHV<br>YDLCKGKNICEGGEEMDNKFGVEQPEGDEDLTKEKGHGGCGRYQPRIRRSGLELYAEWKHVNEDSQ<br>EKKILLSPERVHEIFKRISDEECFVLGMEPRYARPEWMIVTVLPVPPLSVRPAVVMQGSARNQDDLTH<br>KLADIVKINNQLRRNEQNGAAAHVIAEDVKLLQFHVATMVDNELPGLPRAMQKSGRPLKSLKQRL<br>KGKEGRVRGNLMGKRVDFSARTVITPDPNLSIDQVGVPRSIAANMTFAEIVTPFNIDRLQELVRRGNS<br>QYPGAKYIIRDNGDRIDLRFHPKPSDLHLQTGYKVERHMCDGDIVIFNRQPTLHKMSMMGHRVRILP<br>WSTFRLNLSVTTPYNADFDGDEMNLHLPQSLETRAEIQELAMVPRMIVTPQSNRPVMGIVQDTLTAV<br>RKFTKRDVFLERGEVMNLLMFLSTWDGKVPQPAILKPRPLWTGKQIFSLIIPGHINCIRTHSTHPDDED<br>SGPYKHISPGDTKVVVENGELIMGILCKKSLGTSAGSLVHISYLEMGHDITRLFYSNIQTVINNWLLIEG<br>HTIGIGDSIADSKTYQDIQNTIKKAKQDVIEVIEKAHNNELEPTPGNTLRQTFENQVNRILNDARDKT<br>GSSAQKSLSEYNNFKSMVVSGAKGSKINISQVIAVVGQQNVEGKRIPFGFKHRTLPHFIKDDYGPESRG<br>FVENSYLAGLTPTEFFFHAMGGREGLIDTAVKTAETGYIQRRLIKSMESVMVKYDATVRNSINQVVQL<br>RYGEDGLAGESVEFQNLATLKPSNKAFEKKFRFDYTNERALRRTLQEDLVKDVLSNAHIQNELEREFE<br>RMREDREVLRVIFPTGDSKVVLPCNLLRMIWNAQKIFHINPRLPSDLHPIKVVEGVKELSKKLVIVNG<br>DDPLSRQAQENATLLFNIHLRSTLCSRRMAEEFRLSGEAFDWLLGEIESKFNQAIAHPGEMVGALAA<br>QSLGEPATQMTLNTFHYAGVSAKNVTLGVPRLKELINISKKPKTPSLTVFLLGQSARDAERAKDILCRL<br>EHTTLRKVTANTAIYYDPNPQSTVVAEDQEWVNVYYEMPDFDVARISPWLLRVELDRKHMTDRKLT<br>MEQIAEKINAGFGDDLNCIFNDDNAEKLVLRIRIMNSDENKMQEEEEVVDKMDDDVFLRCIESNML<br>TDMTLQGIEQISKVYMHLPQTDNKKKIIITEDGEFKALQEWILETDGVSLMRVLSEKDVDPVRTTSNDI<br>VEIFTVLGIEAVRKALERELYHVISFDGSYVNYRHLALLCDTMTCRGHLMAITRHGVNRQDTGPLMK	

					CSFEETVDVLMEAAAHGESDPMKGVSENIMLGQLAPAGTGCFDLLLDAEKCKYGMEIPTNIPGLGA<br>AGPTGMFFGSAPSPMGGISPAMTPWNQGATPAYGAWSPSVGSGMTPGAAGFSPSAASDASGFSPGY<br>SPAWSPTPGSPGSPGPSSPYIPSPGGAMSPSYSPTSPAYEPRSPGGYTPQSPSYSPTSPSYSPTSPSYSPTSPN<br>YSPTSPSYSPTSPSYSPTSPSYSPTSPSYSPTSPSYSPTSPSYSPTSPSYSPTSPSYSPTSPSYSPTSPNYSPTSPNY<br>TPTSPSYSPTSPSYSPTSPNYTPTSPNYSPTSPSYSPTSPSYSPTSPSYSPSSPRYTPQSPTYTPSSPSYSPSSPSY<br>SPTSPKYTPTSPSYSPSSPEYTPTSPKYSPTSPKYSPTSPKYSPTSPTYSPTTPKYSPTSPTYSPTSPVYTPTSPK<br>YSPTSPTYSPTSPKYSPTS	
26	DNA-directed RNA polymerase subunit	A0A480L5T3	No data	The same (25)	Match to NLHLP at 502<br>MHGGGPPSGDSACPLRTIKRVQFGVLSPDELKRMSVTEGGIKYPETTEGGRPKLGGLMDPRQGVIERT<br>GRCQTCAGNMTECPGHFGHIELAKPVFHVGFLVKTMKVLRCVCFFCSKLLVDSNNPKIKDILAKSKG<br>QPKKRLTHVYDLCKGKNICEGGEEMDNKFGVEQPEGDEDLTKEKGHGGCGRYQPRIRRSGLELYAE<br>WKHVNEDSQEKKILLSPERVHEIFKRISDEECFVLGMEPRYARPEWMIVTVLPVPPLSVRPAVVMQGS<br>ARNQDDLTHKLADIVKINNQLRRNEQNGAAAHVIAEDVKLLQFHVATMVDNELPGLPRAMQKSG<br>RPLKSLKQRLKGKEGRVRGNLMGKRVDFSARTVITPDPNLSIDQVGVPRSIAANMTFAEIVTPFNIDRL<br>QELVRRGNSQYPGAKYIIRDNGDRIDLRFHPKPSDLHLQTGYKVERHMCDGDIVIFNRQPTLHKMSM<br>MGHRVRILPWSTFRLNLSVTTPYNADFDGDEMNLHLPQSLETRAEIQELAMVPRMIVTPQSNRPVMG<br>IVQDTLTAVRKFTKRDVFLERGEVMNLLMFLSTWDGKVPQPAILKPRPLWTGKQIFSLIIPGHINCIRT<br>HSTHPDDEDSGPYKHISPGDTKVVVENGELIMGILCKKSLGTSAGSLVHISYLEMGHDITRLFYSNIQT<br>VINNWLLIEGHTIGIGDSIADSKTYQDIQNTIKKAKQDVIEVIEKAHNNELEPTPGNTLRQTFENQVNR<br>ILNDARDKTGSSAQKSLSEYNNFKSMVVSGAKGSKINISQVIAVVGQQNVEGKRIPFGFKHRTLPHFIK<br>DDYGPESRGFVENSYLAGLTPTEFFFHAMGGREGLIDTAVKTAETGYIQRRLIKSMESVMVKYDATVR<br>NSINQVVQLRYGEDGLAGESVEFQNLATLKPSNKAFEKKFRFDYTNERALRRTLQEDLVKDVLSNAH<br>IQNELEREFERMREDREVLRVIFPTGDSKVVLPCNLLRMIWNAQKIFHINPRLPSDLHPIKVVEGVKEL<br>SKKLVIVNGDDPLSRQAQENATLLFNIHLRSTLCSRRMAEEFRLSGEAFDWLLGEIESKFNQAIAHPGE<br>MVGALAAQSLGEPATQMTLNTFHYAGVSAKNVTLGVPRLKELINISKKPKTPSLTVFLLGQSARDAE<br>RAKDILCRLEHTTLRKVTANTAIYYDPNPQSTVVAEDQEWVNVYYEMPDFDVARISPWLLRVELDRK<br>HMTDRKLTMEQIAEKINAGFGDDLNCIFNDDNAEKLVLRIRIMNSDENKMQEEEEVVDKMDDDVFL<br>RCIESNMLTDMTLQGIEQISKVYMHLPQTDNKKKIIITEDGEFKALQEWILETDGVSLMRVLSEKDVDP<br>VRTTSNDIVEIFTVLGIEAVRKALERELYHVISFDGSYVNYRHLALLCDTMTCRGHLMAITRHGVNRQ<br>DTGPLMKCSFEETVDVLMEAAAHGESDPMKGVSENIMLGQLAPAGTGCFDLLLDAEKCKYGMEIPT<br>NIPGLGAAGPTGMFFGSAPSPMGGISPAMTPWNQGATPAYGAWSPSVGSGMTPGAAGFSPSAASDA<br>SGFSPGYSPAWSPTPGSPGSPGPSSPYIPSPGGAMSPSYSPTSPAYEPRSPGGYTPQSPSYSPTSPSYSPTSP<br>SYSPTSPNYSPTSPSYSPTSPSYSPTSPSYSPTSPSYSPTSPSYSPTSPSYSPTSPSYSPTSPSYSPTSPSYSPTSPS<br>YSPTSPSYSPTSPNYSPTSPNYTPTSPSYSPTSPSYSPTSPNYTPTSPNYSPTSPSYSPTSPSYSPTSPSYSPSSP<br>RYTPQSPTYTPSSPSYSPSSPSYSPTSPKYTPTSPSYSPSSPEYTPTSPKYSPTSPKYSPTSPKYSPTSPTYSPTT<br>PKYSPTSPTYSPTSPVYTPTSPKYSPTSPTYSPTSPKYSPTSPTYSPTSPKGSTYSPTSPGYSPTSPTYSLTSPAI<br>SPDDSDEEN	
27	DNA-directed RNA polymerase subunit	A0A480Z7V7	No data	The same (25)	Match to NLHLP at 502<br>MHGGGPPSGDSACPLRTIKRVQFGVLSPDELKRMSVTEGGIKYPETTEGGRPKLGGLMDPRQGVIERT<br>GRCQTCAGNMTECPGHFGHIELAKPVFHVGFLVKTMKVLRCVCFFCSKLLVDSNNPKIKDILAKSKG<br>QPKKRLTHVYDLCKGKNICEGGEEMDNKFGVEQPEGDEDLTKEKGHGGCGRYQPRIRRSGLELYAE<br>WKHVNEDSQEKKILLSPERVHEIFKRISDEECFVLGMEPRYARPEWMIVTVLPVPPLSVRPAVVMQGS<br>ARNQDDLTHKLADIVKINNQLRRNEQNGAAAHVIAEDVKLLQFHVATMVDNELPGLPRAMQKSG<br>RPLKSLKQRLKGKEGRVRGNLMGKRVDFSARTVITPDPNLSIDQVGVPRSIAANMTFAEIVTPFNIDRL<br>QELVRRGNSQYPGAKYIIRDNGDRIDLRFHPKPSDLHLQTGYKVERHMCDGDIVIFNRQPTLHKMSM<br>MGHRVRILPWSTFRLNLSVTTPYNADFDGDEMNLHLPQSLETRAEIQELAMVPRMIVTPQSNRPVMG<br>IVQDTLTAVRKFTKRDVFLERGEVMNLLMFLSTWDGKVPQPAILKPRPLWTGKQIFSLIIPGHINCIRT<br>HSTHPDDEDSGPYKHISPGDTKVVVENGELIMGILCKKSLGTSAGSLVHISYLEMGHDITRLFYSNIQT	

					VINNWLLIEGHTIGIGDSIADSKTYQDIQNTIKKAKQDVIEVIEKAHNNELEPTPGNTLRQTFENQVNR<br>ILNDARDKTGSSAQKSLSEYNNFKSMVVSGAKGSKINISQVIAVVGQQNVEGKRIPFGFKHRTLPHFIK<br>DDYGPESRGFVENSYLAGLTPTEFFFHAMGGREGLIDTAVKTAETGYIQRRLIKSMESVMVKYDATVR<br>NSINQVVQLRYGEDGLAGESVEFQNLATLKPSNKAFEKKFRFDYTNERALRRTLQEDLVKDVLSNAH<br>IQNELEREFERMREDREVLRVIFPTGDSKVVLPCNLLRMIWNAQKIFHINPRLPSDLHPIKVVEGVKEL<br>SKKLVIVNGDDPLSRQAQENATLLFNIHLRSTLCSRRMAEEFRLSGEAFDWLLGEIESKFNQAIAHPGE<br>MVGALAAQSLGEPATQMTLNTFHYAGVSAKNVTLGVPRLKELINISKKPKTPSLTVFLLGQSARDAE<br>RAKDILCRLEHTTLRKVTANTAIYYDPNPQSTVVAEDQEWVNVYYEMPDFDVARISPWLLRVELDRK<br>HMTDRKLTMEQIAEKINAGFGDDLNCIFNDDNAEKLVLRIRIMNSDENKMQEEEEVVDKMDDDVFL<br>RCIESNMLTDMTLQGIEQISKVYMHLPQTDNKKKIIITEDGEFKALQEWILETDGVSLMRVLSEKDVDP<br>VRTTSNDIVEIFTVLGIEAVRKALERELYHVISFDGSYVNYRHLALLCDTMTCRGHLMAITRHGVNRQ<br>DTGPLMKCSFEETVDVLMEAAAHGESDPMKGVSENIMLGQLAPAGTGCFDLLLDAEKCKYGMEIPT<br>NIPGLGAAGPTGMFFGSAPSPMGGISPAMTPWNQGATPAYGAWSPSVGSGMTPGAAGFSPSAASDA<br>SGFSPGYSPAWSPTPGSPGSPGPSSPYIPSPGGAMSPSYSPTSPAYEPRSPGGYTPQSPSYSPTSPSYSPTSP<br>SYSPTSPNYSPTSPSYSPTSPSYSPTSPSYSPTSPSYSPTSPSYSPTSPSYSPTSDLPQLLAHLAQLLTHFSQLL<br>AHLAQLLAHLPQLLAHLAQLLTHFSQLLANFPQLLAHLAQLLAHISQLLSDIPQLLTNFPQLLTHQP	
28	DNA-directed RNA polymerase subunit	A0A480J736	No data	The same (17)	Match to NLHLP at 502<br>MHGGGPPSGDSACPLRTIKRVQFGVLSPDELKRMSVTEGGIKYPETTEGGRPKLGGLMDPRQGVIERT<br>GRCQTCAGNMTECPGHFGHIELAKPVFHVGFLVKTMKVLRCVCFFCSKLLVDSNNPKIKDILAKSKG<br>QPKKRLTHVYDLCKGKNICEGGEEMDNKFGVEQPEGDEDLTKEKGHGGCGRYQPRIRRSGLELYAE<br>WKHVNEDSQEKKILLSPERVHEIFKRISDEECFVLGMEPRYARPEWMIVTVLPVPPLSVRPAVVMQGS<br>ARNQDDLTHKLADIVKINNQLRRNEQNGAAAHVIAEDVKLLQFHVATMVDNELPGLPRAMQKSG<br>RPLKSLKQRLKGKEGRVRGNLMGKRVDFSARTVITPDPNLSIDQVGVPRSIAANMTFAEIVTPFNIDRL<br>QELVRRGNSQYPGAKYIIRDNGDRIDLRFHPKPSDLHLQTGYKVERHMCDGDIVIFNRQPTLHKMSM<br>MGHRVRILPWSTFRLNLSVTTPYNADFDGDEMNLHLPQSLETRAEIQELAMVPRMIVTPQSNRPVMG<br>IVQDTLTAVRKFTKRDVFLERGEVMNLLMFLSTWDGKVPQPAILKPRPLWTGKQIFSLIIPGHINCIRT<br>HSTHPDDEDSGPYKHISPGDTKVVVENGELIMGILCKKSLGTSAGSLVHISYLEMGHDITRLFYSNIQT<br>VINNWLLIEGHTIGIGDSIADSKTYQDIQNTIKKAKQDVIEVIEKAHNNELEPTPGNTLRQTFENQVNR<br>ILNDARDKTGSSAQKSLSEYNNFKSMVVSGAKGSKINISQVIAVVGQQNVEGKRIPFGFKHRTLPHFIK<br>DDYGPESRGFVENSYLAGLTPTEFFFHAMGGREGLIDTAVKTAETGYIQRRLIKSMESVMVKYDATVR<br>NSINQVVQLRYGEDGLAGESVEFQNLATLKPSNKAFEKKFRFDYTNERALRRTLQEDLVKDVLSNAH<br>IQNELEREFERMREDREVLRVIFPTGDSKVVLPCNLLRMIWNAQKIFHINPRLPSDLHPIKVVEGVKEL<br>SKKLVIVNGDDPLSRQAQENATLLFNIHLRSTLCSRRMAEEFRLSGEAFDWLLGEIESKFNQAIAHPGE<br>MVGALAAQSLGEPATQMTLNTFHYAGVSAKNVTLGVPRLKELINISKKPKTPSLTVFLLGQSARDAE<br>RAKDILCRLEHTTLRKVTANTAIYYDPNPQSTVVAEDQEWVNVYYEMPDFDVARISPWLLRVELDRK<br>HMTDRKLTMEQIAEKINAEEFCEKDRKAKREPRMGCGCKRSRFSQVSVT	
29	DNA-directed RNA polymerase subunit	A0A480H8S4	No data	The same (17)	Match to NLHLP at 506<br>MVKEQFRETDVAKKISHICFGMKSAEEMRQQAHIQVVSKNLYSQDNNHSPLLYGVLDHRMGTSEKD<br>RPCETCGKNLADCLGHYGYIDLELPCFHVGYFRAVIGILQMICKTCCHIMLSQEERKQFLDYLKRPGL<br>TYLQKRGLKKKISDKCRKKNTCHHCGAFNGTVKKCGLLKIIHEKYKTNKKVVDPIVSNFLQSFETAIE<br>HNKEVEPLLGRAQENLNPLVVLNLFKRIPAEDIPLLLMNPEAGKPSDLILTRLLVPPLCIRPSVVSDLKS<br>GTNEDDLTMKLTEIIFLNDVIKKHRISGAKTQMIMEDWDFLQLQCALYINSELSGIPLNMAPKKWTRG<br>FVQRLKGKQGRFRGNLSGKRVDFSGRTVISPDPNLRIDEVAVPVHVAKILTFPEKVNKANITFLRKLVR<br>NGPEVHPGANFIQQRHTQMKRFLKYGNREKMAQELKFGDIVERHLIDGDVVLFNRQPSLHKLSIMA<br>HLARVKPHRTFRFNECVCTPYNADFDGDEMNLHLPQTEEAKAEALVLMGTKANLVTPRNGEPLIAA<br>IQDFLTGAYLLTLKDTFFDRAKACQIIASILVGKDEKIKVRLPPPTILKPVTLWTGKQVFSVILRPSYDNP<br>VRANLRTKGKQYCGKGEDLCVNDSYVTIQNSELMSGSMDKGTLGSGSKNNIFYILLRDWGQNEAAD<br>AMSRLARLAPVYLSNRGFSIGIGDVTPGQGLLKAKYELLNAGYRKCDEYIEALNTGKLQQQPGCTAE	90% with A0A480MHM1 (23)

					ETLEALILKELSVIRDHAGSACLRELDKSNSPLTMALCGSKGSFINISQMIACVGQQAISGSRVPDGFEN<br>RSLPHFEKHSKLPAAKGFVANSFYSGLTPTEFFFHTMAGREGLVDTAVKTAETGYMQRRLVKSLEDLC<br>SQYDLTVRSSTGDIIQFIYGGDGLDPAAMEGKDEPLEFKRVLDNIKAVFPCQSEPALSKNELLLTTESIM<br>KKNEFLCCQDSFLQVSSEGPCAQFDCHRGIVHQVLAGTSLSWGC	
30	DNA-directed RNA polymerase subunit	A0A480NXX2	No data	The same (17)	Match to NLHLP at 502<br>MHGGGPPSGDSACPLRTIKRVQFGVLSPDELKRMSVTEGGIKYPETTEGGRPKLGGLMDPRQGVIERT<br>GRCQTCAGNMTECPGHFGHIELAKPVFHVGFLVKTMKVLRCVCFFCSKLLVDSNNPKIKDILAKSKG<br>QPKKRLTHVYDLCKGKNICEGGEEMDNKFGVEQPEGDEDLTKEKGHGGCGRYQPRIRRSGLELYAE<br>WKHVNEDSQEKKILLSPERVHEIFKRISDEECFVLGMEPRYARPEWMIVTVLPVPPLSVRPAVVMQGS<br>ARNQDDLTHKLADIVKINNQLRRNEQNGAAAHVIAEDVKLLQFHVATMVDNELPGLPRAMQKSG<br>RPLKSLKQRLKGKEGRVRGNLMGKRVDFSARTVITPDPNLSIDQVGVPRSIAANMTFAEIVTPFNIDRL<br>QELVRRGNSQYPGAKYIIRDNGDRIDLRFHPKPSDLHLQTGYKVERHMCDGDIVIFNRQPTLHKMSM<br>MGHRVRILPWSTFRLNLSVTTPYNADFDGDEMNLHLPQSLETRAEIQELAMVPRMIVTPQSNRPVMG<br>IVQDTLTAVRKFTKRDVFLERGEVMNLLMFLSTWDGKVPQPAILKPRPLWTGKQIFSLIIPGHINCIRT<br>HSTHPDDEDSGPYKHISPGDTKVVVENGELIMGILCKKSLGTSAGSLVHISYLEMGHDITRLFYSNIQT<br>VINNWLLIEGHTIGIGDSIADSKTYQDIQNTIKKAKQDVIEVIEKAHNNELEPTPGNTLRQTFENQVNR<br>ILNDARDKTGSSAQKSLSEYNNFKSMVVSGAKGSKINISQVIAVVGQQNVEGKRIPFGFKHRTLPHFIK<br>DDYGPESRGFVENSYLAGLTPTEFFFHAMGGREGLIDTAVKTAETGYIQRRLIKSMESVMVKYDATVR<br>NSINQVVQLRYGEDGLAGESVEFQNLATLKPSNKAFEKKFRFDYTNERALRRTLQEDLVKDVLSNAH<br>IQNELEREFERMREDREVLRVIFPTGDSKVVLPCNLLRMIWNAQKIFHINPRLPSDLHPIKVVEGVKEL<br>SKKLVIVNGDDPLSRQAQENATLLFNIHLRSTLCSRRMAEEFRLSGEAFDWLLGEIESKFNQAIAHPGE<br>MVGALAAQSLGEPATQMTLNTFHYAGVSAKNVTLGVPRLKELINISKKPKTPSLTVFLLGQSARDAE<br>RAKDILCRLEHTTLRKVTANTAIYYDPNPQSTVVAEDQEWVNVYYEMPDFDVARISPWLLRVELDRK<br>HMTDRKLTMEQIAEKINAGEAGGLLSRPPPVPSLCAPPRQAIHTPPSMPWFCSRPLCTAFSFSRFPLLLS<br>RRVL	90% with A0A480J736 (28)
31	DNA-directed RNA polymerase subunit	A0A480TA59	No data	The same (25)	Match to NLHLP at 502<br>MHGGGPPSGDSACPLRTIKRVQFGVLSPDELKRMSVTEGGIKYPETTEGGRPKLGGLMDPRQGVIERT<br>GRCQTCAGNMTECPGHFGHIELAKPVFHVGFLVKTMKVLRCVCFFCSKLLVDSNNPKIKDILAKSKG<br>QPKKRLTHVYDLCKGKNICEGGEEMDNKFGVEQPEGDEDLTKEKGHGGCGRYQPRIRRSGLELYAE<br>WKHVNEDSQEKKILLSPERVHEIFKRISDEECFVLGMEPRYARPEWMIVTVLPVPPLSVRPAVVMQGS<br>ARNQDDLTHKLADIVKINNQLRRNEQNGAAAHVIAEDVKLLQFHVATMVDNELPGLPRAMQKSG<br>RPLKSLKQRLKGKEGRVRGNLMGKRVDFSARTVITPDPNLSIDQVGVPRSIAANMTFAEIVTPFNIDRL<br>QELVRRGNSQYPGAKYIIRDNGDRIDLRFHPKPSDLHLQTGYKVERHMCDGDIVIFNRQPTLHKMSM<br>MGHRVRILPWSTFRLNLSVTTPYNADFDGDEMNLHLPQSLETRAEIQELAMVPRMIVTPQSNRPVMG<br>IVQDTLTAVRKFTKRDVFLERGEVMNLLMFLSTWDGKVPQPAILKPRPLWTGKQIFSLIIPGHINCIRT<br>HSTHPDDEDSGPYKHISPGDTKVVVENGELIMGILCKKSLGTSAGSLVHISYLEMGHDITRLFYSNIQT<br>VINNWLLIEGHTIGIGDSIADSKTYQDIQNTIKKAKQDVIEVIEKAHNNELEPTPGNTLRQTFENQVNR<br>ILNDARDKTGSSAQKSLSEYNNFKSMVVSGAKGSKINISQVIAVVGQQNVEGKRIPFGFKHRTLPHFIK<br>DDYGPESRGFVENSYLAGLTPTEFFFHAMGGREGLIDTAVKTAETGYIQRRLIKSMESVMVKYDATVR<br>NSINQVVQLRYGEDGLAGESVEFQNLATLKPSNKAFEKKFRFDYTNERALRRTLQEDLVKDVLSNAH<br>IQNELEREFERMREDREVLRVIFPTGDSKVVLPCNLLRMIWNAQKIFHINPRLPSDLHPIKVVEGVKEL<br>SKKLVIVNGDDPLSRQAQENATLLFNIHLRSTLCSRRMAEEFRLSGEAFDWLLGEIESKFNQAIAHPGE<br>MVGALAAQSLGEPATQMTLNTFHYAGVSAKNVTLGVPRLKELINISKKPKTPSLTVFLLGQSARDAE<br>RAKDILCRLEHTTLRKVTANTAIYYDPNPQSTVVAEDQEWVNVYYEMPDFDVARISPWLLRVELDRK<br>HMTDRKLTMEQIAEKINAGFGDDLNCIFNDDNAEKLVLRIRIMNSDENKMQEEEEVVDKMDDDVFL<br>RCIESNMLTDMTLQGIEQISKVYMHLPQTDNKKKIIITEDGEFKALQEWILETDGVSLMRVLSEKDVDP<br>VRTTSNDIVEIFTVLGIEAVRKALERELYHVISFDGSYVNYRHLALLCDTMTCRGHLMAITRHGVNRQ<br>DTGPLMKCSFEETVDVLMEAAAHGESDPMKGVSENIMLGQLAPAGTGCFDLLLDAEKCKYGMEIPT	

					NIPGLGAAGPTGMFFGSAPSPMGGISPAMTPWNQGATPAYGAWSPSVGSGMTPGAAGFSPSAASDA<br>SGFSPGYSPAWSPTPGSPGSPGPSSPYIPSPGGAMSPSYSPTSPAYEPRSPGGYTPQSPSYSPTSPSYSPTSP<br>SYSPTSPNYSPTSPSYSPTSPSYSPTSPSYSPTSPSYSPTSPSYSPTSPSYSPTSPSYSPTSPSYSPTSPSYSPTSPS<br>YSPTSPNYSPTSPNYTPTSPSYSPTSPSYSPTSPNYTPTSPNYSPTSPSYSPTSPSYSPTSPSYSPSSPRYTPQSP<br>TYTPSSPSYSPSSPSYSPTSPKYTPTSPSYSPSSPEYTPTSPKYSPTSPKYSPTSPKYSPTSPTYSPTTPKYSPTSP<br>TYSPTSPVYTPTSPKYSPTSPTYSPTSPKYSPTSPTYSPTSPKGSTYSPTSPGYSPTSPTYSLTSPAISPDDSDE<br>EN	
32	DNA-directed RNA polymerase subunit	A0A480UVG4	No data	The same (25)	Match to NLHLP at 502<br>MHGGGPPSGDSACPLRTIKRVQFGVLSPDELKRMSVTEGGIKYPETTEGGRPKLGGLMDPRQGVIERT<br>GRCQAWAGNMTECPGHFGHIELAKPVFHVGFLVKTMKVLRCVCFFCSKLLVDSNNPKIKDILAKSK<br>GQPKKRLTHVYDLCKGKNICEGGEEMDNKFGVEQPEGDEDLTKEKGHGGCGRYQPRIRRSGLELYA<br>EWKHVNEDSQEKKILLSPERVHEIFKRISDEECFVLGMEPRYARPEWMIVTVLPVPPLSVRPAVVMQG<br>SARNQDDLTHKLADIVKINNQLRRNEQNGAAAHVIAEDVKLLQFHVATMVDNELPGLPRAMQKSG<br>RPLKSLKQRLKGKEGRVRGNLMGKRVDFSARTVITPDPNLSIDQVGVPRSIAANMTFAEIVTPFNIDRL<br>QELVRRGNSQYPGAKYIIRDNGDRIDLRFHPKPSDLHLQTGYKVERHMCDGDIVIFNRQPTLHKMSM<br>MGHRVRILPWSTFRLNLSVTTPYNADFDGDEMNLHLPQSLETRAEIQELAMVPRMIVTPQSNRPVMG<br>IVQDTLTAVRKFTKRDVFLERGEVMNLLMFLSTWDGKVPQPAILKPRPLWTGKQIFSLIIPGHINCIRT<br>HSTHPDDEDSGPYKHISPGDTKVVVENGELIMGILCKKSLGTSAGSLVHISYLEMGHDITRLFYSNIQT<br>VINNWLLIEGHTIGIGDSIADSKTYQDIQNTIKKAKQDVIEVIEKAHNNELEPTPGNTLRQTFENQVNR<br>ILNDARDKTGSSAQKSLSEYNNFKSMVVSGAKGSKINISQVIAVVGQQNVEGKRIPFGFKHRTLPHFIK<br>DDYGPESRGFVENSYLAGLTPTEFFFHAMGGREGLIDTAVKTAETGYIQRRLIKSMESVMVKYDATVR<br>NSINQVVQLRYGEDGLAGESVEFQNLATLKPSNKAFEKKFRFDYTNERALRRTLQEDLVKDVLSNAH<br>IQNELEREFERMREDREVLRVIFPTGDSKVVLPCNLLRMIWNAQKIFHINPRLPSDLHPIKVVEGVKEL<br>SKKLVIVNGDDPLSRQAQENATLLFNIHLRSTLCSRRMAEEFRLSGEAFDWLLGEIESKFNQAIAHPGE<br>MVGALAAQSLGEPATQMTLNTFHYAGVSAKNVTLGVPRLKELINISKKPKTPSLTVFLLGQSARDAE<br>RAKDILCRLEHTTLRKVTANTAIYYDPNPQSTVVAEDQEWVNVYYEMPDFDVARISPWLLRVELDRK<br>HMTDRKLTMEQIAEKINAGFGDDLNCIFNDDNAEKLVLRIRIMNSDENKMQEEEEVVDKMDDDVFL<br>RCIESNMLTDMTLQGIEQISKVYMHLPQTDNKKKIIITEDGEFKALQEWILETDGVSLMRVLSEKDVDP<br>VRTTSNDIVEIFTVLGIEAVRKALERELYHVISFDGSYVNYRHLALLCDTMTCRGHLMAITRHGVNRQ<br>DTGPLMKCSFEETVDVLMEAAAHGESDPMKGVSENIMLGQLAPAGTGCFDLLLDAEKCKYGMEIPT<br>NIPGLGAAGPTGMFFGSAPSPMGGISPAMTPWNQGATPAYGAWSPSVGSGMTPGAAGFSPSAASDA<br>SGFSPGYSPAWSPTPGSPGSPGPSSPYIPSPGGAMSPSYSPTSPAYEPRSPGGYTPQSPSYSPTSPSYSPTSP<br>SYSPTSPNYSPTSPSYSPTSPSYSPTSPSYSPTSPSYSPTSPSYSPTSPSYSPTSPSYSPTSPSYSPTSPSYSPTSPS<br>YSPTSPSYSPTSPNYSPTSPNYTPTSPSYSPTSPSYSPTSPNYTPTSPNYSPTSPSYSPTSPSYSPTSPSYSPSSP<br>RYTPQSPTYTPSSPSYSPSSPSYSPTSPKYTPTSPSYSPSSPEYTPTSPKYSPTSPKYSPTSPKYSPTSPTYSPTT<br>PKYSPTSPTYSPTSPVYTPTSPKYSPTSPTYSPTSPKYSPTSPTYSPTSPKGSTYSPTSPGYSPTSPTYSLTSPAI<br>SPDDSDEEN	
33	DNA-directed RNA polymerase subunit	A0A5G2RA15	No data	The same (17)	Match to NLHLP at 502<br>MHGGGPPSGDSACPLRTIKRVQFGVLSPDELKRMSVTEGGIKYPETTEGGRPKLGGLMDPRQGVIERT<br>GRCQTCAGNMTECPGHFGHIELAKPVFHVGFLVKTMKVLRCVCFFCSKLLVDSNNPKIKDILAKSKG<br>QPKKRLTHVYDLCKGKNICEGGEEMDNKFGVEQPEGDEDLTKEKGHGGCGRYQPRIRRSGLELYAE<br>WKHVNEDSQEKKILLSPERVHEIFKRISDEECFVLGMEPRYARPEWMIVTVLPVPPLSVRPAVVMQGS<br>ARNQDDLTHKLADIVKINNQLRRNEQNGAAAHVIAEDVKLLQFHVATMVDNELPGLPRAMQKSG<br>RPLKSLKQRLKGKEGRVRGNLMGKRVDFSARTVITPDPNLSIDQVGVPRSIAANMTFAEIVTPFNIDRL<br>QELVRRGNSQYPGAKYIIRDNGDRIDLRFHPKPSDLHLQTGYKVERHMCDGDIVIFNRQPTLHKMSM<br>MGHRVRILPWSTFRLNLSVTTPYNADFDGDEMNLHLPQSLETRAEIQELAMVPRMIVPQSNRPVMGI<br>VQDTLTAVRKFTKRDVFLERVCGPTGDLAW	90% with A0A4X1V3A0 (21)

34	DNA-directed RNA polymerase subunit	A0A4X1V400	No data	The same (25)	Match to NLHLP at 502<br>MHGGGPPSGDSACPLRTIKRVQFGVLSPDELKRMSVTEGGIKYPETTEGGRPKLGGLMDPRQGVIERT<br>GRCQTCAGNMTECPGHFGHIELAKPVFHVGFLVKTMKVLRCVCFFCSKLLVDSNNPKIKDILAKSKG<br>QPKKRLTHVYDLCKGKNICEGGEEMDNKFGVEQPEGDEDLTKEKGHGGCGRYQPRIRRSGLELYAE<br>WKHVNEDSQEKKILLSPERVHEIFKRISDEECFVLGMEPRYARPEWMIVTVLPVPPLSVRPAVVMQGS<br>ARNQDDLTHKLADIVKINNQLRRNEQNGAAAHVIAEDVKLLQFHVATMVDNELPGLPRAMQKSG<br>RPLKSLKQRLKGKEGRVRGNLMGKRVDFSARTVITPDPNLSIDQVGVPRSIAANMTFAEIVTPFNIDRL<br>QELVRRGNSQYPGAKYIIRDNGDRIDLRFHPKPSDLHLQTGYKVERHMCDGDIVIFNRQPTLHKMSM<br>MGHRVRILPWSTFRLNLSVTTPYNADFDGDEMNLHLPQSLETRAEIQELAMVPRMIEQSPVMGIVQD<br>TLTAVRKFTKRDVFLERGEVMNLLMFLSTWDGKVPQPAILKPRPLWTGKQIFSLIIPGHINCIRTHSTH<br>PDDEDSGPYKHISPGDTKVVVENGELIMGILCKKSLGTSAGSLVHISYLEMGHDITRLFYSNIQTVINN<br>WLLIEGHTIGIGDSIADSKTYQDIQNTIKKAKQDVIEVIEKAHNNELEPTPGNTLRQTFENQVNRILND<br>ARDKTGSSAQKSLSEYNNFKSMVVSGAKGSKINISQVIAVVGQQNVEGKRIPFGFKHRTLPHFIKDDY<br>GPESRGFVENSYLAGLTPTEFFFHAMGGREGLIDTAVKTAETGYIQRLIKSMESVMVKYDATVRNSIN<br>QVVQLRYGEDGLGESVEFQNLATLKPSNKAFEKKFRFDYTNERALRRTLQEDLVKDVLSNAHIQNEL<br>ERFERMREDREVLRVIFPTGDSKVVLPCNLLRMIWNAQKIFHINPRLPSDLHPIKVVEGVKELSKKLVI<br>VNGDDPLSRQAQENATLLFNIHLRSTLCSRRMAEEFRLSGEAFDWLLGEIESKFNQAIAHPGEMVGA<br>LAAQSLGEPATQMTLNTFHYAGVSAKNVTLGVPRLKELINISKKPKTPSLTVFLLGQSARDAERAKDI<br>LCRLEHTTLRKVTANTAIYYDPNPQSTVVAEDQEWVNVYYEMPDFDVARIPWLLRVELDRKHMTDR<br>KLTMEQIAEKINAGFGDDLNCIFNDDNAEKLVLRIRIMNSDENKMQEEEEVVDKMDDDVFLRCIESN<br>MLTDMTLQVYMHLPQTDNKKKIIITEDGEFKALQEWILETDGVSLMRVLSEKDVDPVRTTSNDIVEIF<br>TVLGIEAVRKALERELYHVISFDGSYVNYRHLALLCDTMTCRGHLMAITRHGVNRQDTGPLMKCSFE<br>ETVDVLMEAAAHGESDPMKGVSENIMLGQLAPAGTGCFDLLLDAEKCKYGMEIPTNIPGLGAAGPT<br>GMFFGSAPSPMGGISPAMTPWNQGATPAYGAWSPSVGSGMTPGAAGFSPSAASDASGFSPGYSPAW<br>SPTPGSPGSPGPSSPYIPSPGGAMSPSYSPTSPAYEPRSPGGYTPQSPSYSPTPSYSPTSPSYSPTSPNYSPTS<br>PSYSPTSPSYSPTSPSYSPTSPYSPTSPSYSPTSPSYSPTSPSYSPTSPSYSPTSPYSPTSPSYSPTSPSYSPTSPSYS<br>PTSPSYSPTSPSYSPTSPSYSPTSPNYSPTSPNYTPTSPSYSPTSPSYSPTSPNYTPTPNYSPTSPSYSPTSPSYS<br>PTSPSYSPSSPRYTPQSPTYTPSSPSYSPSSPSYSPTSPKYTPTSPSYSPSSPEYTPTSPKYSPTSPKYSPTSPKYS<br>PTSPTYSPTTPKYSPTSPTYSPTSPVYTPTPKYSPTSPTYSPTSPKYSPTSPTYSPTSPKGSTYSPTSPGYSPTS<br>PTYSLTSPAISPDDSDEEN	
35	DNA-directed RNA polymerase subunit	A0A7M4DUC2	No data	No data	Match to NLHLP at 502<br>MHGGGPPSGDSACPLRTIKRVQFGVLSPDELKRMSVTEGGIKYPETTEGGRPKLGGLMDPRQGVIERT<br>GRCQTCAGNMTECPGHFGHIELAKPVFHVGFLVKTMKVLRCVCFFCSKLLVDSNNPKIKDILAKSKG<br>QPKKRLTHVYDLCKGKNICEGGEEMDNKFGVEQPEGDEDLTKEKGHGGCGRYQPRIRRSGLELYAE<br>WKHVNEDSQEKKILLSPERVHEIFKRISDEECFVLGMEPRYARPEWMIVTVLPVPPLSVRPAVVMQGS<br>ARNQDDLTHKLADIVKINNQLRRNEQNGAAAHVIAEDVKLLQFHVATMVDNELPGLPRAMQKSG<br>RPLKSLKQRLKGKEGRVRGNLMGKRVDFSARTVITPDPNLSIDQVGVPRSIAANMTFAEIVTPFNIDRL<br>QELVRRGNSQYPGAKYIIRDNGDRIDLRFHPKPSDLHLQTGYKVERHMCDGDIVIFNRQPTLHKMSM<br>MGHRVRILPWSTFRLNLSVTTPYNADFDGDEMNLHLPQSLETRAEIQELAMVPRMIVTPQSNRPVMG<br>IVQDTLTAVRKFTKRDVFLERGEVMNLLMFLSTWDGKVPQPAILKPRPLWTGKQIFSLIIPGHINCIRT<br>HSTHPDDEDSGPYKHISPGDTKVVVENGELIMGILCKKSLGTSAGSLVHISYLEMGHDITRLFYSNIQT<br>VINNWLLIEGHTIGIGDSIADSKTYQDIQNTIKKAKQDVIEVIEKAHNNELEPTPGNTLRQTFENQVNR<br>ILNDARDKTGSSAQKSLSEYNNFKSMVVSGAKGSKINISQVIAVVGQQNVEGKRIPFGFKHRTLPHFIK<br>DDYGPESRGFVENSYLAGLTPTEFFFHAMGGREGLIDTAVKTAETGYIQRRLIKSMESVMVKYDATVR<br>NSINQVVQLRYGEDGLAGESVEFQNLATLKPSNKAFEKKFRFDYTNERALRRTLQEDLVKDVLSNAH<br>IQNELEREFERMREDREVLRVIFPTGDSKVVLPCNLLRMIWNAQKIFHINPRLPSDLHPIKVVEGVKEL<br>SKKLVIVNGDDPLSRQAQENATLLFNIHLRSTLCSRRMAEEFRLSGEAFDWLLGEIESKFNQAIAHPGE<br>MVGALAAQSLGEPATQMTLNTFHYAGVSAKNVTLGVPRLKELINISKKPKTPSLTVFLLGQSARDAE	

					RAKDILCRLEHTTLRKVTANTAIYYDPNPQSTVVAEDQEWVNVYYEMPDFDVARISPWLLRVELDRK<br>HMTDRKLTMEQIAEKINAGFGDDLNCIFNDDNAEKLVLRIRIMNSDENKMQEEEEVVDKMDDDVFL<br>RCIESNMLTDMTLQGIEQISKVYMHLPQTDNKKKIIITEDGEFKALQEWILETDGVSLMRVLSEKDVDP<br>VRTTSNDIVEIFTVLGIEAVRKALERELYHVISFDGSYVNYRHLALLCDTMTCRGHLMAITRHGVNRQ<br>DTGPLMKCSFEETVDVLMEAAAHGESDPMKGVSENIMLGQLAPAGTGCFDLLLDAEKCKYGMEIPT<br>NIPGLGAAGPTGMFFGSAPSPMGGISPAMTPWNQGATPAYGAWSPSVGSGMTPGAAGFSPSAASDA<br>SGFSPGYSPAWSPTPGSPGSPGPSSPYIPSPGGAMSPSYSPTSPAYEPRSPGGYTPQSPSYSPTSPSYSPTSP<br>SYSPTSPNYSPTSPSYSPTSPSYSPTSPSYSPTSPSYSPTSPSYSPTSPSYSPTSPSYSPTSPSYSPTSPSYSPTSPS<br>YSPTSPSYSPTSPSYSPTSPSYSPTSPSYSPTSPSYSPTSPSYSPTSPSYSPTSPNYSPTSPNYTPTSPSYSPTSPS<br>YSPTSPNYTPTSPNYSPTSPSYSPTSPSYSPTSPSYSPSSPRYTPQSPTYTPSSPSYSPSSPSYSPTSPKYTPTSPS<br>YSPSSPEYTPTSPKYSPTSPKYSPTSPKYSPTSPTYSPTTPKYSPTSPTYSPTSPVYTPTSPKYSPTSPTYSPTSP<br>KYSPTSPTYSPTSPKGSTYSPTSPGYSPTSPTYSLTSPAISPDDSDEEN	
36	Testis associated actin 29emodeling kinase 2	A0A287AGE1	Low in heart left ventricle	GO:0004672<br>GO:0005524<br>GO:0006468	Match to NLHLP at 146<br>MDRSKRNSIAGFPPRVERLEDFEGGGGGGDGSLSHVGRVWPSSYRALISAFSRLTRLDDFTCEKIGSGF<br>FSEVFKVRHRASGQVMALKMNTLSSNRANMLKEVQLMNRLSHPNILRFMGVCVHQGQLHALTEYI<br>NSGNLEQLLDSNLHLPWTVRVKLAYDIAVGLSYLHFKGIFHRDLTSKNCLIKRDESGYSAVVADFGLA<br>EKIPDGSMGSEKLAVVGSPFWMAPEVLRDEPYNEKADVFSYGIILCEIIARIQADPDYLPRTEMDPKLR<br>PSFAEIGKTLEEILSRLQEEELERDRKLQPAAKGLLEKGPGLKRLSLQDNKIPPKSPRPRRTIWLSRSQSD<br>IFSSKPPRTVNVLDPYYQPQRGGAARAPKINPFSARQDLKGGKIKFFDLPSKSVISLVFDLDAPGPGSTP<br>VAEWQEPLAPPARRWRSLPGSPEFLHQEACPFVGREESLSDGPPPRLSSHKYRVREIPPFRAPALPAAP<br>AHEAMDCSGPQEENGFGPRPPGPGLCLAGALEEMEVEERPRDLAVSGTGLKTQGEQDG	90 % with A0A4X1WBG5 (15)
37	Dual specificity testis-specific protein kinase 2 isoform X1	F1S370	Low in heart (49.92), heart left ventricle (48.40) and endocardial endothelium (43.73)	The same (15,35) +<br>GO:0004674<br>GO:0030036	Match to NLHLP at 146<br>MDRSKRNSIAGFPPRVERLEDFEGGGGGGDGSLSHVGRVWPSSYRALISAFSRLTRLDDFTCEKIGSGF<br>FSEVFKVRHRASGQVMALKMNTLSSNRANMLKEVQLMNRLSHPNILRFMGVCVHQGQLHALTEYI<br>NSGNLEQLLDSNLHLPWTVRVKLAYDIAVGLSYLHFKGIFHRDLTSKNCLIKRDESGYSAVVADFGLA<br>EKIPDGSMGSEKLAVVGSPFWMAPEVLRDEPYNEKADVFSYGIILCEIIARIQADPDYLPRTENFGLDY<br>DAFQHMVGDCPPDFLQLTFNCCNMDPKLRPSFAEIGKTLEEILSRLQEEELERDRKLQPAAKGLLEKG<br>PGLKRLSLQDNKIPPKSPRPRRTIWLSRSQSDIFSSKPPRTVNVLDPYYQPQRGGAARAPKINPFSARQD<br>LKGGKIKFFDLPSKSVISLVFDLDAPGPGSTPVAEWQEPLAPPARRWRSLPGSPEFLHQEACPFVGREE<br>SLSDGPPPRLSSHKYRVREIPPFRAPALPAAPAHEAMDCSGPQEENGFGPRPPGPGLCLAGALEEMEV<br>EERPRDLAVSGTGLKTQGEQDG	90 % with A0A4X1WBG5 (15) and A0A287AGE1 (35)
38	NCK-interacting protein with SH3 domain isoform 2	A0A480QVB2	No data	GO:0005515	Match to NLHLP at 554<br>MYRALYAFRSAEPNALAFAAGETFLVLERSSAHWWLAARARSGETGYVPPAYLRRLQGLEQDVLQA<br>IDRAIEAVHNAAMRDGGKYSLEQRGVLQKLIHHRKETLSRRGPSAPSPAAMTSSTSDHHLDTAAARQ<br>PNGVCRAGFERQHSLPSSEYLGADGGLYQIPPQPRRAAPTTPPPPVKRRDREALVASGSGGCNTTPSG<br>GSSVSSGSSVSSTSLDTYYTGSSSSELGPSCSPTPPPVPRRSTHTTVSQAQPPPSKVPNPEPPAEEVAVDTA<br>SAPDELEALGTLSLGTTEEKAAAETAVPRTIGAELMELVRRNTGLSHELCRVAIGVVVGHIQASVPASS<br>PVMEQVLLSLVEGKDLSTALPSGQVCHDQQRLEVIFADLARRKDDAQQRSWALYEDEGVIRCYLEEL<br>LHILTDADPEVCKKMCKRNEFESVLALVAYYQMEHRVSLRLLLLKCFGAMCSLDAAIISTLVSSVLPV<br>ELARDMQTDTQDHQKLCYSALILAMIFSMGEAVPYAHYEHLGTPFAQFLLSIVEDGLPLDTTEQLPDL<br>CMNLLLALNLHLPAPDQNIIMAALSKHANVKIFSEKLLLLLNRGDDPVRIFKHEPQPPHSILKFLQDV<br>FASPATAAIFYHTDMMALIDITVRHIADLSPGDKLRMEYLSLMHAVVRSTPYLQHRHRLPDLQATLR<br>RILTEEEASPQCQMDRMIVREMCKEFPVLGEAPS	
39	NCK interacting protein with SH3 domain	F1SKK3	Medium-low in heart (52.36) and	GO:0010976	Match to NLHLP at 555<br>MSRGKASAARRSWPGVLNPQTSPSLLPRPAPAQPGPSPCTARCTRSARRSPTRWRSPRARPSWCWSA<br>AARTGGWRRGRAVARRATCRPPTCAACRKLIHHRKETLSRRGPSAPSPAAMTSSTSDHHLDTAAAR<br>QPNGVCRAGFERQHSLPSSEYLGADGGLYQIPPQPRRAAPTTPPPPVKRRDREALVASGSGGCNTTPS<br>GGSSVSSGSSVSSTSLDTYYTGSSSSELGPSCSPTPPPVPRRSTHTTVSQAQPPPSKVPNPEPPAEEVAVDT	

			endocardial endothelium (47.02)		ASAPDELEALGTLSLGTTEEKAAAETAVPRTIGAELMELVRRNTGLSHELCRVAIGVVVGHIQASVPASSPVMEQVLLSLVEGKDLSTALPSGQVCHDQQRLEVIFADLARRKDDAQQRSWALYEDEGVIRCYLEELLHILTDADPEVCKKMCKRNEFESVLALVAYYQMEHRVSLRLLLLKCFGAMCSLDAAIISTLVSSVLPVELARDMQTDTQDHQKLCYSALILAMIFSMGEAVPYAHYEHLGTPFAQFLLSIVEDGLPLDTTEQLPDLCMNLLLALNLHLPAPDQNIIMAALSKHANVKIFSEKLLLLLNRGDDPVRIFKHEPQPPHSILKFLQDVFASPATAAIFYHTDMMALIDITVRHIADLSPGDKLRMEYLSLMHAVVRSTPYLQHRHRLPDLQATLRRILTEEEASPQCQMDRMIVREMCKEFPVLGEAPS	
40	NCK interacting protein with SH3 domain	A0A5G2RIX3	No data	The same (38)	Match to NLHLP at 553<br>MYRALYAFRSAEPNALAFAAGETFLVLERSSAHWWLAARARSGETGYVPPAYLRRLQGLEQDVLQAIDRAIEAVHNAAMRDGGKYSLEQRGVLQKLIHHRKETLSRRGPSAPSPAAMTSSTSDHHLDTAAARQPNGVCRAGFERQHSLPSSEYLGADGGLYQIPPQPRRAAPTTPPPPVKRRDREALVASGSGGCNTTPSGGSSVSSGSSVSSTSLDTYYTGSSSSELGPSCSPTPPPVPRRSTHTTVSQAQPPPSKVPNPEPPAEEVAVDTASAPDELEALGTLSLGTTEEKAAAETAVPRTIGAELMELVRRNTGLSHELCRVAIGVVVGHIQASVPASSPVMEQVLLSLVEGKDLSTALPSGQVCHDQQRLEVIFADLARRKDDAQQRSWALYEDEGVIRCYLEELLHILTDADPEVCKKMCKRNEFESVLALVAYYQMHRVSLRLLLLKCFGAMCSLDAAIISTLVSSVLPVELARDMQTDTQDHQKLCYSALILAMIFSMGEAVPYAHYEHLGTPFAQFLLSIVEDGLPLDTTEQLPDLCMNLLLALNLHLPAPDQNIIMAALSKHANVKIFSEKLLLLLNRGDDPVRIFKHEPQPPHSILKFLQDVFASPATAAIFYHTDMMALIDITVRHIADLSPGDKLRMEYLSLMHAVVRSTPYLQHRHRLPDLQATLRRILTEEEASPQCQMDRMIVREMCKEFPVLGEAPS	90% with A0A480QVB2 (38)
41	SH3 domain-containing protein	A0A4X1T134	No data	The same (38)	Match to NLHLP at 559<br>MYRALYAFRSAEPNALAFAAGETFLVLERSSAHWWLAARARSGETGYVPPAYLRRLQGLEQDVLQAIDRAIEAVHNAAMRDGGKYSLEQRGVLQKLIHHRKETLSRRGPSAPSPAAMTSSTSDHHLDTAAARQPNGVCRAGFERQHSLPSSEYLGADGGLYQIPLLSPIPPQPRRAAPTTPPPPVKRRDREALVASGSGGCNTTPSGGSSVSSGSSVSSTSLDTFYTGSSSSELGPSCSPTPPPVPRRSTHTTVSQAQPPPSKVPNPEPPAEEVAVDTASAPDELEALGALSLGTTEEKAAAETAVRTIGAELMELVRRNTGLSHELCRVAIGVVVGHIQASVPASSPVMEQVLLSLVEGKDLSTALPSGQVCHDQQRLEVIFADLARRKDDAQQRSWALYEDEGVIRCYLEELLHILTDADPEVCKKMCKRNEFESVLALVAYYQMEHRVSLRLLLLKCFGAMCSLDAAIISTLVSSVLPVELARDMQTDTQDHQKLCYSALILAMIFSMGEAVPYAHYEHLGTPFAQFLLSIVEDGLPLDTTEQLPDLCMNLLLALNLHLPAPDQNIIMAALSKHANVKIFSEKLLLLLNRGDDPVRIFKHEPQPPHSILKFLQDVFASPATAAIFYHTDMMALIDITVRHIADLSPGDKLRMEYLSLMHAVVRSTPYLQHRHRLPDLQATLRRILTEEEASPQCQMDRMIVREMCKEFPVLGEAPS	
42	SH3 domain-containing protein	A0A4X1SYU9	No data	The same (38)	Match to NLHLP at 553<br>MYRALYAFRSAEPNALAFAAGETFLVLERSSAHWWLAARARSGETGYVPPAYLRRLQGLEQDVLQAIDRAIEAVHNAAMRDGGKYSLEQRGVLQKLIHHRKETLSRRGPSAPSPAAMTSSTSDHHLDTAAARQPNGVCRAGFERQHSLPSSEYLGADGGLYQIPPQPRRAAPTTPPPPVKRRDREALVASGSGGCNTTPSGGSSVSSGSSVSSTSLDTFYTGSSSSELGPSCSPTPPPVPRRSTHTTVSQAQPPPSKVPNPEPPAEEVAVDTASAPDELEALGALSLGTTEEKAAAETAVRTIGAELMELVRRNTGLSHELCRVAIGVVVGHIQASVPASSPVMEQVLLSLVEGKDLSTALPSGQVCHDQQRLEVIFADLARRKDDAQQRSWALYEDEGVIRCYLEELLHILTDADPEVCKKMCKRNEFESVLALVAYYQMEHRVSLRLLLLKCFGAMCSLDAAIISTLVSSVLPVELARDMQTDTQDHQKLCYSALILAMIFSMGEAVPYAHYEHLGTPFAQFLLSIVEDGLPLDTTEQLPDLCMNLLLALNLHLPAPDQNIIMAALSKHANVKIFSEKLLLLLNRGDDPVRIFKHEPQPPHSILKFLQDVFASPATAAIFYHTDMMALIDITVRHIADLSPGDKLRMEYLSLMHAVVRSTPYLQHRHRLPDLQATLRRILTEEEASPQCQMDRMIVREMCKEFPVLGEAPS	90% with A0A480QVB2 (38) and A0A5G2RIX3 (40)
43	GB1/RHD3-type G domain-containing protein	A0A4X1UA25	No data	GO:0005525<br>GO:0003924	Match to NLHLP at 340<br>MASGVHMPEPQCLIENINGRLAVNPKALKLLSAIKQPLVVVAIVGLYRTGKSYLMNKLAGKNKGFSVGSTVQSHTKGIWMWCVPHPRKPDHTLVLLDTEGLGDVEKGDKKNDTQIFVLALLLSSTFVYNTMNKIDQRAIDLLHYVAELATWLQTVSSTDADEVSGPEDSVSNCPDLVWTLRDFFLDLEVNGHPITTDEYLENSLRPKPGADKSLQNFNLPRQCIQKFFPTKKCFIFDSPTHRKKLAQLETLHDDDLEPDFVQQVAEFCS	

					YIFSHSKSKTLPEGSKANGSHLERVVLTYVKAISSGDLPCVENTVLALAQVKNSAAMKTAIAHYDQL<br>MGQNLHLPTETLQELLDLHRICKKVAIEVFVMNSFKDVDHGFQKKLETLLEAKQNELHERNLKTSLD<br>RCSSLLQVIFEPLEEEVKQGFYSIPGGHRLFMQRREELKAVYYQVPWKGLQAEEALRKYLQSKESMNV<br>TIFQTDLALTQREKEMEEARLQAEAVNFKVQVLAAILTQQHQMMEQRQRFYQEQVRRMEINRLHQ<br>QVLQQRAQERYLQEEAKRIQERAQAENKRLQDELEHLQINDSNDDKCIIL	
44	Guanylate-binding protein 5	A0A480TKG6	No data	The same (43)	Match to NLHLP at 261<br>MWCVPHPRKPDHTLVLLDTEGLGDVEKGDKKNDTQIFVLALLLSSTFVYNTMNKIDQRAIDLLHYV<br>AELATWLQTVSSTDADEVSGPEDSVSNCPDLVWTLRDFFLDLEVNGHPITTDEYLENSLRPKPGADKS<br>LQNFNLPRQCIQKFFPTKKCFIFDSPTHRKKLAQLETLHDDDLEPDFVQQVAEFCSYIFSHSKSKTLPEG<br>SKANGSHLERVVLTYVKAISSGDLPCVENTVLALAQVKNSAAMKTAIAHYDQLMGQNLHLPTETLQ<br>ELLDLHRICKKVAIEVFVMNSFKDVDHGFQKKLETLLEAKQNELHERNLKTSLDRCSSLLQVIFEPLEE<br>EVKQGFYSIPGGHRLFMQRREELKAVYYQVPWKGLQVHLVYALIRGHCWGSSSRNQ	
45	Neurobeachin-like protein 1 isoform X1	A0A480E999	No data	GO:0005515	Match to NLHLP at 1685<br>MASRERLFELWMLYCAKKDPDYLKLWLDNFVSSYEQFLDVDFEKLPTRVDDVPPGISLLPDNILQVLR<br>SQLLQCVQKMADGLEEEQQALSILLVKFFIILCRNLSNVEEIGTCSYINHVITMTTLYIQQLKSKKKEKE<br>LADQTSIEEFVIHALAFCESLYDPYRNWRHRISGRILSTVEKSRQKYKPASLTVEFVPFFYQCFQESEHL<br>KESLKCCLLHLFGAIVAGGQKNALQAISPATMEVLMRVLADCDSWEDGNPEEVGRKVELTLKCLTE<br>VVHILLTSSSDQRQVETSSILENYFKLLNSDHSALPNQRRSRQWESRFIALQIKMLNTITAMLDCTDRP<br>VLQAIFLNSNCFEHLIRLLQNCKLFLNANNKVADKNEKDLANKLLTEMNEDQVFQGQLDCLAVSAI<br>QALTAVMNKSPAAKEVFKERIGYTHMFEVLKSLGQPPLELLKELMNMAVEGDHTSVGILGISNVQPL<br>LLLIQWLPEIESHDLQIFISDWLKRICCINRQSRTTCVNANMGIRIIETLDSHSSLHRTCAENLIALHGSL<br>GSQSVSSEEIRQLLRLLRVDEPEYIHPYITPVTRAILTMARKQSLESALQYFNLSHSMAGISVPSIQKWPG<br>SAFSFNAWFCLDQDQLTLGSANKGGKRKQLYSFFTGSGMGFEAFITHSGTLVVAVCTKREYATVMLP<br>DHSFCDSLWHNITIVHMPGKRPFGQSLVYIYDNGQQKVYAPLRFPAMNEPFISCCIGSAGQRTTTPPPS<br>QIPDPPFSSPITPHRTSFGGILSSASWGGTVEKSKLITKLISAGTQDSEWGCPTSLEGQLGSVIIFSEALQPP<br>QVKALYLAGPNCLSPWKFQESDMADLPANVLLHYTAKACKNSICLDLSTNCLHGRLTGNKVVNWD<br>IKDIINCIGGLNVLFPLMEQIPLLEFGEGQIPEGPRENTVSELITPVEGDWVVLTSTKASESRLERNLIATF<br>ILIVKHFIQRHPINQDNLIHSHGVAVLGALLQKVPSTLMDVNVLMAIQLLIEQVSLEKNMQLLQQMY<br>QYLLFDFRIWNRGDFPFRIGHIQYLSTIIKDSRRVFRKKYGVQFLLDTLRIYYGSDCKYNELSLDDIRTIR<br>TSLYGLIKYFLCKGGTHEEIQSIVGYIAAISEEEQLIGILDILFSLLHTSPTRGQLFLLLFEPGNADILYALL<br>LNQKYSDRLREIIFKVMEQMLKCTNVYERSKQRIRLREVGYSGLGLLLNEAPVNTSLIKSLTNQIINTDP<br>AINFKDLLSVVYISHRACVNVRVVICRKILQTLQSQPDAAHQISQQVGWQDTLVRLFLKANFENGNT<br>PHKHIRTILMKDSDKNIATEDIKRSFDEKTDEEKISSFASAHVSSDQWSLEDRHSLDSNTPLFQEDSSVG<br>ELSFKSENQEEFWHSNPSHLSLDLSGIDSYELSDSGSQMPDSLPSTPSPIESAKSFSVQSNKESSVTNDMG<br>FSDDFTLLESQERCEEELLQLLTTILNYVMCKGLEKSDDDTWIERGQVFSALTKPGISSELLHPPDEIKLI<br>LLQKMLEWAVTENREAKINPVTAENALRLMLIIQDFLQSEGLVNSNMWTEKLLEDMMLLFDSLSVW<br>YSESPVWVKLSQIQIQLLLGFIGRGNLQVCAMASAKLNTLLQTKVIENQDEACYILGKLEHVLRQSIKE<br>QTEIYSFLIPLVRTLVSKIYELLFMNLHLPSLPFANGSSSFFEDFQEYCSSNEWQVYIEKYIVPYMKQYET<br>HTFYDGHESMALYWKDCYEALMVNMHKRDREGGESKLKFQEFFVEPFNRKARQENLRYNNMLKQ<br>LSSQQLATLRRWKAIKLYLTCERGPWAERKQKPIHWKLANVENYSRMRLKLVPNYNFKTHDEASAL<br>RDNLGIQHSQPSSDSLLLEVVKQVKVSDMDEDKLDLPEEEITTRVNIDEKEEQDQKEKLVLSEDCELITI<br>IDVIPGRLEITTQHIYFYDGSIEKEDGVGFDFKWPHSQVREIHLRRYNLRRSALEIFHVDQSNYFLNFKK<br>EVRNKVYSRLLSLHSPNSYGTRSPQELFKTSGLTQKWVNREISNFDYLIQLNTMAGRTYNDLAQYPVF<br>PWILQDYTSEELDLNNPSVFRDLSKPIGVVNDKNAKAMREKYENFEDPMGTIDKFHYGTHYSNSAGV<br>MHYLIRTEPFTTLHIQLQSGRFDCADRQFHSIPATWQALMDNPYDVKELIPEFFYFPEFLENQNKFNL<br>GRLQVSKEVVNDVILPKWAKSAEDFIYKHRKALESEYVSAHLHEWIDLIFGYKQRGPAAVEALNVFY<br>YCSYEGAVDLDALTDEKERKALEGMINNFGQTPCQLLKEPHPSRLSAEEAVQKQTKTDTSTLNLFQH<br>LPELKSFFIEGISDGIPLVKAIVPKNQSRSFMSQGSPELLITVSMNYVIGTHGWLPYDRTISNYFTFIKDQT	

					VTNPKTQRVMNGPFAPGLEITSKLFIVSHDAKLLFSAGHWDNSIQVMSLTKGKIISHHIRHMDIVTCL<br>ATDYCGIHLISGSRDTTCMIWQITQQGGVPVGLASKPFQILYGHTDEVLSVGISTELDMAVSGSRDGTV<br>IIHTIQKGQYMRTLRPPCESSLLLTIPSLAISWEGHIVIYSSIEERTTLKDKNALHLFSVNGKYLGSQVLKE<br>QVSDMCIIGEHIVTGSLQGFLSIRDLHSLNLSINPLAMRLPIHCVCVTKEHSHILVGLEDGKLIVVGVGK<br>PAEVKPSIKNLISHSIGDSFGYSYFQLNQKSPLWVNKLKNKFDFSKYSK	
46	Uncharacterized protein	A0A287AJ02	Medium in heart left ventricle	The same (7) +<br>GO:0005515<br>GO:0005506<br>GO:0016705<br>GO:0020037	Match to NLHLP at 1753<br>MWRFLGLGSNPGHSSDNAESLITRPPENSLIHILKQHIYLPECHLEISYYCFFQLINSYLFNRSECIFLKNS<br>RHIFNITMVFLFQKDPDYLKLWLDNFVSSYEQFLDVDFEKLPTRVDDVPPGISLLPDNILQVLRSQLLQ<br>CVQKMADGLEEEQQALSILLVKFFIILCRNLSNVEEIGTCSYINHVITMTTLYIQQLKSKKKEKELADQT<br>SIEEFVIHALAFCESLYDPYRNWRHRISGRILSTVEKSRQKYKPASLTVEFVPFFYQCFQESEHLKESLKC<br>CLLHLFGAIVAGGQKNALQAISPATMEVLMRVLADCDSWEDGNPEEVGRKVELTLKCLTEVVHILLT<br>SSSDQRQVETSSILENYFKLLNSDHSALPNQRRSRQWESRFIALQIKMLNTITAMLDCTDRPVLQAIFL<br>NSNCFEHLIRLLQNCKLFLNANNKVADKNEKDLANKLLTEMNEDQVFQGQLDCLAVSAIQALTAV<br>MNKSPAAKEVFKERIGYTHMFEVLKSLGQPPLELLKELMNMAVEGDHTSVGILGISNVQPLLLLIQWL<br>PEIESHDLQIFISDWLKRICCINRQSRTTCVNANMGIRIIETLDSHSSLHRTCAENLIALHGSLGSQSVSSE<br>EIRQLLRLLRVDEPEYIHPYITPVTRAILTMARKQSLESALQYFNLSHSMAGISVPSIQKWPGSAFSFNA<br>WFCLDQDQLTLGSANKGGKRKQLYSFFTGSGMGFEAFITHSGTLVVAVCTKREYATVMLPDHSFCDS<br>LWHNITIVHMPGKRPFGQSLVYIYDNGQQKVYAPLRFPAMNEPFISCCIGSAGQRTTTPPPSQIPDPPF<br>SSPITPHRTSFGGILSSASWGGTVEKSKLITKLISAGTQDSEWGCPTSLEGQLGSVIIFSEALQPPQVKALY<br>LAGPNCLSPWKFQESDMADLPANVLLHYTAKACKNSICLDLSTNCLHGRLTGNKVVNWDIKDIINCI<br>GGLNVLFPLMEQIPLLEFGEGQIPEGPRENTVSELITPVEGDWVVLTSTKASESRLERNLIATFILIVKHFI<br>QRHPINQDNLIHSHGVAVLGALLQKVPSTLMDVNVLMAIQLLIEQVSLEKNMQLLQQMYQYLLFDF<br>RIWNRGDFPFRIGHIQYLSTIIKDSRRVFRKKYGVQFLLDTLRIYYGSDCKYNELSLDDIRTIRTSLYGLIK<br>YFLCKGGTHEEIQSIVGYIAAISEEEQLIGILDILFSLLHTSPTRGQLFLLLFEPGNADILYALLLNQKYSD<br>RLREIIFKVMEQMLKCTNVYERSKQRIRLREVGYSGLGLLLNEAPVNTSLIKSLTNQIINTDPAINFKDL<br>LSVVYISHRACVNVRVVICRKILQTLQSQPDAAHQISQQVGWQDTLVRLFLKANFENGNTPHKHIRTI<br>LMKDSDKNIATEDIKRSFDEKTDEEKISSFASAHVSSDQWSLEDRHSLDSNTPLFQEDSSVGELSFKSEN<br>QEEFWHSNPSHLSLDLSGIDSYELSDSGSQMPDSLPSTPSPIESAKSFSVQSNKESSVTNDMGFSDDFTLL<br>ESQERCEEELLQLLTTILNYVMCKGLEKSDDDTWIERGQVFSALTKPGISSELLHPPDEIKLILLQKMLE<br>WAVTENREAKINPVTAENALRLMLIIQDFLQSEGLVNSNMWTEKLLEDMMLLFDSLSVWYSESPVW<br>VKLSQIQIQLLLGFIGRGNLQVCAMASAKLNTLLQTKVIENQDEACYILGKLEHVLRQSIKEQTEIYSFL<br>IPLVRTLVSKIYELLFMNLHLPSLPFANGSSSFFEDFQEYCSSNEWQVYIEKYIVPYMKQYETHTFYDGH<br>ESMALYWKDCYEALMVNMHKRDREGGESKLKFQEFFVEPFNRKARQENLRYNNMLKQLSSQQLAT<br>LRRWKAIKLYLTCERGPWAERKQKPIHWKLANVENYSRMRLKLVPNYNFKTHDEASALRDNLGIQH<br>SQPSSDSLLLEVVKQVKVSDMDEDKLDLPEEEITTRVNIDEKEEQDQKEKLVLSEDCELITIIDVIPGRLE<br>ITTQHIYFYDGSIEKEDGVGFDFKWPHSQVREIHLRRYNLRRSALEIFHVDQSNYFLNFKKEVRNKVYS<br>RLLSLHSPNSYGTRSPQELFKTSGLTQKWVNREISNFDYLIQLNTMAGRTYNDLAQYPVFPWILQDYT<br>SEELDLNNPSVFRDLSKPIGVVNDKNAKAMREKYENFEDPMGTIDKFHYGTHYSNSAGVMHYLIRTE<br>PFTTLHIQLQSGRFDCADRQFHSIPATWQALMDNPYDVKELIPEFFYFPEFLENQNKFNLGRLQVSKE<br>VVNDVILPKWAKSAEDFIYKHRKALESEYVSAHLHEWIDLIFGYKQRGPAAVEALNVFYYCSYEGAV<br>DLDALTDEKERKALEGMINNFGQTPCQLLKEPHPSRLSAEEAVQKQTKTDTSTLNLFQHLPELKSFFIE<br>GISDGIPLVKAIVPKNQSRSFMSQGSPELLITVSMNYVIGTHGWLPYDRTISNYFTFIKDQTVTNPKTQR<br>VMNGPFAPGLEITSKLFIVSHDAKLLFSAGHWDNSIQVMSLTKGKIISHHIRHMDIVTCLATDYCGIHL<br>ISGSRDTTCMIWQITQQGGVPVGLASKPFQILYGHTDEVLSVGISTELDMAVSGSRDGTVIIHTIQKGQY<br>MRTLRPPCESSLLLTIPSLAISWEGHIVIYSSIEERTTLKDKNALHLFSVNGKYLGSQVLKEQVSDMCIIG<br>EHIVTGSLQGFLSIRDLHSLNLSINPLAMRLPIHCVCVTKEHSHILVGLEDGKLIVVGVGKPAEASRQA<br>AGIPGITPTEEKDGNLPDIVNSGSLHEFLVNLHERYGPVVSFWFGRRLVVSLGTVDVLKQHINPNKTS<br>DPFETMLKSLLRYQSDSGNMSENHMRKKLYENGVTNSLQSNFALLLKLSEELLDKWLAYPESQHVPL<br>CQHMLGFAMKSVTQMVMGSTFEDEQEVIRFQKNHGTVWSEIGKGFLDGSLDKSTTRKKQYEDALM	90% with<br>A0A480E999<br>(45)

					QLESILKKIIKERKGRNFSQHVFIDSLVQGNLNDQQILEDSMIFSLASCVITAKLCTWTICFLTTYEEVQK<br>KLYEEIDQVFGKGPIIPEKIEELRYCRQVLCETVRTAKLTPVSARLQDIEGKIDTFIIPKETLVLYALGVVL<br>QDPSTWSSPYKFDPERFDDESVMKTFSLLGFSGPQECPELRFAYMVTTVLLSVLVRRLHLLSVEGQVIET<br>KYELVTSSKEEAWITVSKRY	
47	Uncharacterized protein	A0A5G2QLE9	No data	The same (13)	Match to NLHLP at 27<br>MVLVWLLLLLTLLAGARLLWGQWKLRNLHLPPLVPGFLHLLQPNLPIYLLGLTQRLGPIYRLRLGLQ<br>DVVVLNSKRTIEEALVRKWVDFAGRPQIPSYKLASQHCPDISLGDYSLFWKAHKKLTRSALLLGVRSS<br>MEPRVEQLTQEFCERMRAQAGTPVTIQKEFSVLTCSIICCLTFGDKEDTLVHALHDCVQDLMKTWEH<br>WSIQILDMVPFLRVRRWSPGPQGPEREGGGGEQGSFPAATRSCSLPQFFPSPGLRRLKQAIENRDHLVE<br>KQLRRHKESMVAGQWRDMLDYMLQEAGRQRVEEGQGQLLEGHVHMSVVDLFIGGTETTANTLSW<br>AVVYLLHHPEIQWRLQEELDRELGPGAAGSRVPYKDRARLPLLNATIAEVLRLRPVVPLALPHRATRP<br>SSIFGYDIPEGTVVIPNLQGAHLDETVWEQPHEFRPDRFLAPGANPSALAFGCGARVCLGEPLARLELF<br>VVLVQLLQAFTLLPPEGALPSLQPHPHSGINLKVQPFQVRLQPRGGRGEGPGPR	90% with P15540 (1), Q6B7Q1 (10), Q6B7P7 (11) and A5A8W5 (14)
48	Uncharacterized protein	A0A4X1VA33	No data	The same (13)	Match to NLHLP at 27<br>LVLVWLLLLLTLLAGARLLWGQWKLRNLHLPPLVPGFLHLLQPNLPIYLLGLTQRLGPIYRLRLGLQD<br>VVVLNSKRTIEEALVRKWVDFAGRPQIPSYKLASQHSLLLGVRSSMEPRVEQLTQEFCERMRAQAGTP<br>VTIQKEFSVLTCSIICCLTFGDKEDTLVHALHDCVQDLMKTWEHWSIQILDMVPFLRFFPSPGLRRLKQ<br>AIENRDHLVEKQLRRHKESMVAGQWRDMLDYMLQEAGRQRVEEGQGQLLEGHVHMSVVDLFIGG<br>TETTANTLSWAVVYLLHHPEIQWRLQEELDRELGPGAAGSRVPYKDRARLPLLNATIAEVLRLRPVVP<br>LALPHRATRPSSIFGYDIPEGTVVIPNLQGAHLDETVWEQPHEFRPDRFLAPGANPSALAFGCGARVC<br>LGEPLARLELFVVLVQLLQAFTLLPPEGALPSLQPHPHSGINLKVQPFQVRLQPRGGRGEGPGPR	90% with A5A8W7 (13)
49	Uncharacterized protein	F1SHE6	High-Medium in heart left ventricle (86.07), medium in heart (64.63) low in endocardial endothelium (45.36)	Molecular Function protein binding (GO:0005515)	Match to NLHLP at 1656<br>MASRERLFELWMLYCAKKDPDYLKLWLDNFVSSYEQFLDVDFEKLPTRVDDVPPGISLLPDNILQVLR<br>SQLLQCVQKMADGLEEEQQALSILLVKFFIILCRNLSNVEEIGTCSYINHVITMTTLYIQQLKSKKKEKE<br>LADQTSIEEFVIHALAFCESLYDPYRNWRHRISGRILSTVEKSRQKYKPASLTVEFVPFFYQCFQESEHL<br>KESLKCCLLHLFGAIVAGGQKNALQAISPATMEVLMRVLADCDSWEDGNPEEVGRKVELTLKCLTE<br>VVHILLTSSSDQRQVETSSILENYFKLLNSDHSALPNQRRSRQWESRFIALQIKMLNTITAMLDCTDRP<br>VLQAIFLNSNCFEHLIRLLQNCKVFQGQLDCLAVSAIQALTAVMNKSPAAKEVFKERIGYTHMFEVL<br>KSLGQPPLELLKELMNMAVEGDHTSVGILGISNVQPLLLLIQWLPEIESHDLQIFISDWLKRICCINRQS<br>RTTCVNANMGIRIIETLDSHSSLHRTCAENLIALHGSLGSQSVSSEEIRQLLRLLRVDEPEYIHPYITPVTR<br>AILTMARKQSLESALQYFNLSHSMAGISVPSIQKWPGSAFSFNAWFCLDQDQLTLGSANKGGKRKQL<br>YSFFTGSGMGFEAFITHSGTLVVAVCTKREYATVMLPDHSFCDSLWHNITIVHMPGKRPFGQSLVYIY<br>DNGQQKVYAPLRFPAMNEPFISCCIGSAGQRTTTPPPSQIPDPPFSSPITPHRTSFGGILSSASWGGTVEK<br>SKLITKLISAGTQDSEWGCPTSLEGQLGSVIIFSEALQPPQVKALYLAGPNCLSPWKFQESDMADLPAN<br>VLLHYTAKACKNSICLDLSTNCLHGRLTGNKVVNWDIKDIINCIGGLNVLFPLMEQIPLLEFGEGQIPE<br>GPRENTVSELITPVEGDWVVLTSTKASESRLERNLIATFILIVKHFIQRHPINQDNLIHSHGVAVLGALL<br>QKVPSTLMDVNVLMAIQLLIEQVSLEKNMQLLQQMYQYLLFDFRIWNRGDFPFRIGHIQYLSTIIKDSR<br>RVFRKKYGVQFLLDTLRIYYGSDCKYNELSLDDIRTIRTSLYGLIKYFLCKGGTHEEIQSIVGYIAAISEEE<br>QLIGILDILFSLLHTSPTRGQLFLLLFEPGNADILYALLLNQKYSDRLREIIFKVMEQMLKCTNVYERSKQ<br>RIRLREVGYSGLGLLLNEAPVNTSLIKSLTNQIINTDPAINFKDLLSVVYISHRACVNVRVVICRKILQTL<br>QSQPDAAHQISQQVGWQDTLVRLFLKANFENGNTPHKHIRTILMKDSDKNIATEDIKRSFDEKTDEE<br>KISSFASAHVSSDQWSLEDRHSLDSNTPLFQEDSSVGELSFKSENQEEFWHSNPSHLSLDLSGIDSYELS<br>DSGSQMPDSLPSTPSPIESAKSFSVQSNKESSVTNDMGFSDDFTLLESQERCEEELLQLLTTILNYVMCK<br>GLEKSDDDTWIERGQVFSALTKPGISSELLHPPDEIKLILLQKMLEWAVTENREAKINPVTAENALRL<br>MLIIQDFLQSEGLVNSNMWTEKLLEDMMLLFDSLSVWYSESPVWVKLSQIQIQLLLGFIGRGNLQVCA<br>MASAKLNTLLQTKVIENQDEACYILGKLEHVLRQSIKEQTEIYSFLIPLVRTLVSKIYELLFMNLHLPSLP<br>FANGSSSFFEDFQEYCSSNEWQVYIEKYIVPYMKQYETHTFYDGHESMALYWKDCYEALMVNMHKR<br>DREGGESKLKFQEFFVEPFNRKARQENLRYNNMLKQLSSQQLATLRRWKAIKLYLTCERGPWAERK	90% with A0A287AJ02 (46) and A0A480E999 (45)

					QKPIHWKLANVENYSRMRLKLVPNYNFKTHDEASALRDNLGIQHSQPSSDSLLLEVVKQVKVSDMD<br>EDKLDLPEEEITTRVNIDEKEEQDQKEKLVLSEDCELITIIDVIPGRLEITTQHIYFYDGSIEKEDGVGFDF<br>KWPHSQVREIHLRRYNLRRSALEIFHVDQSNYFLNFKKEVRNKVYSRLLSLHSPNSYGTRSPQELFKTS<br>GLTQKWVNREISNFDYLIQLNTMAGRTYNDLAQYPVFPWILQDYTSEELDLNNPSVFRDLSKPIGVV<br>NDKNAKAMREKYENFEDPMGTIDKFHYGTHYSNSAGVMHYLIRTEPFTTLHIQLQSGRFDCADRQF<br>HSIPATWQALMDNPYDVKELIPEFFYFPEFLENQNKFNLGRLQVSKEVVNDVILPKWAKSAEDFIYKH<br>RKALESEYVSAHLHEWIDLIFGYKQRGPAAVEALNVFYYCSYEGAVDLDALTDEKERKALEGMINNF<br>GQTPCQLLKEPHPSRLSAEEAVQKQTKTDTSTLNLFQHLPELKSFFIEGISDGIPLVKAIVPKNQSRSFM<br>SQGSPELLITVSMNYVIGTHGWLPYDRTISNYFTFIKDQTVTNPKTQRVMNGPFAPGLEITSKLFIVSHD<br>AKLLFSAGHWDNSIQVMSLTKGKIISHHIRHMDIVTCLATDYCGIHLISGSRDTTCMIWQITQQGGVP<br>VGLASKPFQILYGHTDEVLSVGISTELDMAVSGSRDGTVIIHTIQKGQYMRTLRPPCESSLLLTIPSLAIS<br>WEGHIVIYSSIEERTTLKDKNALHLFSVNGKYLGSQVLKEQVSDMCIIGEHIVTGSLQGFLSIRDLHSLN<br>LSINPLAMRLPIHCVCVTKEHSHILVGLEDGKLIVVGVGKPAEMRSGQLSRKLWGSSKRLSQISAGETE<br>YNTQDSK	
50	Uncharacterized protein	A0A287BFX1	Medium in heart left ventricle	GO:0005515	Match to NLHLP at 1608<br>MADGLEEEQQALSILLVKFFIILCRNLSNVEEIGTCSYINHVITMTTLYIQQLKSKKKEKELADQTSIEEF<br>VIHALAFCESLYDPYRNWRHRISGRILSTVEKSRQKYKPASLTVEFVPFFYQCFQESEHLKESLKCCLLH<br>LFGAIVAGGQKNALQAISPATMEVLMRVLADCDSWEDGNPEEVGRKVELTLKCLTEVVHILLTSSSD<br>QRQVETSSILENYFKLLNSDHSALPNQRRSRQWESRFIALQIKMLNTITAMLDCTDRPVLQAIFLNSNC<br>FEHLIRLLQNCKLFLNANNKVADKNEKDLANKLLTEMNEDQVFQGQLDCLAVSAIQALTAVMNKS<br>PAAKEVFKERIGYTHMFEVLKSLGQPPLELLKELMNMAVEGDHTSVGILGISNVQPLLLLIQWLPEIES<br>HDLQIFISDWLKRICCINRQSRTTCVNANMGIRIIETLDSHSSLHRTCAENLIALHGSLGSQSVSSEEIRQ<br>LLRLLRVDEPEYIHPYITPVTRAILTMARKQSLESALQYFNLSHSMAGISVPSIQKWPGSAFSFNAWFCL<br>DQDQLTLGSANKGGKRKQLYSFFTGSGMGFEAFITHSGTLVVAVCTKREYATVMLPDHSFCDSLWH<br>NITIVHMPGKRPFGQSLVYIYDNGQQKVYAPLRFPAMNEPFISCCIGSAGQRTTTPPPSQIPDPPFSSPIT<br>PHRTSFGGILSSASWGGTVEKSKLITKLISAGTQDSEWGCPTSLEGQLGSVIIFSEALQPPQVKALYLAG<br>PNCLSPWKFQESDMADLPANVLLHYTAKACKNSICLDLSTNCLHGRLTGNKVVNWDIKDIINCIGGL<br>NVLFPLMEQIPLLEFGEGQIPEGPRENTVSELITPVEGDWVVLTSTKASESRLERNLIATFILIVKHFIQR<br>HPINQDNLIHSHGVAVLGALLQKVPSTLMDVNVLMAIQLLIEQVSLEKNMQLLQQMYQYLLFDFRI<br>WNRGDFPFRIGHIQYLSTIIKDSRRVFRKKYGVQFLLDTLRIYYGSDCKYNELSLDDIRTIRTSLYGLIKYF<br>LCKGGTHEEIQSIVGYIAAISEEEQLIGILDILFSLLHTSPTRGQLFLLLFEPGNADILYALLLNQKYSDRL<br>REIIFKVMEQMLKCTNVYERSKQRIRLREVGYSGLGLLLNEAPVNTSLIKSLTNQIINTDPAINFKDLLS<br>VVYISHRACVNVRVVICRKILQTLQSQPDAAHQISQQVGWQDTLVRLFLKANFENGNTPHKHIRTIL<br>MKDSDKNIATEDIKRSFDEKTDEEKISSFASAHVSSDQWSLEDRHSLDSNTPLFQEDSSVGELSFKSENQ<br>EEFWHSNPSHLSLDLSGIDSYELSDSGSQMPDSLPSTPSPIESAKSFSVQSNKESSVTNDMGFSDDFTLLE<br>SQERCEEELLQLLTTILNYVMCKGLEKSDDDTWIERGQVFSALTKPGISSELLHPPDEIKLILLQKMLEW<br>AVTENREAKINPVTAENALRLMLIIQDFLQSEGLVNSNMWTEKLLEDMMLLFDSLSVWYSESPVWVK<br>LSQIQIQLLLGFIGRGNLQVCAMASAKLNTLLQTKVIENQDEACYILGKLEHVLRQSIKEQTEIYSFLIPL<br>VRTLVSKIYELLFMNLHLPSLPFANGSSSFFEDFQEYCSSNEWQVYIEKYIVPYMKQYETHTFYDGHES<br>MALYWKDCYEALMVNMHKRDREGGESKLKFQEFFVEPFNRKARQENLRYNNMLKQLSSQQLATLR<br>RWKAIKLYLTCERGPWAERKQKPIHWKLANVENYSRMRLKLVPNYNFKTHDEASALRDNLGIQHS<br>QPSSDSLLLEVVKQVKVSDMDEDKLDLPEEEITTRVNIDEKEEQDQKEKLVLSEDCELITIIDVIPGRLEI<br>TTQHIYFYDGSIEKEDGVGFDFKWPHSQVREIHLRRYNLRRSALEIFHVDQSNYFLNFKKEVRNKVYS<br>RLLSLHSPNSYGTRSPQELFKTSGLTQKWVNREISNFDYLIQLNTMAGRTYNDLAQYPVFPWILQDYT<br>SEELDLNNPSVFRDLSKPIGVVNDKNAKAMREKYENFEDPMGTIDKFHYGTHYSNSAGVMHYLIRTE<br>PFTTLHIQLQSGRFDCADRQFHSIPATWQALMDNPYDVKELIPEFFYFPEFLENQNKFNLGRLQVSKE<br>VVNDVILPKWAKSAEDFIYKHRKALESEYVSAHLHEWIDLIFGYKQRGPAAVEALNVFYYCSYEGAV<br>DLDALTDEKERKALEGMINNFGQTPCQLLKEPHPSRLSAEEAVQKQTKTDTSTLNLFQHLPELKSFFIE<br>GISDGIPLVKAIVPKNQSRSFMSQGSPELLITVSMNYVIGTHGWLPYDRTISNYFTFIKDQTVTNPKTQR	90% with A0A287AJ02 (46), F1SHE6 (49) and A0A480E999 (45)

					VMNGPFAPGLEITSKLFIVSHDAKLLFSAGHWDNSIQVMSLTKGKIISHHIRHMDIVTCLATDYCGIHL<br>ISGSRDTTCMIWQITQQGGVPVGLASKPFQILYGHTDEVLSVGISTELDMAVSGSRDGTVIIHTIQKGQY<br>MRTLRPPCESSLLLTIPSLAISWEGHIVIYSSIEERTTLKDKNALHLFSVNGKYLGSQVLKEQVSDMCIIG<br>EHIVTGSLQGFLSIRDLHSLNLSINPLAMRLPIHCVCVTKEHSHILVGLEDGKLIVVGVGKPAEMRSGQ<br>LSRKLWGSSKRLSQISAGETEYNTQDSK	
51	Uncharacterized protein	A0A286ZRL3	Medium in heart left ventricle	GO:0005515<br>GO:0005506<br>GO:0016705<br>GO:0020037<br>GO:0019901<br>GO:0008104	Match to NLHLP at 1685<br>MASRERLFELWMLYCAKKDPDYLKLWLDNFVSSYEQFLDVDFEKLPTRVDDVPPGISLLPDNILQVLR<br>SQLLQCVQKMADGLEEEQQALSILLVKFFIILCRNLSNVEEIGTCSYINHVITMTTLYIQQLKSKKKEKE<br>LADQTSIEEFVIHALAFCESLYDPYRNWRHRISGRILSTVEKSRQKYKPASLTVEFVPFFYQCFQESEHL<br>KESLKCCLLHLFGAIVAGGQKNALQAISPATMEVLMRVLADCDSWEDGNPEEVGRKVELTLKCLTE<br>VVHILLTSSSDQRQVETSSILENYFKLLNSDHSALPNQRRSRQWESRFIALQIKMLNTITAMLDCTDRP<br>VLQAIFLNSNCFEHLIRLLQNCKLFLNANNKVADKNEKDLANKLLTEMNEDQVFQGQLDCLAVSAI<br>QALTAVMNKSPAAKEVFKERIGYTHMFEVLKSLGQPPLELLKELMNMAVEGDHTSVGILGISNVQPL<br>LLLIQWLPEIESHDLQIFISDWLKRICCINRQSRTTCVNANMGIRIIETLDSHSSLHRTCAENLIALHGSL<br>GSQSVSSEEIRQLLRLLRVDEPEYIHPYITPVTRAILTMARKQSLESALQYFNLSHSMAGISVPSIQKWPG<br>SAFSFNAWFCLDQDQLTLGSANKGGKRKQLYSFFTGSGMGFEAFITHSGTLVVAVCTKREYATVMLP<br>DHSFCDSLWHNITIVHMPGKRPFGQSLVYIYDNGQQKVYAPLRFPAMNEPFISCCIGSAGQRTTTPPPS<br>QIPDPPFSSPITPHRTSFGGILSSASWGGTVEKSKLITKLISAGTQDSEWGCPTSLEGQLGSVIIFSEALQPP<br>QVKALYLAGPNCLSPWKFQESDMADLPANVLLHYTAKACKNSICLDLSTNCLHGRLTGNKVVNWD<br>IKDIINCIGGLNVLFPLMEQIPLLEFGEGQIPEGPRENTVSELITPVEGDWVVLTSTKASESRLERNLIATF<br>ILIVKHFIQRHPINQDNLIHSHGVAVLGALLQKVPSTLMDVNVLMAIQLLIEQVSLEKNMQLLQQMY<br>QYLLFDFRIWNRGDFPFRIGHIQYLSTIIKDSRRVFRKKYGVQFLLDTLRIYYGSDCKYNELSLDDIRTIR<br>TSLYGLIKYFLCKGGTHEEIQSIVGYIAAISEEEQLIGILDILFSLLHTSPTRGQLFLLLFEPGNADILYALL<br>LNQKYSDRLREIIFKVMEQMLKCTNVYERSKQRIRLREVGYSGLGLLLNEAPVNTSLIKSLTNQIINTDP<br>AINFKDLLSVVYISHRACVNVRVVICRKILQTLQSQPDAAHQISQQVGWQDTLVRLFLKANFENGNT<br>PHKHIRTILMKDSDKNIATEDIKRSFDEKTDEEKISSFASAHVSSDQWSLEDRHSLDSNTPLFQEDSSVG<br>ELSFKSENQEEFWHSNPSHLSLDLSGIDSYELSDSGSQMPDSLPSTPSPIESAKSFSVQSNKESSVTNDMG<br>FSDDFTLLESQERCEEELLQLLTTILNYVMCKGLEKSDDDTWIERGQVFSALTKPGISSELLHPPDEIKLI<br>LLQKMLEWAVTENREAKINPVTAENALRLMLIIQDFLQSEGLVNSNMWTEKLLEDMMLLFDSLSVW<br>YSESPVWVKLSQIQIQLLLGFIGRGNLQVCAMASAKLNTLLQTKVIENQDEACYILGKLEHVLRQSIKE<br>QTEIYSFLIPLVRTLVSKIYELLFMNLHLPSLPFANGSSSFFEDFQEYCSSNEWQVYIEKYIVPYMKQYET<br>HTFYDGHESMALYWKDCYEALMVNMHKRDREGGESKLKFQEFFVEPFNRKARQENLRYNNMLKQ<br>LSSQQLATLRRWKAIKLYLTCERGPWAERKQKPIHWKLANVENYSRMRLKLVPNYNFKTHDEASAL<br>RDNLGIQHSQPSSDSLLLEVVKQVKVSDMDEDKLDLPEEEITTRVNIDEKEEQDQKEKLVLSEDCELITI<br>IDVIPGRLEITTQHIYFYDGSIEKEDGVGFDFKWPHSQVREIHLRRYNLRRSALEIFHVDQSNYFLNFKK<br>EVRNKVYSRLLSLHSPNSYGTRSPQELFKTSGLTQKWVNREISNFDYLIQLNTMAGRTYNDLAQYPVF<br>PWILQDYTSEELDLNNPSVFRDLSKPIGVVNDKNAKAMREKYENFEDPMGTIDKFHYGTHYSNSAGV<br>MHYLIRTEPFTTLHIQLQSGRFDCADRQFHSIPATWQALMDNPYDVKELIPEFFYFPEFLENQNKFNL<br>GRLQVSKEVVNDVILPKWAKSAEDFIYKHRKALESEYVSAHLHEWIDLIFGYKQRGPAAVEALNVFY<br>YCSYEGAVDLDALTDEKERKALEGMINNFGQTPCQLLKEPHPSRLSAEEAVQKQTKTDTSTLNLFQH<br>LPELKSFFIEGISDGIPLVKAIVPKNQSRSFMSQGSPELLITVSMNYVIGTHGWLPYDRTISNYFTFIKDQT<br>VTNPKTQRVMNGPFAPGLEITSKLFIVSHDAKLLFSAGHWDNSIQVMSLTKGKIISHHIRHMDIVTCL<br>ATDYCGIHLISGSRDTTCMIWQITQQGGVPVGLASKPFQILYGHTDEVLSVGISTELDMAVSGSRDGTV<br>IIHTIQKGQYMRTLRPPCESSLLLTIPSLAISWEGHIVIYSSIEERTTLKDKNALHLFSVNGKYLGSQVLKE<br>QVSDMCIIGEHIVTGSLQGFLSIRDLHSLNLSINPLAMRLPIHCVCVTKEHSHILVGLEDGKLIVVGVGK<br>PAEASRQAAGIPGITPTEEKDGNLPDIVNSGSLHEFLVNLHERYGPVVSFWFGRRLVVSLGTVDVLKQ<br>HINPNKTSDPFETMLKSLLRYQSDSGNMSENHMRKKLYENGVTNSLQSNFALLLKLSEELLDKWLAY<br>PESQHVPLCQHMLGFAMKSVTQMVMGSTFEDEQEVIRFQKNHGTVWSEIGKGFLDGSLDKSTTRKK<br>QYEDALMQLESILKKIIKERKGRNFSQHVFIDSLVQGNLNDQQILEDSMIFSLASCVITAKLCTWTICFL	90% with A0A287AJ02 (46), F1SHE6 (49), A0A287BFX1 (50) and A0A480E999 (45)

					TTYEEVQKKLYEEIDQVFGKGPIIPEKIEELRYCRQVLCETVRTAKLTPVSARLQDIEGKIDTFIIPKETLV<br>LYALGVVLQDPSTWSSPYKFDPERFDDESVMKTFSLLGFSGPQECPELRFAYMVTTVLLSVLVRRLHLL<br>SVEGQVIETKYELVTSSKEEAWITVSKRY	
52	Uncharacterized protein	A0A4X1V6R6	No data	The same (13)	Match to NLHLP at 27<br>MVLVGLLLLLTLLAGARLLWGQWKLRNLHLPPLVPGFLHLLQPNLPIYLLGLTQRLGPIYRLRLGLQD<br>KLASQHCPDISLGDYLFWKAHKKLTRSALLLGVRSSMEPRVEQLTQEFCERMRAQAGTPVTIQKEFSV<br>LTCSIICCLTFGDKVKEDTLVHALHDCVQDLMKTWEHWSIQILDMVPFLRFFPSPGLRRLKQAIENRD<br>HLVEKQLRRHKESMVAGQWRDMLDYMLQEAGRQRVEEGQGQLLEGHVHMSVVDLFIGGTETTAN<br>TLSWAVVYLLHHPEIQWRLQEELDRELGPGAAGSRVPYKDRARLPLLNATIAEVLRLRPVVPLALPH<br>RATRPSSIFGYDIPEGTVVIPNLQGAHLDETVWEQPHEFRPDRFLAPGANPSALAFGCGARVCLGEPL<br>ARLELFVVLVQLLQAFTLLPPEGALPSLQPHPHSGINLKVQPFQVRLQPRGGRAQGPGEHL	90% with A5A8W7 (13)
53	Uncharacterized protein	A0A4X1V4K3	No data	The same (7)<br>+ GO:0005515<br>GO:0005506<br>GO:0016705<br>GO:0020037	Match to NLHLP at 1686<br>MHFLKNSRHIFNITMVFLFQKDPDYLKLWLDNFVSSYEQFLDVDFEKLPTRVDDVPPGISLLPDNILQV<br>LRSQLLQCVQKMADGLEEEQQALSILLVKFFIILCRNLSNVEEIGTCSYINHVITMTTLYIQQLKSKKKE<br>KELADQTSIEEFVIHALAFCESLYDPYRNWRHRISGRILSTVEKSRQKYKPASLTVEFVPFFYQCFQESE<br>HLKESLKCCLLHLFGAIVAGGQKNALQAISPATMEVLMRVLADCDSWEDGNPEEVGRKVELTLKCL<br>TEVVHILLTSSSDQRQVETSSILENYFKLLNSDHSALPNQRRSRQWESRFIALQIKMLNTITAMLDCTDR<br>PVLQAIFLNSNCFEHLIRLLQNCKLFLNANNKVADKNEKDLANKLLTEMNEDQVFQGQLDCLAVSA<br>IQALTAVMNKSPAAKEVFKERIGYTHMFEVLKSLGQPPLELLKELMNMAVEGDHTSVGILGISNVQP<br>LLLLIQWLPEIESHDLQIFISDWLKRICCINRQSRTTCVNANMGIRIIETLDSHSSLHRTCAENLIALHGS<br>LGSQSVSSEEIRQLLRLLRVDEPEYIHPYITPVTRAILTMARKQSLESALQYFNLSHSMAGISVPSIQKWP<br>GSAFSFNAWFCLDQDQLTLGSANKGGKRKQLYSFFTGSGMGFEAFITHSGTLVVAVCTKREYATVML<br>PDHSFCDSLWHNITIVHMPGKRPFGQSLVYIYDNGQQKVYAPLRFPAMNEPFISCCIGSAGQRTTTPP<br>PSQIPDPPFSSPITPHRTSFGGILSSASWGGTVEKSKLITKLISAGTQDSEWGCPTSLEGQLGSVIIFSEALQ<br>PPQVKALYLAGPNCLSPWKFQESDMADLPANVLLHYTAKACKNSICLDLSTNCLHGRLTGNKVVN<br>WDIKDIINCIGGLNVLFPLMEQIRHFGEGQIPEGPRENTVSELITPVEGDWVVLTSTKASESRLERNLIA<br>TFILIVKHFIQRHPINQDNLIHSHGVAVLGALLQKVPSTLMDVNVLMAIQLLIEQVSLEKNMQLLQQ<br>MYQYLLFDFRIWNRGDFPFRIGHIQYLSTIIKDSRRVFRKKYGVQFLLDTLRIYYGSDCKYNELSLDDIR<br>TIRTSLYGLIKYFLCKGGTHEEIQSIVGYIAAISEEEQLIGILDILFSLLHTSPTRGQLFLLLFEPGNADILYA<br>LLLNQKYSDRLREIIFKVMEQMLKCTNVYERSKQRIRLREVGYSGLGLLLNEAPVNTSLIKSLTNQIINT<br>DPAINFKDLLSVVYISHRACVNVRVVICRKILQTLQSQPDAAHQISQQVGWQDTLVRLFLKANFENG<br>NTPHKHIRTILMKDSDKNIATEDIKRSFDEKTDEEKISSFASAHVSSDQWSLEDRHSLDSNTPLFQEDSS<br>VGELSFKSENQEEFWHSNPSHLSLDLSGIDSYELSDSGSQMPDSLPSTPSPIESAKSFSVQSNKESSVTND<br>MGFSDDFTLLESQERCEEELLQLLTTILNYVMCKGLEKSDDDTWIERGQVFSALTKPGISSELLHPPDEI<br>KLILLQKMLEWAVTENREAKINPVTAENALRLMLIIQDFLQSEGLVNSNMWTEKLLEDMMLLFDSLS<br>VWYSESPVWVKLSQIQIQLLLGFIGRGNLQVCAMASAKLNTLLQTKVIENQDEACYILGKLEHVLRQS<br>IKEQTEIYSFLIPLVRTLVSKIYELLFMNLHLPSLPFANGSSSFFEDFQEYCSSNEWQVYIEKYIVPYMKQY<br>ETHTFYDGHESMALYWKDCYEALMVNMHKRDREGGESKLKFQEFFVEPFNRKARQENLRYNNML<br>KQLSSQQLATLRRWKAIKLYLTCERGPWAERKQKPIHWKLANVENYSRMRLKLVPNYNFKTHDEAS<br>ALRDNLGIQHSQPSSDSLLLEVVKQVKVSDMDEDKLDLPEEEITTRVNIDEKEEQDQKEKLVLSEDCEL<br>ITIIDVIPGRLEITTQHIYFYDGSIEKEDGVGFDFKWPHSQVREIHLRRYNLRRSALEIFHVDQSNYFLNF<br>KKEVRNKVYSRLLSLHSPNSYGTRSPQELFKTSGLTQKWVNREISNFDYLIQLNTMAGRTYNDLAQYP<br>VFPWILQDYTSEELDLNNPSVFRDLSKPIGVVNDKNAKAMREKYENFEDPMGTIDKFHYGTHYSNSA<br>GVMHYLIRTEPFTTLHIQLQSGRFDCADRQFHSIPATWQALMDNPYDVKELIPEFFYFPEFLENQNKF<br>NLGRLQVSKEVVNDVILPKWAKSAEDFIYKHRKALESEYVSAHLHEWIDLIFGYKQRGPAAVEALNV<br>FYYCSYEGAVDLDALTDEKERKALEGMINNFGQTPCQLLKEPHPSRLSAEEAVQKQTKTDTSTLNLF<br>QHLPELKSFFIEGISDGIPLVKAIVPKNQSRSFMSQGSPELLITVSMNYVIGTHGWLPYDRTISNYFTFIK<br>DQTVTNPKTQRVMNGPFAPGLEITSKLFIVSHDAKLLFSAGHWDNSIQVMSLTKGKIISHHIRHMDIV	90% with A0A287AJ02 (46), F1SHE6 (49), A0A287BFX1 (50), A0A286ZRL3 (51) and A0A480E999 (45)

					TCLATDYCGIHLISGSRDTTCMIWQITQQGGVPVGLASKPFQILYGHTDEVLSVGISTELDMAVSGSRD<br>GTVIIHTIQKGQYMRTLRPPCESSLLLTIPSLAISWEGHIVIYSSIEERTTLKDKNALHLFSVNGKYLGSQV<br>LKEQVSDMCIIGEHIVTGSLQGFLSIRDLHSLNLSINPLAMRLPIHCVCVTKEHSHILVGLEDGKLIVVG<br>VGKPAEASRQAAGIPGITPTEEKDGNLPDIVNSGSLHEFLVNLHERYGPVVSFWFGRRLVVSLGTVDV<br>LKQHINPNKTSDPFETMLKSLLRYQSDSGNMSENHMRKKLYENGVTNSLQSNFALLLKLSEELLDKW<br>LAYPESQHVPLCQHMLGFAMKSVTQMVMGSTFEDEQEVIRFQKNHGTVWSEIGKGFLDGSLDKSTT<br>RKKQYEDALMQLESILKKIIKERKGRNFSQHVFIDSLVQGNLNDQQILEDSMIFSLASCVITAKLCTWTI<br>CFLTTYEEVQKKLYEEIDQVFGKGPIIPEKIEELRYCRQVLCETVRTAKLTPVSARLQDIEGKIDTFIIPKE<br>TLVLYALGVVLQDPSTWSSPYKFDPERFDDESVMKTFSLLGFSGPQECPELRFAYMVTTVLLSVLVRRL<br>HLLSVEGQVIETKYELVTSSKEEAWITVSKRY	
54	Uncharacterized protein	A0A4X1V6V9	No data	The same (7) + GO:0005515	Match to NLHLP at 1683<br>MASRERLFELWMLYCAKKDPDYLKLWLDNFVSSYEQFLDVDFEKLPTRVDDVPPGISLLPDNILQVLR<br>SQLLQCVQKMADGLEEEQQALSILLVKFFIILCRNLSNVEEIGTCSYINHVITMTTLYIQQLKSKKKEKE<br>LADQTSIEEFVIHALAFCESLYDPYRNWRHRISGRILSTVEKSRQKYKPASLTVEFVPFFYQCFQESEHL<br>KESLKCCLLHLFGAIVAGGQKNALQAISPATMEVLMRVLADCDSWEDGNPEEVGRKVELTLKCLTE<br>VVHILLTSSSDQRQVETSSILENYFKLLNSDHSALPNQRRSRQWESRFIALQIKMLNTITAMLDCTDRP<br>VLQAIFLNSNCFEHLIRLLQNCKLFLNANNKVADKNEKDLANKLLTEMNEDQVFQGQLDCLAVSAI<br>QALTAVMNKSPAAKEVFKERIGYTHMFEVLKSLGQPPLELLKELMNMAVEGDHTSVGILGISNVQPL<br>LLLIQWLPEIESHDLQIFISDWLKRICCINRQSRTTCVNANMGIRIIETLDSHSSLHRTCAENLIALHGSL<br>GSQSVSSEEIRQLLRLLRVDEPEYIHPYITPVTRAILTMARKQSLESALQYFNLSHSMAGISVPSIQKWPG<br>SAFSFNAWFCLDQDQLTLGSANKGGKRKQLYSFFTGSGMGFEAFITHSGTLVVAVCTKREYATVMLP<br>DHSFCDSLWHNITIVHMPGKRPFGQSLVYIYDNGQQKVYAPLRFPAMNEPFISCCIGSAGQRTTTPPPS<br>QIPDPPFSSPITPHRTSFGGILSSASWGGTVEKSKLITKLISAGTQDSEWGCPTSLEGQLGSVIIFSEALQPP<br>QVKALYLAGPNCLSPWKFQESDMADLPANVLLHYTAKACKNSICLDLSTNCLHGRLTGNKVVNWD<br>IKDIINCIGGLNVLFPLMEQIRHFGEGQIPEGPRENTVSELITPVEGDWVVLTSTKASESRLERNLIATFIL<br>IVKHFIQRHPINQDNLIHSHGVAVLGALLQKVPSTLMDVNVLMAIQLLIEQVSLEKNMQLLQQMYQ<br>YLLFDFRIWNRGDFPFRIGHIQYLSTIIKDSRRVFRKKYGVQFLLDTLRIYYGSDCKYNELSLDDIRTIRTS<br>LYGLIKYFLCKGGTHEEIQSIVGYIAAISEEEQLIGILDILFSLLHTSPTRGQLFLLLFEPGNADILYALLLN<br>QKYSDRLREIIFKVMEQMLKCTNVYERSKQRIRLREVGYSGLGLLLNEAPVNTSLIKSLTNQIINTDPAI<br>NFKDLLSVVYISHRACVNVRVVICRKILQTLQSQPDAAHQISQQVGWQDTLVRLFLKANFENGNTPH<br>KHIRTILMKDSDKNIATEDIKRSFDEKTDEEKISSFASAHVSSDQWSLEDRHSLDSNTPLFQEDSSVGEL<br>SFKSENQEEFWHSNPSHLSLDLSGIDSYELSDSGSQMPDSLPSTPSPIESAKSFSVQSNKESSVTNDMGFS<br>DDFTLLESQERCEEELLQLLTTILNYVMCKGLEKSDDDTWIERGQVFSALTKPGISSELLHPPDEIKLILL<br>QKMLEWAVTENREAKINPVTAENALRLMLIIQDFLQSEGLVNSNMWTEKLLEDMMLLFDSLSVWYS<br>ESPVWVKLSQIQIQLLLGFIGRGNLQVCAMASAKLNTLLQTKVIENQDEACYILGKLEHVLRQSIKEQ<br>TEIYSFLIPLVRTLVSKIYELLFMNLHLPSLPFANGSSSFFEDFQEYCSSNEWQVYIEKYIVPYMKQYETH<br>TFYDGHESMALYWKDCYEALMVNMHKRDREGGESKLKFQEFFVEPFNRKARQENLRYNNMLKQLS<br>SQQLATLRRWKAIKLYLTCERGPWAERKQKPIHWKLANVENYSRMRLKLVPNYNFKTHDEASALRD<br>NLGIQHSQPSSDSLLLEVVKQVKVSDMDEDKLDLPEEEITTRVNIDEKEEQDQKEKLVLSEDCELITIID<br>VIPGRLEITTQHIYFYDGSIEKEDGVGFDFKWPHSQVREIHLRRYNLRRSALEIFHVDQSNYFLNFKKEV<br>RNKVYSRLLSLHSPNSYGTRSPQELFKTSGLTQKWVNREISNFDYLIQLNTMAGRTYNDLAQYPVFPW<br>ILQDYTSEELDLNNPSVFRDLSKPIGVVNDKNAKAMREKYENFEDPMGTIDKFHYGTHYSNSAGVM<br>HYLIRTEPFTTLHIQLQSGRFDCADRQFHSIPATWQALMDNPYDVKELIPEFFYFPEFLENQNKFNLGR<br>LQVSKEVVNDVILPKWAKSAEDFIYKHRKALESEYVSAHLHEWIDLIFGYKQRGPAAVEALNVFYYCS<br>YEGAVDLDALTDEKERKALEGMINNFGQTPCQLLKEPHPSRLSAEEAVQKQTKTDTSTLNLFQHLPE<br>LKSFFIEGISDGIPLVKAIVPKNQSRSFMSQGSPELLITVSMNYVIGTHGWLPYDRTISNYFTFIKDQTVT<br>NPKTQRVMNGPFAPGLEITSKLFIVSHDAKLLFSAGHWDNSIQVMSLTKGKIISHHIRHMDIVTCLAT<br>DYCGIHLISGSRDTTCMIWQITQQGGVPVGLASKPFQILYGHTDEVLSVGISTELDMAVSGSRDGTVII<br>HTIQKGQYMRTLRPPCESSLLLTIPSLAISWEGHIVIYSSIEERTTLKDKNALHLFSVNGKYLGSQVLKEQ	90% with A0A287AJ02 (46), F1SHE6 (49), A0A287BFX1 (50), A0A286ZRL3 (51), A0A4X1V4K3 (53) and A0A480E999 (45)

					VSDMCIIGEHIVTGSLQGFLSIRDLHSLNLSINPLAMRLPIHCVCVTKEHSHILVGLEDGKLIVVGVGKP AEASRQAAGIPGITPTEEKDGNLPDIVNSGSLHEFLVNLHERYGPVVSFWFGRRLVVSLGTVDVLKQH INPNKTSDPFETMLKSLLRYQSDSGNMSENHMRKKLYENGVTNSLQSNFALLLKLSEELLDKWLAYP ESQHVPLCQHMLGFAMKSVTQMVMGSTFEDEQEVIRFQKNHGTVWSEIGKGFLDGSLDKSTTRKKQ YEDALMQLESILKKIIKERKGRNFSQHVFIDSLVQGNLNDQQILEDSMIFSLASCVITAKLCTWTICFLT TYEEVQKKLYEEIDQVFGKGPIIPEKIEELRYCRQVLCETVRTAKLTPVSARLQDIEGKIDTFIIPKETLVL YALGVVLQDPSTWSSPYKFDPERFDDESVMKTFSLLGFSGPQECPELRFAYMVTTVLLSVLVRRLHLLS VEGQVIETKYELVTSSKEEAWITVSKRY	
55	Uncharacterized protein	A0A4X1VR69	No data	GO:0005515	Match to NLHLP at 661<br>MSGQPPPPPPPPQQQQQQQQQLPPPPPAAVAPVPGVVLPAPPAVSAGSSPAGSPGGGAGGGGGGLG AAAAALLLHPPPPPPPPATAAPPPPPPPPPPASAAAPATAPPAPPGLAAGPGPAGGAPAPALAAGSS AAAPFPHGDSALNEQEKELQRRLKRLYPAVDEQETPLPRSWSPKDKFSYIGLSQNNLRVHYKGHGKT PKDAASVRATHPIPAACGIYYFEVKIVSKGRDGYMGIGLSAQGVNMNRLPGWDKHSYGYHGDDGH SFCSSGTGQPYGPTFTTGDVIGCCVNLINNTCFYTKNGHSLGIAFTDLPPNLYPTVGLQTPGEVVDANF GQHPFVFDIEDYMREWRTKIQAQIDRFPIGDREGEWQTMIQKMVSSYLVHHGYCATAEAFARSTDQT VLEELASIKNRQRIQKLVLAGRMGEAIETTQQLYPSLLERNPNLLFTLKVRQFIEMVNGTDSEVRCLGG RSPKSQDSYPVSPRPFSSPSMSPSHGMNIHNLASGKGSTAHFSGFESCSNGVISNKAHQSYCHSKHQSS NLNVPELNSINMSRSQQVNNFTSNDVDMETDHYSNGVGETSSNGFLNGSSKHDHEMEDCDTEMEV DSSQLRRQLCGGSQAAIERMIHFGRELQAMSEQLRRECGKNTANKKMLKKPTICQSNLHLP	
56	Uncharacterized protein	A0A4X1V4L3	No data	GO:0005515	Match to NLHLP at 1654<br>MASRERLFELWMLYCAKKDPDYLKLWLDNFVSSYEQFLDVDFEKLPTRVDDVPPGISLLPDNILQVLR SQLLQCVQKMADGLEEEQQALSILLVKFFIILCRNLSNVEEIGTCSYINHVITMTTLYIQQLKSKKKEKE LADQTSIEEFVIHALAFCESLYDPYRNWRHRISGRILSTVEKSRQKYKPASLTVEFVPFFYQCFQESEHL KESLKCCLLHLFGAIVAGGQKNALQAISPATMEVLMRVLADCDSWEDGNPEEVGRKVELTLKCLTE VVHILLTSSSDQRQVETSSILENYFKLLNSDHSALPNQRRSRQWESRFIALQIKMLNTITAMLDCTDRP VLQAIFLNSNCFEHLIRLLQNCKVFQGQLDCLAVSAIQALTAVMNKSPAAKEVFKERIGYTHMFEVL KSLGQPPLELLKELMNMAVEGDHTSVGILGISNVQPLLLLIQWLPEIESHDLQIFISDWLKRICCINRQS RTTCVNANMGIRIIETLDSHSSLHRTCAENLIALHGSLGSQSVSSEEIRQLLRLLRVDEPEYIHPYITPVTR AILTMARKQSLESALQYFNLSHSMAGISVPSIQKWPGSAFSFNAWFCLDQDQLTLGSANKGGKRKQL YSFFTGSGMGFEAFITHSGTLVVAVCTKREYATVMLPDHSFCDSLWHNITIVHMPGKRPFGQSLVYIY DNGQQKVYAPLRFPAMNEPFISCCIGSAGQRTTTPPPSQIPDPPFSSPITPHRTSFGGILSSASWGGTVEK SKLITKLISAGTQDSEWGCPTSLEGQLGSVIIFSEALQPPQVKALYLAGPNCLSPWKFQESDMADLPAN VLLHYTAKACKNSICLDLSTNCLHGRLTGNKVVNWDIKDIINCIGGLNVLFPLMEQIRHFGEGQIPEG PRENTVSELITPVEGDWVVLTSTKASESRLERNLIATFILIVKHFIQRHPINQDNLIHSHGVAVLGALLQ KVPSTLMDVNVLMAIQLLIEQVSLEKNMQLLQQMYQYLLFDFRIWNRGDFPFRIGHIQYLSTIIKDSRR VFRKKYGVQFLLDTLRIYYGSDCKYNELSLDDIRTIRTSLYGLIKYFLCKGGTHEEIQSIVGYIAAISEEEQ LIGILDILFSLLHTSPTRGQLFLLLFEPGNADILYALLLNQKYSDRLREIIFKVMEQMLKCTNVYERSKQR IRLREVGYSGLGLLLNEAPVNTSLIKSLTNQIINTDPAINFKDLLSVVYISHRACVNVRVVICRKILQTLQ SQPDAAHQISQQVGWQDTLVRLFLKANFENGNTPHKHIRTILMKDSDKNIATEDIKRSFDEKTDEEKI SSFASAHVSSDQWSLEDRHSLDSNTPLFQEDSSVGELSFKSENQEEFWHSNPSHLSLDLSGIDSYELSDS GSQMPDSLPSTPSPIESAKSFSVQSNKESSVTNDMGFSDDFTLLESQERCEEELLQLLTTILNYVMCKGL EKSDDDTWIERGQVFSALTKPGISSELLHPPDEIKLILLQKMLEWAVTENREAKINPVTAENALRLMLII QDFLQSEGLVNSNMWTEKLLEDMMLLFDSLSVWYSESPVWVKLSQIQIQLLLGFIGRGNLQVCAMAS AKLNTLLQTKVIENQDEACYILGKLEHVLRQSIKEQTEIYSFLIPLVRTLVSKIYELLFMNLHLPSLPFAN GSSSFFEDFQEYCSSNEWQVYIEKYIVPYMKQYETHTFYDGHESMALYWKDCYEALMVNMHKRDRE GGESKLKFQEFFVEPFNRKARQENLRYNNMLKQLSSQQLATLRRWKAIKLYLTCERGPWAERKQKPI HWKLANVENYSRMRLKLVPNYNFKTHDEASALRDNLGIQHSQPSSDSLLLEVVKQVKVSDMDEDKL DLPEEEITTRVNIDEKEEQDQKEKLVLSEDCELITIIDVIPGRLEITTQHIYFYDGSIEKEDGVGFDFKWPH	90% with A0A287AJ02 (46), F1SHE6 (49), A0A287BFX1 (50), A0A286ZRL3 (51), A0A4X1V4K3 (53), A0A4X1V6V9 (54) and A0A480E999 (45)

					SQVREIHLRRYNLRRSALEIFHVDQSNYFLNFKKEVRNKVYSRLLSLHSPNSYGTRSPQELFKTSGLTQK<br>WVNREISNFDYLIQLNTMAGRTYNDLAQYPVFPWILQDYTSEELDLNNPSVFRDLSKPIGVVNDKNA<br>KAMREKYENFEDPMGTIDKFHYGTHYSNSAGVMHYLIRTEPFTTLHIQLQSGRFDCADRQFHSIPAT<br>WQALMDNPYDVKELIPEFFYFPEFLENQNKFNLGRLQVSKEVVNDVILPKWAKSAEDFIYKHRKALE<br>SEYVSAHLHEWIDLIFGYKQRGPAAVEALNVFYYCSYEGAVDLDALTDEKERKALEGMINNFGQTPC<br>QLLKEPHPSRLSAEEAVQKQTKTDTSTLNLFQHLPELKSFFIEGISDGIPLVKAIVPKNQSRSFMSQGSPE<br>LLITVSMNYVIGTHGWLPYDRTISNYFTFIKDQTVTNPKTQRVMNGPFAPGLEITSKLFIVSHDAKLLFS<br>AGHWDNSIQVMSLTKGKIISHHIRHMDIVTCLATDYCGIHLISGSRDTTCMIWQITQQGGVPVGLASK<br>PFQILYGHTDEVLSVGISTELDMAVSGSRDGTVIIHTIQKGQYMRTLRPPCESSLLLTIPSLAISWEGHIVI<br>YSSIEERTTLKDKNALHLFSVNGKYLGSQVLKEQVSDMCIIGEHIVTGSLQGFLSIRDLHSLNLSINPLA<br>MRLPIHCVCVTKEHSHILVGLEDGKLIVVGVGKPAEMRSGQLSRKLWGSSKRLSQISAGETEYNTQDS<br>K	
57	Uncharacterized protein	A0A4X1VB30	No data	The same (13)	Match to NLHLP at 27<br>MVLVGLLLLLTLLAGARLLWGQWKLRNLHLPPLVPGFLHLLQPNLPIYLLGLTQRLGPIYRLRLGLQD<br>VVVLNSKRTIEEALVRKWVDFAGRPQIPSYKLASQHCPDISLGDYLFWKAHKKLTRSALLLGVRSSME<br>PRVEQLTQEFCERMRAQAGTPVTIQKEFSVLTCSIICCLTFGDKVKEDTLVHALHDCVQDLMKTWEH<br>WSIQILDMVPFLRFFPSPGLRRLKQAIENRDHLVEKQLRRHKESMVAGQWRDMLDYMLQEAGRQRV<br>EEGQGQLLEGHVHMSVVDLFIGGTETTANTLSWAVVYLLHHPEIQWRLQEELDRELGPGAAGSRVP<br>YKDRARLPLLNATIAEVLRLRPVVPLALPHRATRPSSIFGYDIPEGTVVIPNLQGAHLDETVWEQPHEF<br>RPDRFLAPGANPSALAFGCGARVCLGEPLARLELFVVLVQLLQAFTLLPPEGALPSLQPHPHSGINLK<br>VQPFQVRLQPRGGRAQGPGEHL	90% with P15540 (1), A0A5G2QLE9 (47), Q6B7Q1 (10), Q6B7P7 (11) and A5A8W5 (14)
**FVAPW**						
1	Metalloproteinase inhibitor 1	P35624	No data	GO:0005125<br>GO:0008083<br>GO:0046872<br>GO:0008191<br>GO:0002020<br>GO:0071492<br>GO:0043086<br>GO:0010951<br>GO:0051045<br>GO:0008284<br>GO:2001044<br>GO:0034097<br>GO:0009725<br>GO:0010033	Match to FVAPW at 124<br>MSPFAPLASGILLLLWLTAPSRACTCVPPHPQTAFCSSDLVIRAKFVGAPEFNQTASYKRYEIKMTKMF<br>KGFNALGDAPDIRFIYTPAMESVCGYFHRSQNRSQEFLIAGQLWNGHLHITTCSFVAPWNSLSSAQR<br>QGFTEIYAAGCEECTVFPCTSIPCKLQSDTHCLWTDQLLTGSDKGFQSRHLACMPREPGMCTWQSLR<br>PRVA	
2	Metalloproteinase inhibitor 1	A0A4X1T867	Medium in heart left ventricle	GO:0005125<br>GO:0008083<br>GO:0008191<br>GO:0008270<br>GO:0071492	Match to FVAPW at 124<br>MAPFAPLASGILLLLWLTAPSRACTCVPPHPQTAFCSSDLVIRAKFVGAPEFNQTASYKRYEIKMTKM<br>FKGFNALGDAPDIRFIYTPAMESVCGYFHRSQNRSQEFLIAGQLWNGHLHITTCSFVAPWNSLSSAQR<br>QGFTEIYAAGCEECTVFPCTSIPCKLQSDTHCLWTDQLLTGSDKGFQSRHLACMPREPGMCTWQSLR<br>PRVA	90% with P35624 (1)

				GO:0002248<br>GO:0051045<br>GO:1905049<br>GO:1901164<br>GO:0008284<br>GO:2001044		
3	Metalloproteinase inhibitor 1	A0A4X1TA94	No data	GO:0008083<br>GO:0046872<br>GO:0008191	Match to FVAPW at 203<br>MAPFAPLASGILLLLWLTAPSRACTCVPPHPQTAFCSSDLVIRAKFVGAPEFNQTASYKRYEIKMTKM<br>FKGFNALGDAPDIRFIYTPAMESVCGYFHRSQNRSQEFLIAGEAPPSRPLPGNRKGGEALREGSQLKW<br>EPPQSQPIRRLGLCPPGGGSVLLGTPIGSHPAPGSANQKPPVDILPHPHSHPGQLWNGHLHITTCSFV<br>APWNSLSSAQRQGFTEIYAAGCEECTVFPCTSIPCKLQSDTHCLWTDQLLTGSDKGFQSRHLACMPRE<br>PGMCTWQSLRPRVA	
4	Metalloproteinase inhibitor 1	F1RWV2	No data	GO:0005125<br>GO:0008083<br>GO:0008191<br>GO:0008270<br>GO:0002020<br>GO:0071492<br>GO:0002248<br>GO:0010951<br>GO:0051045<br>GO:1905049<br>GO:1901164<br>GO:0008284<br>GO:2001044<br>GO:0034097<br>GO:0009725<br>GO:0010033	Match to FVAPW at 124<br>MAPFAPLASGILLLLWLTAPSRACTCVPPHPQTAFCSSDLVIRAKFVGAPEFNQTASYKRYEIKMTKM<br>FKGFNALGDAPDIRFIYTPAMESVCGYFHRSQNRSQEFLIAGQLWNGHLHITTCSFVAPWNSLSSAQR<br>QGFTEIYAAGCEECTVFPCTSIPCKLQSDTHCLWTDQLLTGSDKGFQSRHLACMPREPGMCTWQSLR<br>PRVA	100% with A0A4X1T867 (2); 90% with P35624 (1) A0A4X1T867 (2)
